# Sustained Survival Benefit in Recurrent Medulloblastoma by a Metronomic Antiangiogenic Regimen: A Nonrandomized Controlled Trial

**DOI:** 10.1001/jamaoncol.2023.4437

**Published:** 2023-10-26

**Authors:** Andreas Peyrl, Monika Chocholous, Magnus Sabel, Alvaro Lassaletta, Jaroslav Sterba, Pierre Leblond, Karsten Nysom, Ingrid Torsvik, Susan N. Chi, Thomas Perwein, Neil Jones, Stefan Holm, Per Nyman, Helena Mörse, Anders Öberg, Liesa Weiler-Wichtl, Ulrike Leiss, Christine Haberler, Maresa T. Schmook, Lisa Mayr, Karin Dieckmann, Marcel Kool, Johannes Gojo, Amedeo A. Azizi, Nicolas André, Mark Kieran, Irene Slavc

**Affiliations:** 1 Department of Pediatrics and Adolescent Medicine, Medical University of Vienna, Vienna, Austria; 2 Comprehensive Center for Pediatrics, Medical University of Vienna, Vienna, Austria; 3 Childhood Cancer Centre, Queen Silvia Children’s Hospital, Sahlgrenska University Hospital, Gothenburg, Sweden; 4 Institute of Clinical Sciences, Sahlgrenska Academy, University of Gothenburg, Gothenburg, Sweden; 5 Department of Pediatric Neuro-Oncology, Hospital Infantil Universitario Niño Jesús, Madrid, Spain; 6 Pediatric Oncology Department, University Hospital Brno, Brno, Czech Republic; 7 Pediatric Oncology Unit, Oscar Lambret Comprehensive Cancer Center, Lille, France; 8 Centre Léon Bérard, Institut d’Hématologie et d’Oncologie Pediatrique, Lyon, France; 9 Department of Paediatrics and Adolescent Medicine, Rigshospitalet, Copenhagen, Denmark; 10 Department of Paediatric and Adolescent Medicine, Haukeland University Hospital, Bergen, Norway; 11 Department of Pediatric Neuro-Oncology, Dana-Farber Cancer Institute, Boston, Massachusetts; 12 Division of Pediatric Hemato-Oncology, Department of Pediatrics and Adolescent Medicine, Medical University of Graz, Graz, Austria; 13 Kinderonkologie, Salzburger Universitätsklinikum, Salzburg, Austria; 14 Department of Pediatric Hematology and Oncology, Karolinska University Hospital, Stockholm, Sweden; 15 Department of Paediatrics, Linköping University Hospital, Linköping, Sweden; 16 Pediatric Cancer Center, Skane University Hospital, Lund, Sweden; 17 Department of Pediatrics, Uppsala University, Uppsala, Sweden; 18 Department of Neurology, Medical University of Vienna, Vienna, Austria; 19 Department of Biomedical Imaging and Image-Guided Therapy, Division of Neuroradiology and Musculoskeletal Radiology, Medical University of Vienna, Vienna, Austria; 20 Department of Radio-Oncology, Medical University of Vienna, Vienna, Austria; 21 Hopp Children’s Cancer Center (KiTZ), Heidelberg, Germany; 22 Division of Pediatric Neurooncology, German Cancer Research Center (DKFZ), German Cancer Consortium (DKTK), Heidelberg, Germany; 23 Princess Máxima Center for Pediatric Oncology, Utrecht, the Netherlands; 24 Départment of Pediatric Oncology, Assistance Publique-Hopitaux de Marseille, Marseille, France; 25 Aix Marseille University, Cancer Research Center of Marseille, Marseille, France

## Abstract

**Importance:**

Medulloblastoma recurrence in patients who have previously received irradiation has a dismal prognosis and lacks a standard salvage regimen.

**Objective:**

To evaluate the response rate of pediatric patients with medulloblastoma recurrence using an antiangiogenic metronomic combinatorial approach (Medulloblastoma European Multitarget Metronomic Anti-Angiogenic Trial [MEMMAT]).

**Design, Setting, and Participants:**

This phase 2, investigator-initiated, multicenter nonrandomized controlled trial assessed 40 patients with relapsed or refractory medulloblastoma without a ventriculoperitoneal shunt who were younger than 20 years at original diagnosis. Patients were enrolled between April 1, 2014, and March 31, 2021.

**Interventions:**

Treatment consisted of daily oral thalidomide, fenofibrate, celecoxib, and alternating 21-day cycles of low-dose (metronomic) oral etoposide and cyclophosphamide, supplemented by intravenous bevacizumab and intraventricular therapy consisting of alternating etoposide and cytarabine.

**Main Outcomes and Measures:**

The primary end point was response after 6 months of antiangiogenic metronomic therapy. Secondary end points included progression-free survival (PFS), overall survival (OS), and quality of life. Adverse events were monitored to assess safety.

**Results:**

Of the 40 patients (median [range] age at treatment start, 10 [4-17] years; 25 [62.5%] male) prospectively enrolled, 23 (57.5%) achieved disease control after 6 months of treatment, with a response detected in 18 patients (45.0%). Median OS was 25.5 months (range, 10.9-40.0 months), and median PFS was 8.5 months (range, 1.7-15.4 months). Mean (SD) PFS at both 3 and 5 years was 24.6% (7.9%), while mean (SD) OS at 3 and 5 years was 43.6% (8.5%) and 22.6% (8.8%), respectively. No significant differences in PFS or OS were evident based on molecular subgroup analysis or the number of prior recurrences. In patients demonstrating a response, mean (SD) overall 5-year PFS was 49.7% (14.3%), and for patients who remained progression free for the first 12 months of treatment, mean (SD) 5-year PFS was 66.7% (16.1%). Treatment was generally well tolerated. Grade 3 to 4 treatment-related adverse events included myelosuppression, infections, seizures, and headaches. One heavily pretreated patient with a third recurrence died of secondary acute myeloid leukemia.

**Conclusions and Relevance:**

This feasible and well-tolerated MEMMAT combination regimen demonstrated promising activity in patients with previously irradiated recurrent medulloblastoma. Given these results, this predominantly oral, well-tolerated, and outpatient treatment warrants further evaluation.

**Trial Registration:**

ClinicalTrials.gov Identifier: NCT01356290

## Key Points

**Question:**

What is the outcome of an antiangiogenic metronomic combinatorial regimen in pediatric patients with recurrent or refractory medulloblastoma?

**Findings:**

In this single-arm, phase 2 nonrandomized controlled trial of 40 patients with previously irradiated recurrent or refractory medulloblastoma, a response rate of 45% was achieved. Six patients achieved a complete response, 9 patients achieved a partial response, and 5 patients had stable disease.

**Meaning:**

The use of an antiangiogenic metronomic combinatorial regimen warrants further investigation.

## Teaser

This nonrandomized controlled trial evaluates the activity and toxicity profile of an antiangiogenic metronomic combinatorial regimen in pediatric patients with relapsed or refractory medulloblastoma.

## Introduction

Medulloblastoma, an aggressive embryonal tumor arising in the cerebellum or, less frequently, in the dorsal brain stem, is one of the most common malignant central nervous system tumors in children.^1^ Four clinically relevant molecular subgroups—WNT (wingless-related integration site), SHH (sonic hedgehog signaling molecule), group 3, and group 4—are known^2^ and have been differentiated into additional subtypes.^3,4,5^ Unfortunately, identification of these subgroups has not yet translated into more effective targeted therapies. Surgery, craniospinal irradiation, and multiagent chemotherapy remain the standard up-front treatment approach for most patients. Although this treatment is highly effective for most patients, approximately 30% of patients will experience relapse of their disease, often metastatic at the time of relapse, and most will eventually die of their disease.^6,7,8,9,10,11,12^ A widely used treatment strategy for recurrent medulloblastoma is the combination of temozolomide and irinotecan,^13^ which was recently augmented with bevacizumab.^14^ This treatment regimen was well tolerated and had an acceptable response rate but failed to lead to sustained responses in patients who had previously received irradiation.

The advantage of metronomic antiangiogenic therapies targeting the tumor microenvironment is that even drugs to which tumors have previously been exposed at high concentrations and have developed resistance can be used. For example, etoposide and cyclophosphamide given at low, long-term dosing to target normal cells that support tumor proliferation, such as endothelium, can produce substantial inhibition of tumor growth, even in tumors made highly resistant to these drugs.^15,16,17^ Similar effects can be observed using other inhibitors of endothelial cell function, including cyclooxygenase 2 inhibitors, peroxisome proliferator–activated receptor α agonists, and thalidomide.^18,19,20^

A retrospective observational study was recently published of an alternative strategy to treat recurrent medulloblastoma, using a modified 5-drug oral metronomic antiangiogenic therapy.^21^ On the basis of this preliminary experience,^22^ we evaluated the activity and toxicity profile of this combinatorial approach in a phase 2 trial (Medulloblastoma European Multitarget Metronomic Anti-Angiogenic Trial [MEMMAT]) in pediatric patients with previously irradiated recurrent or refractory medulloblastoma.

## Methods

### Study Design and Patients

MEMMAT was a prospective, international, multicenter, single-arm, phase 2 study that included sites in Europe and the US. This academic trial depended on local institutions to generate funding to perform the study. The trial was conducted in accordance with the Declaration of Helsinki^23^ and in compliance with the International Conference on Harmonisation Good Clinical Practice and local regulatory ethics committee guidelines. Written informed consent was required from parent(s) or legal guardian(s) and/or the patients before study enrollment. The Transparent Reporting of Evaluations with Nonrandomized Designs (TREND) reporting guidelines were followed. The trial protocol is available in Supplement 1.

Patients were enrolled from April 1, 2014, to March 31, 2021. Eligible patients were younger than 20 years at original diagnosis, with a histologically confirmed medulloblastoma diagnosis and a documented relapse on magnetic resonance imaging (MRI), biopsy, or cerebrospinal fluid (CSF) cytologic testing. There was no limit to the number of previous relapses or any restrictions to the types of previous therapies administered. Acceptable organ function and bone marrow recovery and a Karnofsky Performance Scale Index score or Lansky Play-Performance Scale score of 50% or higher were required (lower Karnofsky and Lansky scores indicate functional impairment). MEMMAT did not collect data on race or ethnicity. At the time of enrollment, patients were permitted to undergo additional surgical resection. Patients dependent on ventriculoperitoneal shunts were excluded because of the anticipated difficulties in providing adequate intraventricular therapy. The medulloblastoma molecular group was determined by DNA methylation profiling according to previously described methods.^24^ Forty-one patients met the eligibility criteria and were enrolled, and 1 dropped out, leaving an evaluable population of 40 patients.

### Study Treatment

Treatment consisted of daily oral thalidomide, fenofibrate, celecoxib, and alternating 21-day cycles of oral etoposide and cyclophosphamide, supplemented by intravenous bevacizumab and intraventricular therapy via an Ommaya reservoir consisting of alternating etoposide and liposomal or aqueous cytarabine. For an overview of the dosing schedule and dose modifications, see eTables 1 and 2 in Supplement 2. Concomitant pneumocystis prophylaxis was recommended. Treatment was given for 12 months and was continued for an optional second year without oral etoposide or cyclophosphamide and with extended intervals of intraventricular therapy, depending on response and tolerability.

### Response Assessments

Magnetic resonance imaging with and without gadolinium enhancement was performed within 2 weeks before the start of protocol therapy. In case of surgery, postoperative MRI was required within 72 hours after surgery. Six months after the start of antiangiogenic treatment, MRI was mandatory to assess response. A CSF cytologic test was performed once at each cycle of intraventricular therapy. Continuing response or stable disease was confirmed every 3 months until disease progression or study discontinuation. The MRIs were collated and centrally reviewed by an experienced panel of pediatric neuroradiologists and pediatric neurooncologists (A.P. and M.S.).

#### Response Criteria

Complete response (CR) was defined as the total disappearance of all radiologic evidence of tumor, determined by 2 observations not less than 4 weeks apart, and no evidence of malignant cells in the CSF. No evidence of disease (NED) was defined as no recurrence or appearance of new lesions and no malignant cells in the CSF in a patient with complete resection and no measurable disease after surgery, with NED continuing for at least 6 months. Partial response (PR) was defined as regression of at least 50% of all tumor size (the sum of the products of all measured lesions), determined by 2 observations not less than 4 weeks apart. No simultaneous progression of any lesion or the appearance of new lesions may have occurred. Nonmeasurable lesions (eg, diffuse leptomeningeal spread) must have remained stable or regressed for this category. Patients whose CSF results were negative for disease were required to continue to test negative. Stable disease was regression of less than 50% of tumor size or progression less than 25% of at least 4 weeks’ duration, without appearance of new lesions. Progressive disease was worsening of disease, evidenced by enlargement of any existing lesion(s) of 25% or more or appearance of new lesions or the manifestation of new CSF-positive disease.

Health-related quality of life (QOL) data were collected using the KINDL questionnaire in the respective national language at study enrollment and after 6 months. The questionnaire consists of 24 Likert scale items, with 4 items each representing 1 of the following 6 scales: physical well-being, emotional well-being, self-esteem, family, friends, and everyday functioning (school or nursery school or kindergarten). Age-specific self-report versions for children and adolescents and a proxy version for parents were applied. The scale scores and the total score were compared with reference scores of a norm sample of the same age groups.^25^

Patients were followed up for survival after completion or discontinuation of study treatment. Safety was evaluated and graded according to the National Cancer Institute’s Common Terminology Criteria for Adverse Events (CTCAE), version 4.0. Adverse events were recorded at study visits and for at least 28 days after the last dose of study treatment.

### Statistical Analysis

Data cutoff was on March 7, 2022. Trial enrollment for this phase 2 cohort was planned for 40 patients (1 − β = 0.90; estimated dropout rate, 5%). The clinically sufficient response rate was specified with π1 = 35%; the clinically insufficient response rate was specified with π0 = 15%. In the primary efficacy analysis, tumor responses of all patients were determined by central review and used as the primary assessment in the overall response analysis. The primary end point of response was estimated using the minimax 3-stage design for phase 2 oncology clinical trials with 2 interim analyses (eTable 3 in Supplement 2).^26^ Binomial proportion of best overall response of a confirmed NED, CR, or PR was reported using a 2-sided multiple testing procedure with α = .05. Both overall survival (OS) and progression-free survival (PFS) were described using the Kaplan-Meier method, and median OS and PFS were calculated using reversed Kaplan-Meier estimator; median times and 95% CIs were calculated. Statistical analyses were conducted using SPSS software, version 27 (SPSS Inc).

## Results

### Study Patients

**Figure 1.**
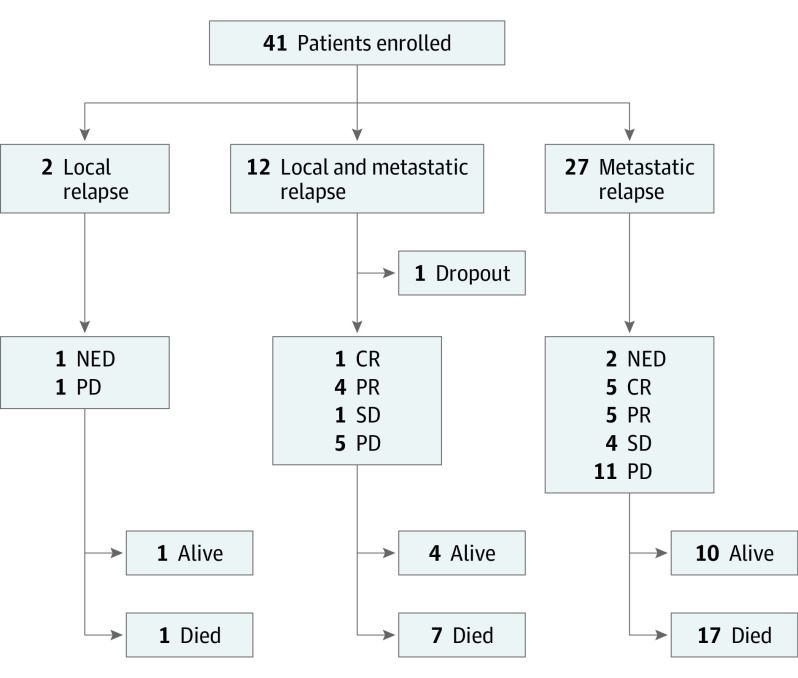
Study Flow Diagram CR indicates complete response; NED, no evidence of disease; PD, progressive disease; PR, partial response; SD, stable disease.

Baseline demographic and disease characteristics of the 40 analyzed patients (median [range] age at treatment start, 10 [4-17] years; 25 [62.5%] male and 15 [37.5%] female) are summarized in Table 1. All patients had received irradiation as part of their previous treatment. Median (range) time to relapse was 20 (1-57) months. Eight patients experienced a second relapse and 2 patients a third relapse. Seven of these patients received temozolomide, irinotecan, bevacizumab, or a combination of those drugs to treat previous relapses. The pattern of relapse was local (2 [5.0%]), metastatic (27 [67.5%]), or combined local and metastatic (11 [27.5%]) (Figure 1). No association between molecular group at diagnosis and individual relapse pattern was found. Twelve patients underwent surgical resection before enrollment, with 4 gross total resections and 8 partial resections. Four patients received focal reirradiation after completion of the 6-month response MRI. *MYC(N)* amplification was identified in 4 of the 40 patients: 3 (*MYC* [OMIM 190080]) in group 3 and 1 (*MYCN* [OMIM 164840]) in SHH, similar to previous studies.^9,10^

**Table 1.** Baseline Demographic and Disease Characteristics^a^

Characteristic	Finding (N = 40)
Sex	
Female	15 (37.5)
Male	25 (62.5)
Molecular subgroup at original diagnosis	
WNT	1 (2.5)
SHH	4 (10.0)
Group 3	12 (30.0)
Group 4	23 (57.5)
*MYC/MYCN* amplification	
No	36 (90.0)
* MYC*	3 (7.5)
* MYCN*	1 (2.5)
No. of recurrences at study entry	
1	29 (72.5)
2	9 (22.5)
3	2 (5.0)
Age at MEMMAT start, median (range), y	10 (4-17)
Pattern of current relapse	
Local	2 (5.0)
Metastatic	28 (70.0)
Combined local and metastatic	10 (25.0)

Abbreviation: MEMMAT, Medulloblastoma European Multitarget Metronomic Anti-Angiogenic Trial; SHH, sonic hedgehog signaling molecule; WNT, wingless-related integration site.

^a^ Data are presented as number (percentage) of patients unless otherwise indicated. Data on race and ethnicity were not collected by MEMMAT.

### Efficacy

Among the 40 patients, 23 (57.5%) achieved disease control (NED, CR, PR, and stable disease) after 6 months of treatment, whereas 17 patients (42.5%) discontinued treatment because of disease progression within the first 6 months. A total of 16 patients (40.0%) received the MEMMAT treatment for at least 1 year. Three patients had a complete resection of their relapse and remained with NED thereafter (7.5%). Best response was CR in 6 patients (15.0%), PR in 9 patients (22.5%), and stable disease in 5 patients (12.5%). Objective response rate, defined as ongoing NED after complete resection, CR, or PR, was 45%.

**Figure 2.**
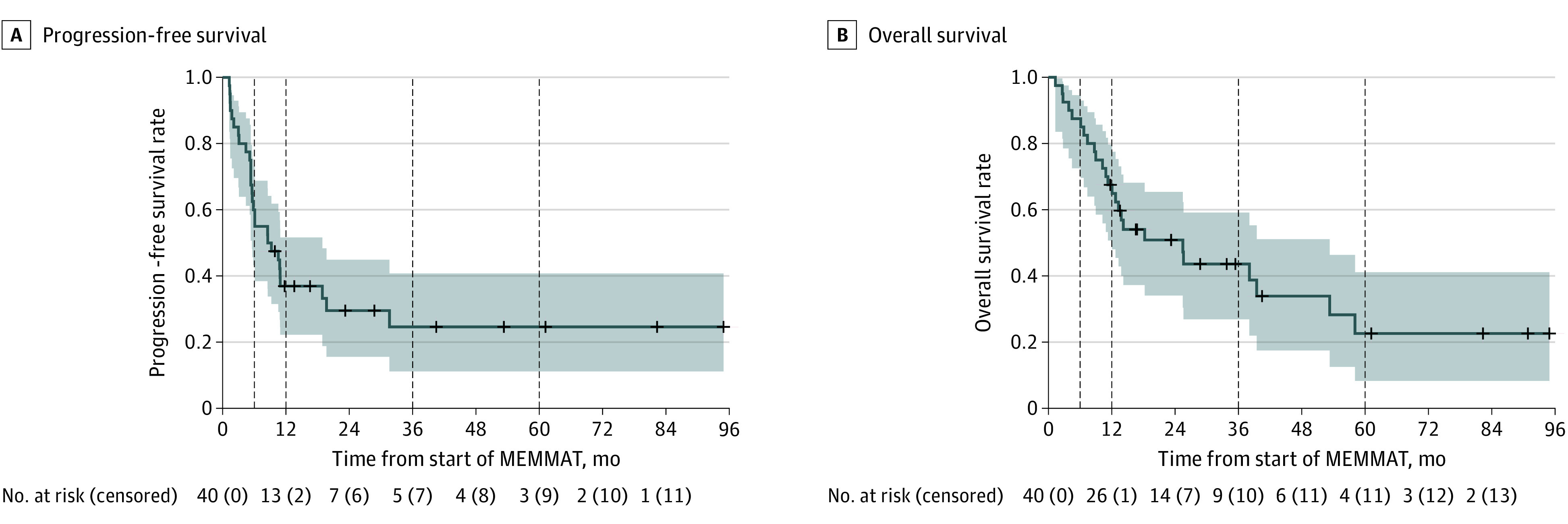
Kaplan-Meier Estimates of Survival Outcomes of the 40 Study Participants The shaded areas represent 95% CIs; vertical dashed lines indicate the 6-month, 12-month, 36-month, and 60-month time points; and crosses represent censored participants. MEMMAT indicates Medulloblastoma European Multitarget Metronomic Anti-Angiogenic Trial.

**Figure 3.**
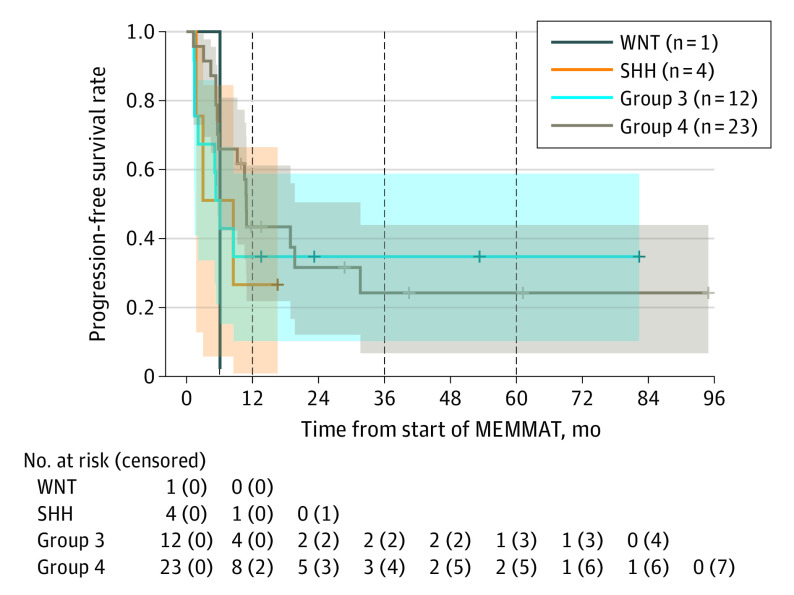
Progression-Free Survival After 12 Months for Medulloblastoma Subgroup Distribution The shaded areas represent 95% CIs; vertical dashed lines indicate the 12-month, 36-month, and 60-month time points; and crosses represent censored participants. MEMMAT indicates Medulloblastoma European Multitarget Metronomic Anti-Angiogenic Trial; SHH, sonic hedgehog signaling molecule; and WNT, wingless-related integration site.

Median follow-up time was 40.5 months (range, 1.3-94.9 months), and mean (SD) PFS at both 3 and 5 years was both 24.6% (7.9%). Mean (SD) OS was 43.6% (8.5%) at 3 years and 22.6% (8.8%) at 5 years. Median PFS and OS were 8.5 months (range, 1.7-15.4 months) and 25.5 months (range, 10.9-40.0 months), respectively (Figure 2). Two patients died of events without progression of the investigated disease. No significant differences in PFS or OS were evident regarding the molecular groups (Figure 3; eTable 4 in Supplement 2), the number of relapses, or the intraventricular treatment with liposomal cytarabine or aqueous cytarabine (eFigures 1-3 in Supplement 2).

In an exploratory analysis of patients who had disease control at 6 months, median PFS was 31.6 months (range, 11.0-52.2 months), and 5-year mean (SD) PFS was 42.8 (12.4) months. In the 18 patients (45.0%) who demonstrated any response (NED, CR, or PR), median PFS was 31.6 months (range, 11.0-52.2 months) and mean (SD) 5-year PFS was 49.7 (14.3) months. In the 13 patients (32.5%) who had no progression after 12 months of treatment, mean (SD) 5-year PFS rate was 66.7% (16.1%). Fifteen patients remained alive for a mean of 39 months (range, 12-95 months) after initiation of MEMMAT treatment, 12 of whom were progression free (eFigures 4-6 in Supplement 2).

### Safety and QOL

The most frequently reported adverse events of any grade were hematologic disorders (Table 2). One heavily pretreated patient who presented with a third relapse died of secondary acute myeloid leukemia 10 months after starting MEMMAT treatment, having received etoposide, temozolomide, and irinotecan as part of his prior therapy. A complete list of all treatment-related adverse effects is shown in eTable 5 in Supplement 2.

**Table 2.** Treatment-Related Adverse Events in the Study Patients

Adverse event	No. (%) of patients (N = 40)		
	Grade 3	Grade 4	Total
Hematologic			
Anemia	5 (12.5)	0	5 (12.5)
Leukopenia	2 (5.0%)	8 (20.0)	10 (25.0)
Lymphopenia	0	3 (7.5)	3 (7.5)
Neutropenia	3 (7.5)	14 (35)	17 (42.5)
Platelet count decreased	0	4 (10.0)	4 (10.0)
Neurologic			
Fatigue	1 (2.5)	0	1 (2.5)
Headache	2 (5.0)	0	2 (5.0)
Hearing impairment	0	1 (2.5)	1 (2.5)
Seizure	2 (5.0)	0	2 (5.0)
Infection			
Cerebellitis	1 (2.5)	0	1 (2.5)
Chemical arachnoiditis	0	1 (2.5)	1 (2.5)
Febrile neutropenia	2 (5.0)	0	2 (5.0)
Infection, not otherwise specified	3 (7.5)	1 (2.5)	4 (10.0)
Meningitis	2 (5.0)	0	2 (5.0)
Mucositis	1 (2.5)	0	1 (2.5)
Sepsis	0	1 (2.5)	1 (2.5)
Urinary tract infection	1 (2.5)	0	1 (2.5)
Laboratory			
Elevated liver enzymes	2 (5.0)	0	2 (5.0)
Hyponatremia	3 (7.5)	0	3 (7.5)
Various			
Hypertension	2 (5.0)	0	2 (5.0)
Proteinuria	2 (5.0)	0	2 (5.0)
Wound dehiscence	1 (2.5)	0	1 (2.5)

The QOL results measured by the KINDL questionnaire indicated a medium or low QOL before initiation of protocol therapy. Quality of life did not further decrease considerably once therapy was initiated and slightly improved, although results were heterogeneous (eTable 6 in Supplement 2).

## Discussion

Effective treatment for recurrent medulloblastoma remains a significant unmet need, with no approved therapies and only rare long-term survivors. The metronomic antiangiogenic drug combination with intraventricular therapy used in MEMMAT significantly prolonged survival and was able to produce durable responses in patients without a ventriculoperitoneal shunt who had previously received irradiation. The treatment is predominantly oral, well tolerated, outpatient, and easily adapted in these heavily pretreated patients.

In the past, numerous attempts have been made to treat relapsed medulloblastoma. In some of these studies, significant responses have been observed, but these responses lasted only a short period in patients who had previously received irradiation.^13,14,27^ With a median OS of 25.5 months, our study also compared favorably to the OS of 19 months reported by the Children’s Oncology Group study with the addition of bevacizumab to temozolomide and irinotecan.^14^ Whether this superior result is attributable to the difference in systemic therapy or the addition of intraventricular therapy needs to be determined. Although half of the infants in one study who had not received irradiation as part of their primary treatment could be saved at relapse,^10,28^ subsequent irradiation or myeloablative chemotherapy was not successful in patients previously treated with irradiation, with only a limited number of patients alive at the time of reporting of those often highly selected cohorts.^29,30,31,32^ Most important, the MEMMAT combination enhanced durability of response and facilitated sustained PFS in one-quarter of patients with recurrent medulloblastoma who have undergone heavy pretreatment and previous irradiation.

In accordance with a previous study,^10^ we observed that our exclusively prior-irradiated cohort also experienced early relapses. A post hoc analysis, however, showed very promising 5-year PFS in patients demonstrating a response (49.7%) and for patients who remained progression free the first 12 months of treatment (66.7%), indicating patients who might benefit from this intervention.

MEMMAT differs from conventional treatment regimens because it applies a metronomic antiangiogenic combinatorial approach to target the tumor microenvironment. Metronomic chemotherapy is defined as the long-term administration of chemotherapy at low, minimally toxic doses, without prolonged drug-free breaks designed to treat vascular cells needed to maintain tumor cell proliferation, migration, and metastatic spread. Beyond its antiangiogenic effect, metronomic chemotherapy appears to also affect other cells within the tumor microenvironment that may play important roles by inducing immunogenic pathways that can activate both innate and adaptive immune responses. Metronomic cyclophosphamide, for example, was shown to deplete regulatory T cells in various tumor types and is a common treatment for nephrotic syndrome and lupus nephritis.^33,34,35^

Given the redundancy of mechanisms involved in new blood vessel formation by cancer, growth interference via multiple pathways is required for induction and maintenance of tumor response. Therefore, MEMMAT combined metronomic chemotherapy with the repurposing of nonchemotherapeutic drugs, as was previously done in the COMBAT (combined oral metronomic biodifferentiating antiangiogenic treatment) regimen.^36,37^ The rationale for combining the 5 oral drugs as well as bevacizumab and intraventricular therapy in MEMMAT has previously been described in detail.^21,22,38,39^

Metronomic chemotherapy has been reported to be better tolerated and to induce less severe adverse effects than conventional chemotherapy. This was also confirmed by MEMMAT, in which this therapy was generally well tolerated in a heavily pretreated cohort in an outpatient setting. The MEMMAT combination was feasible because the patients were adherent even with the frequent necessary visits to the outpatient clinic for intraventricular therapy, and only 1 of all patients dropped out of the study. Most toxic effects were hematologic and consistent with low-dose chemotherapy. Frequent clinical and laboratory checks were used to closely monitor the treatment, and dose modification was added to reduce neutropenic episodes and infections while minimizing prolonged drug-free breaks.

Quality of life during metronomic therapy has rarely been reported but is even more important for patients with an overall poor prognosis.^40,41^ The QOL questionnaires indicated a medium or low QOL at baseline, likely reflecting the heavy burden of prior therapy and associated sequelae from their original diagnosis and/or prior relapses. Furthermore, the significance of another relapse might have further influenced the low baseline QOL at the start of treatment. However, it appears that QOL did not worsen during MEMMAT treatment and slightly improved. Clearly, additional studies in this area of patient outcome are needed to clarify areas that might help improve QOL.

All but 2 patients in our study had disease recurrence at distant central nervous system sites with or without disease in the original tumor bed, a pattern that is consistent with previous observations.^9,11^ Prior studies have shown that surgery in isolated medulloblastoma relapses was associated with improved survival,^8,9,42^ albeit with limited long-term success in patients who had previously received irradiation.^7,8,41^ To avoid denying patients with complete resection access to the study, MEMMAT allowed patients with recurrent medulloblastoma to undergo resection before enrollment, and maintaining NED after a gross total resection was considered a benefit of the treatment combination. However, only 4 patients in our cohort achieved a gross total resection, 2 with local and 2 with metastatic relapse. One of these patients experienced rapid disease progression (3 months) and died of her disease, whereas the other 3 patients are alive, progression free, and without evidence of disease at 41, 29, and 14 months, respectively, after enrollment in MEMMAT.

Although it was not an original objective of the trial, with the recent subclassification of medulloblastoma,^5^ we were interested to see whether outcome differed by group. No association between outcome and group assignment was identified; however, this study was not powered to pick up small differences (Figure 2). This result is consistent with the data from our retrospective series^21^ and the recent Children’s Oncology Group trial for relapsed medulloblastoma,^14^ although others have reported longer median postrelapse survival for patients with group 4 tumors.^11^

### Limitations

There are some important limitations of our study. The patient population reported in this trial, although all having relapsed medulloblastoma after standard up-front surgery, radiation therapy, and chemotherapy, is heterogeneous with regard to their molecular subgrouping, and the study was underpowered to detect true differences between groups. In contrast to a previous publication,^21^ we have excluded patients with a ventriculoperitoneal shunt because of the inherent risk of premature diversion of possibly important intraventricularly administered drugs. The ability to identify which drugs were responsible for problematic toxic effects in this group was thus limited, which impacts our ability to make optimal substitutions going forward. Randomized clinical trials are needed to fully evaluate the efficacy of this metronomic regimen, although this will be difficult because there is no clear standard of care for comparison. Perhaps more importantly, all the drugs in this regimen are commercially available, as are those of other frequently used regimens in relapsed medulloblastoma; thus, there are no industry partners incentivized to support such studies at the current time, and the availability of the drugs might depend on the market situation. Relying on comparisons to other single-arm trials, while less than ideal, remains the best current option for identifying new active treatment regimens. Because the aim of initial treatment is to cure and prevent relapse, figuring out how and for which patients to incorporate this approach into frontline therapy remains a long-term goal.^43,44^

## Conclusions

This nonrandomized controlled trial found that the MEMMAT regimen is a feasible and promising strategy for patients who have previously received irradiation. Given this meaningful clinical benefit in a difficult-to-treat population, further evaluation of the combination is warranted. In the future, an improved understanding of the molecular signature and specific pathways of medulloblastoma groups and subgroups may allow us to add targeted therapies to a MEMMAT backbone.

## Notes

Supplement 1.
Trial Protocol

Supplement 2. **eFigure 1.** OS After 12 Months for Medulloblastoma Subgroup Distribution **eFigure 2.** Kaplan-Meier Estimates of Survival Outcomes Regarding the Number of Relapses **eFigure 3.** Kaplan-Meier Estimates of Survival Outcomes Regarding Intraventricular Liposomal Cytarabine (n = 17) and Aqueous Cytarabine (n = 24) **eFigure 4.** Kaplan-Meier Estimates for PFS of Patients That Exhibited Disease Control (NED+CR+PR+SD) **eFigure 5.** Kaplan-Meier Estimates for PFS of Patients That Exhibited Response (NED+CR+PR) **eFigure 6.** Kaplan-Meier Estimates for PFS of Patients That Showed No Progression After 12 Months of MEMMAT Treatment **eTable 1.** MEMMAT Regimen: Dosing Schedule **eTable 2.** Dose Modifications of the MEMMAT Regimen **eTable 3.** Results and Description of the Interim Analyses **eTable 4.** Responders and Non-responders by Subgroup **eTable 5.** All Treatment-Related AEs in the Study Population (N = 40) **eTable 6.** Quality of Life at the Beginning and After 6 Months of the MEMMAT Protocol

Supplement 3.
Data Sharing Statement
