# Supplementary material for: Sustained Survival Benefit in Recurrent Medulloblastoma by a Metronomic Antiangiogenic Regimen: A Nonrandomized Controlled Trial
Source: JAMA Oncol. 2023 Oct 26;9(12):1688–95. doi: 10.1001/jamaoncol.2023.4437 (PMC10603581; doi:10.1001/jamaoncol.2023.4437)
Supplement: Supplement 1. — Trial Protocol [file jamaoncol-e234437-s001.pdf]

Clinical study protocol

A Phase II study of metronomic and targeted anti-angiogenesis therapy for children with recurrent/progressive medulloblastoma, ependymoma and ATRT

MEMMAT

Medulloblastoma European Multitarget Metronomic Anti-Angiogenic Trial

Protocol authors: Andreas Peyrl [1]
Monika Chocholous [1]
Irene Slavc [1]
Nicholas André [2]
Mark Kieran [3]
Amedeo Azizi [1]

[1] Department of Pediatrics, Medical University of Vienna, Austria
[2] Pediatric Oncology Department, Children Hospital of "La Timone", Marseille, France
[3] Pediatric Neuro-Oncology, Dana-Farber Cancer Institute, Boston, MA, United States

Eudra-CT: 2010-023691-33
ClinicalTrials.gov Identifier: NCT01356290
StudyID: MUV-MEMMAT-01

MEDICAL UNIVERSITY OF VIENNA

Important Contact Information

Austria

Study function	Name, email	Address	Telephone / Fax
Principal investigator	Assoc. Prof. Dr. Andreas Peyrl andreas.peyrl@meduniwien.ac.at	Department of Pediatrics, Medical University of Vienna, Währinger Gürtel 18-20, A-1090 Vienna, Austria	Tel.: +43 1 40400 31750 Fax.: +43 1 40400 61028
Co-Investigator	Univ. Prof. Dr. Irene Slavc irene.slavc@meduniwien.ac.at	Department of Pediatrics, Medical University of Vienna, Währinger Gürtel 18-20, A-1090 Vienna, Austria	Tel.: +43 1 40400 31750 Fax.: +43 1 40400 61028
Co-Investigator	Assoc. Prof. Dr. Amedeo A. Azizi amedeo.azizi@meduniwien.ac.at	Department of Pediatrics, Medical University of Vienna, Währinger Gürtel 18-20, A-1090 Vienna, Austria	Tel.: +43 1 40400 31750 Fax.: +43 1 40400 61028
Co-Investigator	Mag. Dr. Monika Chocholous monika.chocholous@meduniwien.ac.at	Department of Pediatrics, Medical University of Vienna, Währinger Gürtel 18-20, A-1090 Vienna, Austria	Tel.: +43 1 40400 31750 Fax.: +43 1 40400 61028
Co-Investigator	DDr. Johannes Gojo johannes.gojo@meduniwien.ac.at	Department of Pediatrics, Medical University of Vienna, Währinger Gürtel 18-20, A-1090 Vienna, Austria	Tel.: +43 1 40400 31750 Fax.: +43 1 40400 61028
Co-Investigator	Univ. Prof. Dr. Karin Dieckmann karin.dieckmann@meduniwien.ac.at	Department of Radiotherapy, Medical University of Vienna, Währinger Gürtel 18-20, A-1090 Vienna, Austria	Tel.: +43 1 40400 76760 Fax.: +43 1 40400 26660
Co-Investigator	Dr. Maresa Schmook maria.schmook@meduniwien.ac.at	Department of Radiology, Medical University of Vienna, Währinger Gürtel 18-20, A-1090 Vienna, Austria	Tel.: +43 1 40400 48180 Fax.: +43 1 40400 48980
Co-Investigator	Univ. Prof. Dr. Thomas Czech thomas.czech@meduniwien.ac.at	Department of Neurosurgery, Medical University of Vienna, Währinger Gürtel 18-20, A-1090 Vienna, Austria	Tel.: +43 1 40400 25650 Fax.: +43 1 40400 45660
Co-Investigator	Univ. Prof. Dr. Christine Haberler christine.haberler@meduniwien.ac.at	Institute of Neurology, Medical University of Vienna, Währinger Gürtel 18-20, A-1090 Vienna, Austria	Tel.: +43 1 40400 55010 Fax.: +43 1 40400 55110
Co-Investigator	Univ. Prof. Dr. Peter Bauer peter.bauer@meduniwien.ac.at	Center for Medical Statistics, Medical University of Vienna, Währinger Gürtel 18-20, A-1090 Vienna, Austria	Tel.: +43 1 40400 74890
Co-Investigator	Mag. Dr. Michael Weber michael.weber@meduniwien.ac.at	Department of Radiology, Medical University of Vienna, Währinger Gürtel 18-20, A-1090 Vienna, Austria	Tel.: +43 1 40400 57450
Co-Investigator	Univ. Prof. Dr. Martin Benesch martin.benesch@medunigraz.at	Department of Pediatrics, Medical University of Graz, Auenbruggerplatz 34/2, A-8036 Graz, Austria	Tel.: +43 316 385 13485
Co-Investigator	Ass. Prof. PD Dr. Roman Crazzolara roman.crazzolara@i-med.ac.at	Department of Pediatrics I, Medical University of Innsbruck, Anichstrasse 35, 6020 Innsbruck, Austria	Tel.: +43 512 504 23525
Co-Investigator	Dr. Georg Ebetsberger-Dachs georg.ebetsbergerdachs@gespag.at	Landes-Frauen- und Kinderklinik Linz, Krankenhausstraße 26-30, 4020 Linz, Austria	Tel.: +43 5055 46324 290
Co-Investigator	Dr. Agnes Gamper a.gamper@salk.at	Salzburger Universitätsklinikum, Kinderonkologie Salzburg, Müllner Hauptstrasse 48, 5020 Salzburg, Austria	Tel.: +43 662 448257 759

International Study managers

	Name, email	Address	Telephone / Fax
Czech Republic	Jaroslav Sterba, MD<br>jsterb@fnbrno.cz	University Hospital Brno, Pediatric Oncology Department, Cernopolni 9, PC 662 63, Brno, Czech Republic	Phone: +420532234755
Denmark	Karsten Nysom, MD<br>karsten.nysom@regionh.dk	Section 5054, Rigshospitalet, Blegdamsvej 9, DK-2100 København, Denmark	Phone: +45 3545 0809<br>Fax +45 3545 5055
France	Nicholas André, MD, PhD<br>nicolas.andre@ap-hm.fr	Pediatric Oncology Department, Children Hospital of " La Timone ", Bd Jean Moulin 13885 Marseille Cedex 5, France	Phone: +33491388628<br>Fax: +334913820-24
Norway	Ingrid Kristin Torsvik<br>ingrid.kristin.torsvik@helse-bergen.no	Onkologisk-hematologisk seksjon Barneklinikken Haukeland universitetssjukehus, Bergen, Norway	Phone: +4705300<br>Fax: +4755975147
Spain	Alvaro Lassaleta, MD<br>alvaro.lassaletta@salud.madrid.org	Department of Pediatric Hematology and Oncology, Hospital Infantil Universitario Nino Jesus, Av. Menéndes Pelayo 65, Madrid 28009, Spain	Phone +34 915 035938377<br>Fax + 34 93 600 97 82
Sweden	Magnus Sabel, MD<br>magnus.sabel@vgregion.se	Childhood cancer centre (ward 322), Queen Silvia Childrens Hospital, 41685 Gothenburg, Sweden	Phone: +46-31-343 45 22<br>Fax: +46-31-21 54 86
USA, Austin	Virginia Harrod, MD<br>VLHarrod@ascension.org	Dell Children's Medical Group SFC-HEM/ONC, 1301 Barbara Jordan Blvd, Ste 401, Austin, TX 78723, United States	Phone: +512 628 1900<br>Fax: +512 628 1901
USA, Boston	Susan Chi, MD<br>Susan_Chi@dfci.harvard.edu	Pediatric Neuro-Oncology, Dana-Farber Cancer Institute and Boston Children's Hospital, 450 Brookline Avenue, Rm SW331, Boston, MA, United States	Phone: +1617632-4907<br>Fax: +1617632-4897
USA, Chicago	Steward Goldman, MD<br>SGoldman@luriechildrens.org	Ann & Robert H. Lurie Children's Hospital of Chicago, 225 East Chicago Avenue, Mailstop 30, Chicago, IL 60611-2650, United States	Phone: +312 227 4846<br>Fax: +312 227 9372
USA, Grand Rapids	Giselle Saulnier-Sholler, MD<br>Giselle.SaulnierSholler@helendevoschildrens.org	Helen DeVos Children's Hospital, 100 Michigan Street, NE, MC272, Grand Rapids, MI 48503, United States	Phone: +616 267 0334<br>Fax: +616 391 2785
USA, New York	Stergios Zacharoulis, MD<br>sz2764@cumc.columbia.edu	Columbia Institution Medical Center, Department of Pediatric Hematology Oncology, Herbert Irving Pavillon, 168 Fort Washington Ave, New York NY10032, United States	Phone: +212 305 9005<br>Fax: +212 305 9006

Data Monitoring Committee

	Name, email	Address	Telephone / Fax
	Frank Berthold, MD<br>frank.berthold@uk-koeln.de	University Hospital of Cologne, Cologne, Germany	Phone: +49 221 478 6880
	Eric Bouffet, MD<br>eric.bouffet@sickkids.ca	Division of Hematology/Oncology, Hospital for Sick Children, University of Toronto, Toronto, ON, Canada	Phone: 416 813 7457<br>Fax: 416 813 8024

Sponsor

Medical University of Vienna
Department of Pediatrics
Währinger Guertel 18-20
1090 Wien

Monitor

Sabine Breyer
Medical University of Vienna
Department of Pediatrics
Währinger Guertel 18-20
1090 Wien

SAE Reporting (FAX)

FAX +43 1 40400 61028

Signatures

This protocol has been carefully reviewed and agreed to by

Univ. Prof. Dr. Susanne Greber-Platzer, Medical University of Vienna

----
Assoc. Prof. Dr. Andreas Peyrl, Principal Investigator

Univ. Prof. Irene Slavc, Co-Investigator

Mag. Dr. Monika Chocholous, Co-Investigator

Assoc. Prof. Dr. Amedeo Azizi, Co-Investigator

1 Study synopsis

Title	*A phase II study of metronomic and targeted anti-angiogenesis therapy for children with recurrent / progressive medulloblastoma, ependymoma and ATRT*
Study acronym	*MEMMAT (Medulloblastoma European Multitarget Metronomic Anti-Angiogenic Trial)*
Principal investigator:	*Andreas Peyrl*
Sponsor:	*Medical University of Vienna, Department of Pediatrics*
Background	*Patients with relapsed medulloblastoma have a very poor prognosis whether treated with conventional chemotherapy, high-dose chemotherapy with stem cell rescue, irradiation or combinations of these modalities.*<br>*Antiangiogenesis therapy has emerged as a new treatment option in solid malignancies. The frequent delivery of low doses of chemotherapy, referred to as metronomic or antiangiogenic chemotherapy, targets endothelial cells while reducing the toxicity associated with standard dose chemotherapy.*<br>*In this study we will evaluate the use of intravenous bevacizumab every two weeks in combination with five oral drugs (thalidomide, celecoxib, fenofibrate, and alternating cycles of daily low-dose oral etoposide and cyclophosphamide), augmented with alternating courses of intraventricular etoposide and either aqueous or liposomal cytarabine.*
Aim of the study	*The aim of the study is to extend therapy options for children with recurrent or progressive medulloblastoma, ependymoma and atypical teratoid rhabdoid tumor (ATRT), for whom no known curative therapy exists, by prolonging survival while maintaining good quality of life. The primary objective of the MEMMAT trial is to evaluate the efficacy of this multidrug antiangiogenic approach in these heavily pretreated children and young adults as measured by response (CR, PR, SD or persistence of NED after complete resection) 6 months after start of therapy. Additionally, PFS, OS, toxicity, QoL, Performance status, prognostic factors and tumor/angiogenic markers will be examined.*
Design of the clinical trial	*The study is an open label, single arm phase II design.*
Study drugs	*Bevacizumab (intravenous dose of 10 mg/kg every two weeks); thalidomide (3 mg/kg/day daily orally); celecoxib (50 mg – 400 mg orally twice daily); fenofibrate (90 mg/m^2 daily orally); alternating 21-day courses of etoposide (35-50 mg/m^2 daily orally), and cyclophosphamide (2.5 mg/kg daily orally). Intraventricular etoposide (0.5 mg x 5d in one week out of every four weeks), alternating with intraventricular aqueous cytarabine (30 mg >3 years; twice weekly for two weeks out of every four weeks) or liposomal cytarabine (25-50 mg out of every four weeks). Treatment will be given for 12 months duration and should be extended depending on tolerability and response.*
Patients	*Patients who fulfill the eligibility criteria will be evaluated for study participation. All therapeutic decisions (e.g. onset, duration and discontinuation of therapy) need to ensure the safety of the patients at all times.*<br><br>*<u>Inclusion criteria for patients</u>*<br>*Stratum I: Relapsed or progressive medulloblastoma (at least one site of untreated recurrent disease)*<br>*Stratum II: Relapsed or progressive ependymoma (at least one site of untreated recurrent disease)*<br>*Stratum III: Relapsed or progressive ATRT (at least one site of untreated recurrent disease)*<br>*Histological confirmation of medulloblastoma at diagnosis or relapse*<br>*Female or male, aged from 0 to <20 years (at time of original diagnosis)*<br>*Participants must have normal organ and bone marrow function (ALT <5x institutional upper limit of normal, creatinine <1.5x institutional upper limit of normal for age, WBC >1000/mm^3, platelets > 20,000/mm^3. Patients with*

values less than WBC 2000/mm^3 or platelets 50,000/mm^3 will require initiation of treatment with etoposide and cyclophosphamide at a lower starting dose as defined within the protocol
Karnofsky performance status ≥50. For infants and children less than 12 years of age, the Lansky play scale ≥50% will be used
Written informed consent of patients and / or parents

Exclusion criteria for patients

Active infection
VP-shunt dependency
Pregnancy or breast feeding
Conventional chemotherapy or complete irradiation of all disease for current relapse (surgery may be performed before antiangiogenic treatment; patients with sites of disease not irradiated are still eligible for the protocol)
Known hypersensitivity to any of the drugs in the protocol
Active peptic ulcer
Any significant cardiovascular disease not controlled by standard therapy e.g. systemic hypertension
Anticipation of the need for major elective surgery during the course of the study treatment
Any disease or condition that contraindicates the use of the study medication/treatment or places the patient at an unacceptable risk of experiencing treatment-related complications
Non-healing surgical wound
A bone fracture that has not satisfactorily healed

Main outcome variable

Response rate is defined as the percentage of patients with CR, PR or SD 6 months after start of antiangiogenic treatment, or persistence of NED after complete resection

Additional outcome variables

Overall survival rate: The percentage of patients in the study who are alive at 6, 12, 24, and 36 months after start of treatment with this antiangiogenic multidrug regimen.
Progression free survival rate: The percentage of patients in the study who are alive without progressive disease at 6, 12, 24, and 36 months after start of treatment with this antiangiogenic multidrug regimen.
Toxicity: To evaluate and document toxicities from chronic administration of these drugs at the doses prescribed in this protocol in patients with recurrent or progressive medulloblastoma. These will be descriptive in nature.
Quality of life: QoL will be evaluated by the KINDL®-questionnaire in patients capable of participating.
Performance status: Will be evaluated by the Karnofsky/Lansky scale.
Prognostic factors: To evaluate the influence of tumor biology (histology, subgroups (if available), metastatic stage, age at first diagnosis [<3 years, >3 years]), age at start of antiangiogenic therapy, sex, duration of remission prior to antiangiogenic therapy, number of recurrences.
Tumor/angiogenic markers: To evaluate serum and CSF markers for in-vitro correlative studies of tumor response.

Expected risk/inconvenience

All patients enrolled in this study will receive treatment for therapeutic indications. Relapse of these aggressive brain cancers is a severe and life-threatening disease. Death from tumor progression is typically the natural course of disease in relapsed medulloblastoma and will not be considered a side effect of this study.

Risk/benefit assessment

The prognosis of recurrent or progressive medulloblastoma, ependymoma and ATRT is bleak despite intensive therapeutic approaches. Antiangiogenic therapy inhibits vascular formation, thereby preventing tumor progression indirectly. The major risks of antiangiogenic therapy used in this protocol such as infection are comparable to conventional chemotherapy. In contrast to conventional chemotherapy, antiangiogenic therapy is administered orally and is performed in an outpatient setting.

In summary, the potential benefit of the study clearly outweighs the assumed risks.

2 Table of Contents

Important Contact Information 2
Austria 2
International Study managers 3
Data Monitoring Committee 3
Sponsor 4
Monitor 4
SAE Reporting (FAX) 4
Signatures 4

1 Study synopsis 5

2 Table of Contents 7

3 Notes concerning this protocol 13

4 Glossary 14

5 Scientific / Medical section 16
5.1 Background 16
5.1.1 Introduction 16
5.1.2 Bevacizumab 17
5.1.3 Thalidomide 18
5.1.4 Celecoxib 18
5.1.5 Fenofibrate 19
5.1.6 Etoposide 21
5.1.7 Cyclophosphamide 21
5.1.8 Rationale for combining different oral drugs 21
5.1.9 Intraventricular chemotherapy 21
5.1.9.1 Aqueous cytarabine/ Liposomal cytarabine 22
5.1.9.2 Etoposide 23
5.2 Rationale for the study 23
5.3 Previous experience with this regimen 23
5.4 Aim of the study 24
5.4.1 Objectives 24
5.4.1.1 Primary objectives 24
5.4.1.2 Secondary objectives 24
5.5 Study design 25
5.6 Study population 25
5.6.1 Number of subjects 25
5.6.2 Eligibility Criteria 25
5.6.2.1 Inclusion criteria for patients 25
5.6.2.2 Exclusion criteria for patients 25

6 Description of Treatment Plan 27
6.1 Timepoints 27
6.1.1 Pre-treatment evaluation (T_{-1}) 28
6.1.2 Start of antiangiogenic therapy (T_1) 28
6.1.2.1 Patients not undergoing a resection at the time of recurrence or progression 28
6.1.2.2 Patients undergoing a resection at the time of recurrence or progression 28
6.1.3 Response evaluation week 24 – 28 (T_{24-28}) 28
6.2 On-Study Evaluation 29
6.2.1 Medical history, routine physical and neurologic examination 29
6.2.2 Routine laboratory investigations 29

6.2.3 CSF evaluation......29
6.2.3.1 Cytological Response Criteria......29
6.2.4 Routine neuroradiological investigations......30
6.2.4.1 Time points......30
6.2.4.2 Neuroradiological guidelines......30
6.2.4.3 Radiological criteria for response......31
6.2.5 Determination of tumor/angiogenic markers (optional)......31
6.2.6 Common Terminology Criteria for Adverse Events (CTCAE Version 4.0)......32
6.2.7 Quality of Life......32
6.2.8 Performance status......32
6.2.8.1 Karnofsky performance status......32
6.2.8.2 Lansky performance status for patients less than 12 years of age......32
6.2.9 Nerve conduction velocity......33
6.2.10 Bone age evaluation by X-ray of the left hand (including the wrist)......33
6.3 Post Treatment Follow-up......33
6.4 Drug Administration and Dosing......33
6.4.1 Bevacizumab......33
6.4.1.1 Infusion related reaction/infusional site extravasation management guidelines 33
6.4.2 Thalidomide......35
6.4.3 Celecoxib......35
6.4.4 Fenofibrate......35
6.4.5 Etoposide......35
6.4.6 Cyclophosphamide......35
6.4.7 Intraventricular etoposide......36
6.4.8 Intraventricular cytarabine......36
6.5 Dose modifications......36
6.5.1 Bevacizumab......36
6.5.1.1 Treatment delays......36
6.5.1.2 Bevacizumab discontinuation......37
6.5.1.3 Toxicity Management Guidelines......37
6.5.2 Thalidomide......38
6.5.3 Celecoxib......38
6.5.4 Fenofibrate......38
6.5.5 Initiation of etoposide/cyclophosphamide......39
6.5.5.1 Etoposide......39
6.5.5.2 Cyclophosphamide......39
6.5.6 Dose modifications with respect to ANC (Absolute neutrophil count)......39
6.5.7 Dose modifications for elevated transaminases......39
6.5.8 Intraventricular chemotherapy......39
6.6 Supportive Care Guidelines......39
6.6.1 Pneumocystis prophylaxis......40
6.6.2 Constipation......40
6.6.3 Diarrhea......40
6.6.4 Antiemetic......40
6.6.5 Proton pump inhibitor......40
6.6.6 Growth factors......40
6.7 Patient identification......40

7 Analysis section......41
7.1. Formulation of hypotheses......41
7.1.1 Main question......41
7.1.2 Additional questions......41
7.2 Statistical evaluation......41
7.2.1 Main hypothesis......41

7.2.2 Additional hypothesis 43
7.3 Interruption due to toxicities 43
7.4 Interim analyses and end analysis 44
7.5 Sample size 44
7.6 Relevant protocol deviations 45

8 Study drugs 46
8.1 Bevacizumab 46
8.1.1 Description 46
8.1.2 Classification 46
8.1.3 Molecular Weight 46
8.1.4 Mode of Action 46
8.1.5 Availability and drug storage conditions 46
8.1.6 Stability 46
8.1.7 Preparation 46
8.1.8 Route of Administration 47
8.1.9 Administration 47
8.1.10 Premedication 47
8.1.11 Application 47
8.1.12 Vital Signs 47
8.1.13 Infusion reactions 47
8.1.14 Frequencies of Toxicities 47
8.1.14.1 Additional adverse events, regardless of causality 49
8.1.14.2 CNS hemorrhage (punctuate lesions) 49
8.1.15 Method of Procurement 49
8.2 Thalidomide 49
8.2.1 Description 49
8.2.2 Formulation 50
8.2.3 Reconstitution 50
8.2.4 Application 50
8.2.5 Pharmacology 50
8.2.6 Toxicity 50
8.2.6.1 Neurologic 51
8.2.6.2 Teratogenicity 51
8.2.6.3 Miscellaneous 51
8.2.7 Frequencies of Toxicities 53
8.2.7.1 Additional adverse events, regardless of causality 54
8.2.8 Method of Procurement 54
8.3 Celecoxib 55
8.3.1 Description 55
8.3.2 Formulation 55
8.3.3 Reconstitution 55
8.3.4 Application 55
8.3.5 Pharmacology 55
8.3.6 Distribution 55
8.3.7 Metabolism 55
8.3.8 Excretion 55
8.3.9 Dosing in hepatic disease 56
8.3.10 Renal Insufficiency 56
8.3.11 Drug Interactions 56
8.3.12 Toxicity 56
8.3.12.1 Gastrointestinal 57
8.3.12.2 Cardiovascular 57
8.3.12.3 General 57

8.3.12.4 Resistance mechanism disorders....57
8.3.12.5 Central, peripheral nervous system....57
8.3.12.6 Reproductive....57
8.3.12.7 Metabolic and nutritional....57
8.3.12.8 Musculoskeletal....57
8.3.12.9 Blood....57
8.3.12.10 Respiratory....57
8.3.12.11 Skin....58
8.3.12.12 Application site disorders....58
8.3.12.13 Genitourinary....58
8.3.13 Frequencies of Toxicities....58
8.3.13.1 Additional adverse events, regardless of causality....58
8.3.14 Method of Procurement....59
8.4 Fenofibrate....59
8.4.1 Ingredients....59
8.4.2 Formulation....59
8.4.3 Reconstitution....59
8.4.4 Application....59
8.4.5 Pharmacology....60
8.4.5.1 Cholelithiasis....60
8.4.5.2 Concomitant Oral Anticoagulants....60
8.4.5.3 Concomitant HMG-CoA Reductase Inhibitors....60
8.4.5.4 Pancreatitis....60
8.4.5.5 Hypersensitivity Reactions....61
8.4.5.6 Hematologic Changes....61
8.4.5.7 Skeletal muscle....61
8.4.6 Drug Interactions....61
8.4.6.1 Oral Anticoagulants....61
8.4.7 Toxicity....61
8.4.7.1 Clinical....61
8.4.7.2 Body as a whole....61
8.4.7.3 Cardiovascular....61
8.4.7.4 Digestive....62
8.4.7.5 Endocrine....62
8.4.7.6 Hemic and lymphatic....62
8.4.7.7 Metabolic and nutritional disorders....62
8.4.7.8 Musculoskeletal....62
8.4.7.9 Nervous system....62
8.4.7.10 Respiratory....62
8.4.7.11 Skin and appendages....62
8.4.7.12 Special Senses....62
8.4.7.13 Urogenital System....62
8.4.8 Frequencies of Toxicities....62
8.4.8.1 Additional adverse events, regardless of causality....63
8.4.9 Method of Procurement....64
8.5 Etoposide....64
8.5.1 Description....64
8.5.2 Formulation....64
8.5.3 Reconstitution....64
8.5.4 Application....64
8.5.5 Pharmacology....65
8.5.6 Toxicity....66
8.5.6.1 Hematologic Toxicity....66
8.5.6.2 Gastrointestinal....66

8.5.6.3 Hypotension.....66
8.5.6.4 Allergic Reactions.....66
8.5.6.5 Alopecia.....66
8.5.7 Frequencies of Toxicities.....67
8.5.7.1 Additional adverse events, regardless of causality.....67
8.5.8 Method of Procurement.....68
8.6 Cyclophosphamide.....68
8.6.1 Description.....68
8.6.2 Formulation.....68
8.6.3 Reconstitution.....68
8.6.4 Application.....68
8.6.5 Pharmacology.....68
8.6.6 Toxicity.....69
8.6.6.1 Carcinogenesis, Mutagenesis, Impairment of Fertility.....69
8.6.6.2 Urinary System.....69
8.6.6.3 Cardiac Toxicity.....70
8.6.6.4 Infections.....70
8.6.6.5 Other.....70
8.6.7 Frequencies of Toxicities.....70
8.6.7.1 Additional adverse events, regardless of causality.....71
8.6.8 Method of Procurement.....71
8.7 Intraventricular cytarabine.....71
8.7.1 Description.....71
8.7.2 Formulation.....71
8.7.3 Application.....72
8.7.4 Pharmacology.....72
8.7.5 Frequencies of Toxicities.....72
8.7.6 Method of Procurement.....73
8.8 Intraventricular etoposide.....73
8.8.1 Description.....73
8.8.2 Formulation.....73
8.8.3 Application.....73
8.8.4 Pharmacology.....73
8.8.5 Frequencies of Toxicities.....73
8.8.6 Method of Procurement.....73

9 Ethical section.....75
9.1 Toxicity, inconveniences and risks for subjects.....75
9.2 Toxicity / Safety precautions.....75
9.3 Adverse Events (AE).....75
9.3.1 Causal Relationship of Adverse Events.....75
9.3.2 Grading of Adverse Events.....76
9.3.3 Intensity of Adverse Events.....76
9.3.4 Outcome of Adverse Events.....77
9.3.5 Reporting of Adverse Events.....77
9.4 Serious Adverse Events (SAEs).....77
9.4.1 Reporting of Serious Adverse Events (SAEs).....77
9.5 Unexpected Outcomes.....78
9.6 Contact persons for emergencies.....78
9.7 Risk/benefit assessment.....78
9.8 Informed consent.....78
9.9 Ethical and legal aspects.....78
9.10 Withdrawal criteria.....79
9.11 Premature termination of the trial (stopping rules).....79

10 Administrative section 80
10.1 Documentation 80
10.2 Quality control and quality assurance 80
10.3 Publication of study results 80
10.4 Protocol amendment 80
10.5 Finance and insurance 80
10.6 Error correction 81
10.7 Storage of study documents 81

11 Study schedule for patients 82

12 References 87

13 MEMMAT Appendix 95
A - Graphical Overview 95
B - Performance status 95
C - Tanner stages 95
D - MRI protocol 95
E - Conversion of RCF (g) to RPM 95
F - Bevacizumab Treatment Management for Proteinuria 95
G - Serious Adverse Event (SAE) Report Form 95
H - Pregnancy Notification Form 95
I - Adverse Event Report Form 95
J – End of Therapy Report Form 95
K - Case Report Forms 95
L - Common Terminology Criteria for Adverse Events (CTCAE) 95

3 Notes concerning this protocol

All therapeutic regimens described in this protocol have been checked to ensure correctness. Despite this, errors cannot be ruled out completely. Clinical staff are therefore instructed to use appropriate clinical judgement. Any variance in the protocol should be immediately reported to the Principle Investigators.

Since November 2016 the sole manufacturer for Depocyte, Pacira Pharmaceuticals, is unable to deliver new supplies of Depocyte as planned due to an issue that has been identified with the manufacturing process. In a letter dated August 2017, Pacira Pharmaceuticals disclosed that they will discontinue all future manufacturing and supply of Depocyte. Therefore the protocol has to be amended to include an alternative to intraventricular therapy with liposomal cytarabine. After review from the protocol committee and investigators, and with input from leukemia experts, liposomal cytarabine will be substituted with the parent drug aqueous cytarabine twice a week for two weeks out of every four weeks instead of the one liposomal cytarabine dose every four weeks.

The identification of molecular subgroups of medulloblastoma is gaining prognostic importance. Medulloblastoma molecular subgroup analysis has been added to the protocol and is to be performed whenever possible as this may identify certain subgroups that are either more or less likely to respond to metronomic therapy. Therefore we strongly recommend the communication of all available molecular tumor data (for example molecular group, subgroup and changes in *MYC*, *MYCN*, *TP53*).

4 Glossary

β-HCG	β-human choriogonadotropin
ACE	Angiotensin-converting enzyme
Acetyl-CoA	Acetyl-Coenzyme A
AE	Adverse event
ALT	Alanine aminotransferase
ANC	Absolute neutrophil count
AST	aspartate aminotransferase
ATP	Adenosine triphosphate
ATRT	Atypical teratoid rhabdoid tumor
AUC	Area under the curve
BID	Bis in die ("twice daily")
BUN	Blood urea nitrogen
CPK	Creatine phosphokinase
CNS	Central nervous system
COX	Cyclooxygenase
CR	Complete response
CRP	C-reactive protein
CSF	Cerebrospinal fluid
CSO	Clinical Sample Operations
CTCAE	Common Terminology Criteria for Adverse Events
CVA	Cerebrovascular accident
DNA	Deoxyribonucleic acid
e.g.	Exempli gratia ("example given")
ECOG	Eastern Cooperative Oncology Group
eCRF	Electronic Case Report Form
EFS	Event free survival
EOT	End of treatment
EPC	Endothelial progenitor cell
FSH	Follicle-stimulating hormone
fT4	Free thyroxine
GM-CSF	Granulocyte macrophage colony-stimulating factor
GGT	Gamma-glutamyl transpeptidase
GI	Gastrointestinal
GLUT4	Glucose transporter 4
HDCT	High-dose chemotherapy
HMG-CoA	3-hydroxy-3-methylglutaryl-coenzyme A
IB	Investigator's brochure
IGF-1	Insulin-like growth factor 1
IT	Intrathecal
IV	Intravenous
KINDL	KINDer Lebensqualitätsfragebogen
LD_{50}	Median lethal dose
LH	Luteinizing hormone
MCH	Mean corpuscular haemoglobine
MCHC	Mean corpuscular haemoglobine concentration
MCV	Mean corpuscular volume
MI	Myocardial infarction
MRI	Magnetic resonance imaging
NADH	Reduced nicotinamide adenine dinucleotide
NED	No evidence of disease
NF-κB	Nuclear factor kappa-light-chain-enhancer of activated B cells
NOS	Not otherwise specified

NSAIDs	Non-steroidal anti-inflammatory drugs
OS	Overall survival
PD	Progressive disease
PEI	Ifosfamide-cisplatin-etoposide
PFK	Phosphofructokinase
PFS	Progression free survival
PG	Prostaglandin
PK	Pharmacokinetics
PLTs	Platelets
PNET	Primitive neuroectodermal tumor
po	per os
PPAR	Peroxisome proliferator-activated receptor
PR	Partial response
qam	Quaque ante meridiem ("every morning")
qhs	Quaque hora somni ("every bedtime")
QoL	Quality of life
RBC	Red blood count
SAE	Serious adverse event
SD	Stable disease
SIADH	Syndrome of inadequate secretion of ADH
S.T.E.P.S.®	System for Thalidomide Education and Prescribing Safety
SUSAR	Suspected unexpected serious adverse reaction
T4	Thyroxine
TIA	Transient ischaemic attack
TNF-α	Tumor necrosis factor alpha
TSH	Thyroid-stimulating hormone
ULN	Upper Limit of Normal
VEGF	Vascular endothelial growth factor
WBC	White blood count

5 Scientific / Medical section

5.1 Background

5.1.1 Introduction

Tumors of the central nervous system constitute the largest group of solid neoplasms in children and are second only to leukemia in their overall frequency. Each year, brain tumors develop in approximately 3.5 to 4 children per 100.000. Medulloblastoma is one of the most common malignant brain tumor in children. It constitutes approximately 20% of all central nervous system tumors in children (Kaatsch, 2001; Rosemberg, 2004). Up to 40% of children with medulloblastoma will have disseminated disease at the time of diagnosis, and at the time of recurrence, more than two thirds will have leptomeningeal disease (Packer, 1999; Packer, 2008). The outcome rates of medulloblastoma have significantly improved since the 1980s, mainly through the addition of chemotherapy to radiotherapy and improved staging of disease at diagnosis (Tait, 1990; Evans, 1990; Packer, 2006; von Hoff, 2009).
In the multicenter series HIT-91 (Germany, Austria and Switzerland), fully assessable medulloblastoma patients with clinical low risk factors (M0) achieved excellent long-term survival rates (10-year OS, 91%), when treated with immediate postoperative radiotherapy followed by maintenance chemotherapy. In contrast, survival rates of patients with intracranial (M2) and spinal leptomeningeal spread (M3) were lower with a 10-year OS of 42-45% (von Hoff, 2009). Other brain tumors such as ependymoma and atypical teratoid rhabdoid tumor (ATRT) remain challenges in pediatric oncology as well. Ependymoma represents the second most frequent brain cancer in children occurring in the posterior fossa primarily. Ependymoma have a 59.7% event free survival at diagnosis but challenging to treat at relapse (Jünger, 2019). Currently, the prognosis for ATRT at initial diagnosis is an event free survival of 29% overall while those with single lesion approaches 50% and at relapse the outcome is very poor (Fischer-Valuck, 2017).

Chemotherapy has proven to be efficacious in achieving clinical remissions in newly diagnosed patients. At the time of recurrence, conventional dose chemotherapies typically lead to the selection of tumors that are insensitive to further treatment, possibly due to emergence of drug-resistant clones. Patients with relapsed embryonal brain tumors, whether treated with conventional chemotherapy, high-dose chemotherapy with stem cell rescue, irradiation or combinations of these modalities have a very poor prognosis with a median survival of less than one year in most previous studies (Torres, 1994; Bouffet, 1998; Shih, 2008; Pizer, 2011, Sabel, 2016). Nonetheless, most children who are candidates for further palliative therapy are offered various types of chemotherapy, all of which are associated with limited efficacy and, at times, substantial toxic effects.

Alkylator-based high-dose chemotherapy represents the most aggressive chemotherapeutic approach in children with recurrent medulloblastoma. Although different studies have shown limited efficacy of high-dose chemotherapy in recurrent medulloblastoma, only a small subset of children benefit, mostly those who have not been previously irradiated (Shih, 2008; Gururangan, 2008; Dunkel, 2010).

Angiogenesis, the formation of new blood vessels, is an important component of normal physiological processes such as wound healing. Moreover, tumor growth and metastases are dependent on the formation of new blood vessels (Folkman, 1971; Folkman, 1992). Angiogenesis involves the degradation of extracellular matrix combined with migration and sprouting of endothelial cells.
Antiangiogenic therapy inhibits vascular formation, thereby inhibiting tumor progression indirectly. Conventional administration of chemotherapeutic agents typically requires a treatment-free period for the recovery of normal cells. During this recovery period, mobilization of endothelial progenitor cells (EPCs) occurs, and the few endothelial cells that

had been killed are replaced by aggressive endothelial proliferation and therefore resume tumor growth during this treatment-free period (Browder, 2000; Klement 2000). In contrast to conventional chemotherapy, low-dose metronomic scheduling of cytotoxic substances can reduce the release of endothelial progenitor cells (EPC) into the circulation (Stoelting, 2008). Antiangiogenic therapy also has the ability to damage or kill the genetically stable, host endothelial cell, with subsequent induction of tumor dormancy or cytostasis.

Antiangiogenic therapy has emerged as a new treatment option in solid malignancies. The frequent, low dose metronomic schedule targets endothelial cells and minimizes toxicity (André, 2009; Zacharoulis, 2010, Pasquier, 2010).

Previous reports have shown that some patients who do not achieve long-term remission with established high-intensity regimens, may benefit from treatment protocols with prolonged, low-intensity chemotherapy (Chamberlain, 1995; Chamberlain, 1997; Asou, 2000; Glode, 2003; Kieran, 2005; Stockklausner, 2006; Buckstein, 2006; Sterba, 2006; Krzyzanowska, 2007; André, 2008; Dellapasqua, 2008; Garcia, 2008; Sung, 2008; Packer, 2009; Sterba, 2010).

Since various trials have demonstrated that antiangiogenic drugs are generally insufficient as single agents, a combination of different agents is considered to be more promising (Klement, 2002; Bello, 2001; Bergers, 2002; Panigrahy, 2010).

In this study we will evaluate the use of intravenous bevacizumab every two weeks in combination with continuous oral thalidomide, celecoxib, fenofibrate and low-dose oral chemotherapy with etoposide and cyclophosphamide, augmented with intraventricular etoposide, intraventricular aqueous cytarabine or liposomal cytarabine.

5.1.2 Bevacizumab

Bevacizumab is a recombinant humanized monoclonal IgG1 antibody that binds to and inhibits the biologic activity of human vascular endothelial growth factor (VEGF). Bevacizumab binds VEGF and inhibits the interaction of VEGF with its receptors on the surface of endothelial cells, preventing endothelial cell proliferation and new blood vessel formation.

Preclinical studies, as well as early-phase clinical studies have shown that VEGF-targeted therapy can inhibit the mobilization of EPCs and their presumed incorporation into tumor vasculature, while also decreasing perfusion, microvascular density and interstitial fluid pressure (Willett, 2004).

After administration of a single dose of bevacizumab, pharmacokinetic studies demonstrated a half-life of approximately 18 days in adult patients. The predicted time to reach steady state is 100 days on a once every 2 weeks dosing schedule on a mg/kg basis (Gordon, 2001; Hsei, 2001; Hsei, 2002; Lu, 2008). Various studies support the effectiveness of bevacizumab in brain tumors (Vredenburgh, 2007; Sathornsumatee, 2008; Desjardins, 2008; Kreisl, 2009; Poulsen, 2009; Zuniga, 2009; Packer, 2009).

In a pediatric Phase I trial, bevacizumab was administered biweekly at doses up to 15 mg/kg in 18 children with refractory solid tumors. No dose-limiting toxicities were observed, and a maximum tolerated dose was not defined. Drug exposure was proportional to dose in this pediatric study, whereas half-life values varied over a four-fold range with a median value of 12 days. Since drug disposition in pediatric patients seemed to be similar to that observed in adults, common adult bevacizumab dosing schedules are pursued in pediatric patients (Glade Bender, 2008).

We retrospectively analyzed 30 patients with primary CNS tumors who received a total of 478 courses of bevacizumab. Overall, bevacizumab treatment was well tolerated. No bevacizumab related intratumoral hemorrhage occurred in any of our 30 patients. Grade III

hypertension was seen in two patients. One patient developed nephrotic syndrome, requiring cessation of treatment. Grade I and III proteinuria were observed in five and one patient, respectively. New onset lymphopenia occurred in 12/30 and new onset hypothyroidism in 7/30 patients. Impaired wound healing was manageable. No immediate bevacizumab-related cardiotoxicity was observed as evidenced by echocardiography (Reismueller, 2010).

5.1.3 Thalidomide

Thalidomide is a glutamic acid derivate with immunomodulatory and sleep-promoting characteristics, developed in the 1950’s as a sedative. In rodent models the drug seemed to be so non-toxic that a LD_{50} could not be established. Unfortunately, thalidomide turned out to be a potent teratogen in humans (McBride, 1961; Lenz, 1962). In 1994 D’Amato et al. found that orally administered thalidomide is a potent inhibitor of angiogenesis in the rabbit and mouse corneal neovascularization assay (D’Amato, 1994; Kenyon, 1997). This finding suggested that thalidomide’s teratogenicity may be related to an inhibition of blood vessel growth in the developing limb. The developing limb bud is indeed unique in requiring a complex coordination of angiogenesis and vasculogenesis, thus potentially making it an extremely sensitive target for inhibition of neovascularization. As an immunomodulator, thalidomide inhibits the production of TNF-α in lipopolysaccharide-induced human monocyte and mouse macrophages by enhancing degradation of TNF-α mRNA (Sampaio, 1991; Moreira, 1993). Thalidomide blocked activation of NF-kB, a transcription factor positively regulating immune and inflammatory response (Keifer, 2001). Moreover, thalidomide reduced the proliferation of endothelial cells *in vitro* (Moreira, 1999).

Thalidomide has been evaluated as a single agent or in combination with various anticancer drugs in the treatment of adult and childhood malignancies. In adult glioblastoma, thalidomide in combination with temozolomide appeared to be reasonably safe, but with little improvement to temozolomide alone (Groves, 2007). Similarly, the combination of irinotecan and thalidomide had limited activity against adult glioblastoma (Fadul, 2008). As a single agent, thalidomide was modestly tolerated up to a daily dose of 300 mg in patients with hepatocellular carcinoma. Patients with a tumor smaller than 5 cm in size showed a better response to this treatment, suggesting that thalidomide preferentially inhibits the genesis of new blood vessels rather than interfering with mature vessel stability (Pinter, 2008). Thalidomide showed antiangiogenic effects on occult hepatic metastases suggesting that thalidomide has utility as an antiangiogenesis agent, especially in the early stage of tumor development (Liu, 2009).

A series of multi-agent pediatric phase I and II studies containing thalidomide have been completed. In a patient with multiple recurrent metastatic neuroblastoma, thalidomide as a single agent led to shrinkage of an abdominal mass and pulmonary metastases (Gesundheit, 2007). In children with brainstem tumors, the combination of thalidomide and carboplatin-vincristine-fluvastatin significantly increased survival (Lopez-Aguilar, 2008). The combination of thalidomide with celecoxib, etoposide and cyclophosphamide was very well tolerated and was able to produce prolonged or persistent disease-free status in patients with recurrent solid tumors (Kieran, 2005, Robison, 2013).

5.1.4 Celecoxib

Celecoxib is a non-steroidal anti-inflammatory drug (NSAID). Celecoxib is a highly selective cyclooxygenase (COX) 2 inhibitor and primarily inhibits this isoform of cyclooxygenase, whereas traditional NSAIDs inhibit both COX-1 and COX-2. The COX-enzyme catalyzes the rate-limiting step in arachidonate metabolism, resulting in prostaglandin (PG) production. COX-2 is an inducible enzyme responsible for PG production at sites of inflammation (Smith, 1996).

The role of anti-inflammatory drugs in cancer is now being investigated, especially for the prevention of colon and breast cancer. In rodent models of familial adenomatous polyposis, a genetic disorder leading to colon cancer, blockade of COX-2 by gene deletion or pharmacologic inhibition of enzyme activity suppresses intestinal polyp formation (Jacoby,

2000). Regular ingestion of NSAIDs or aspirin reduces the relative risk of death from colon cancer by 40-50%, suggesting that inhibition of COX in humans has a chemopreventive effect (Garcia-Rodriguez, 2001; Harris, 2008). These data provide evidence that COX-2 enzyme activity is important in oncogenesis. Treatment with celecoxib at a dose of 400 mg twice daily was associated with significant regression of colorectal adenomas in patients with familial adenomatous polyposis. Significant regression was not associated with 100 mg twice daily (Steinbach, 2000). Celecoxib taken for two or more years was associated with a 71% reduction in breast cancer risk (Harris, 2006).
COX-2 levels have been shown to be frequently elevated in high-grade gliomas, and selective inhibition of COX-2 leads to reduced growth activity (Joki, 2000). Significant expression of COX-2 has been detected in the neovasculature of human breast adenocarcinomas (Davies, 2003). Celecoxib inhibited the COX-2-derived prostaglandin production in the corneal micropocket and decreased proliferation as well as induced apoptosis of angiogenic cells in rat cornea (Leahy, 2002). The ability of specific COX-2 inhibitors to decrease tumor-angiogenesis and tumor growth has also been demonstrated in a number of *in vitro* and *in vivo* model systems (Tsujii, 1997; Tsujii, 1998; Yoshida, 2003).

The safety and tolerability of celecoxib has been demonstrated in a number of clinical trials in adults (Bingham, 2007; Alvarez-Soria 2008; Nissen, 2016). For example, the gastrointestinal (GI) tolerability of celecoxib at multiple doses was no more GI toxic than placebo, and much less than that observed with non-selective NSAIDs (Rostom, 2007). Celecoxib was well tolerated in children with juvenile rheumatoid arthritis with 3 mg/kg and 6 mg/kg twice daily (Foeldvari, 2009). Clinical phase I and II trials combining celecoxib with low-dose cyclophosphamide showed that this combination was generally well tolerated in adult patients (Buckstein, 2006; Krzyzanowska, 2007). Similarly, trials in pediatric patients with relapsed cancer showed that celecoxib was not associated with major toxicities (Kieran, 2005; Stempak, 2006; André, 2008; Choi, 2008; Sterba, 2010).

5.1.5 Fenofibrate

Fenofibrate is a prodrug analogue of clofibrate, a class of drugs used to reduce serum triglycerides and cholesterol levels by activating the nuclear peroxisome proliferator-activated receptor (PPAR). PPARs are members of the steroid receptor superfamily and act as ligand-activated transcriptional factors. Activation of PPARs by ligands results in the regulation of transcription by heterodimerization with the retinoic acid-X receptor. The resulting heterodimer associates with a peroxisome proliferators-response element found in the enhancer region of target genes, a relatively homogenous group of genes that participate in aspects of lipid catabolism such as fatty acid uptake through membranes, fatty acid binding in cells, fatty acid oxidation (in microsomes, peroxisomes and mitochondria), and lipoprotein assembly and transport (Kliewer, 1992; Kersten, 2000). Activators of the subtype PPARα include high-affinity synthetic ligands, such as the lipid-lowering fibrates (Bishop-Bailey, 2000; Neve, 2000). Fibrates are a synthetic class of drugs that act as agonists to the PPARα pathway and have been used for 3 decades to help control triglyceride and cholesterol metabolism due to induction of fatty acid oxidation.
The interrelationships among PPARs, mitochondria and cancer are extremely complex and seem to act on different target areas of the cell, which are currently under investigation by many groups. An important factor linking cancer to PPARs is represented by their synthetic ligands, which are characterized by receptor- and extra-receptor activities, such as functions in lipid and glucose metabolism, inflammation, and antiangiogenesis (Kersten, 2000; Lee, 2003, Scatena, 2008; Pozzi, 2008).
Recent evidence indicates an association of peroxisome proliferators with inhibition of cancers. PPARα ligands suppress the growth of several cancer cell lines *in vitro*, including colon, breast, endometrium and skin (Thuillier, 2000; Tanaka, 2001; Maggiora, 2004; Saidi, 2006). A previously published report described the addition of lipid-lowering agents to retinoic acid during therapy of cutaneous T cell lymphoma in humans in order to treat

hyperlipidemia associated with this therapy. While the response rate for patients on retinoids alone was approximately 48%, those receiving fenofibrate to control their hyperlipidemia demonstrated response rates of 90% (Talpur, 2002).

Antiangiogenesis is one of the antitumorigenic properties of PPARα ligands. PPARα ligands have been shown to inhibit VEGF-mediated migration of endothelial cells and decrease microvessel density within intimal tissues in animals (Goetze, 2002; Libby, 2001). Fenofibrate suppressed endothelial cell proliferation and VEGF expression in various cell lines, and inhibited corneal neovascularization (Panigrahy, 2008). In patients with hyperlipidemia, successful lipid-lowering treatment was associated with a reduction in plasma VEGF (Blann, 2001).

Another important part of PPARα ligands is their role in energy metabolism of tumor cells. A common property of cancer cells is an altered glucose metabolism, known as the "Warburg effect". In the presence of oxygen, most differentiated cells primarily metabolize glucose to carbon dioxide by oxidation of glycolytic pyruvate in the Krebs cycle to maximize ATP production, with minimal production of lactate. In contrast to differentiated cells, most cancer cells produce large amounts of lactate regardless of the availability of oxygen. This metabolism is often referred to as "aerobic glycolysis". Warburg originally hypothesized that cancer cells develop a defect in mitochondria that leads to impaired aerobic respiration and a subsequent reliance on glycolytic metabolism (Warburg 1924; Warburg 1956). Indeed, mitochondrial DNA shows high rates of mutations in cancer cells, leading to malfunction in respiration and oxidative phosphorylation (Carew, 2002). On the other hand there is evidence that mitochondrial function is intact in many cancer cells (Moreno-Sanchez, 2007). A possible explanation for the switch to "aerobic glycolysis" is that proliferating cells have important metabolic requirements beyond ATP production. To produce two viable daughter cells at mitosis, a proliferating cell must replicate all of its cellular contents, requiring nucleotides, amino acids and lipids. Converting all of the glucose to CO_2 via oxidative phosphorylation in the mitochondria to maximize ATP production does not meet the demand of a proliferating cell. Some glucose must be diverted to macromolecular precursors such as acetyl-CoA for fatty acids, glycolytic intermediates for nonessential amino acids, and ribose for nucleotides. This may explain at least in part the selective advantage of the Warburg effect (for review, see Vander Heiden, 2009). PPARα, which is a transcriptional activator of fatty-acid β-oxidation machinery, can switch energy metabolism towards fatty acid degradation, and decrease glucose uptake by repressing the glucose transporter GLUT4 and the glycolytic enzyme phosphofructokinase (PFK) (Finck, 2002a; Finck, 2002b; Ahmed, 2007). GLUT4-mediated transport represents a major mechanism by which the heart increases glucose uptake during ischemia. This mechanism is critical for myocardial protection during ischemia and for subsequent recovery of the heart after ischemic insults (Tian, 2001). Comparable to an ischemic heart, cancer cells are often in a hypoxic tissue environment, and the increase in glycolysis may be viewed as cellular adaption to hypoxia (Gatenby, 2004), thereby, maintaining a high level of glycolytic activity essential for cancer cells to survive. Inhibition of glycolysis under hypoxic condition may severely abolish energy metabolism in cancer cells and may subsequently kill the malignant cells.

In the last years, a number of extra-receptor functions of fibrates have been reported. Fibrates can disrupt the mitochondrial electron respiratory chain at the level of complex I, the NADH dehydrogenase, or NADH-ubiquinone oxidoreductase. Thus, cells are pushed towards a series of complex compensatory adaptations (Scatena, 2003). Additionally, preincubation of medulloblastoma cells with fenofibrate partially inhibited IGF-1-mediated phosphorylation (Urbanska, 2008).

Fenofibrate has been prescribed millions of times for patients with hypercholesterolemia and has demonstrated excellent tolerability (Keech, 2005; Mombelli, 2009). Fenofibrate has been used in children for different indications and was also well tolerated (Choi, 2006; Cree, 2007).

5.1.6 Etoposide

Etoposide phosphate is an inhibitor of the enzyme topoisomerase II and has a major role in the management of many childhood cancers.

The use of chronic low-dose etoposide has been used predominantly in the palliative setting (Davidson, 1993; Needle, 1997). Long-term therapy with oral etoposide is one chemotherapeutic approach in children with recurrent brain tumors, although it might result in an increased rate of secondary leukaemia (Le Deley, 2005). Oral etoposide is attractive for a number of reasons, including the ease of administration, modest toxicity, and antiangiogenic mechanism of action (Davidson, 1993; Chamberlain, 1995; Ashley, 1996, Needle, 1997; Chamberlain 1997; Panigrahy 2010).

Prolonged oral etoposide has shown activity against several tumors initially refractory to short-term intravenous infusions of this drug. A number of reports have been published documenting clinical responses in patients treated with daily dosing cycles. Oral administration of etoposide demonstrated tolerability in both pediatric and adult patients (Davidson, 1993; Chamberlain, 1995; Needle, 1997; Chamberlain, 1997; Kieran, 2005; Korones, 2006; Stockklausner, 2006; André, 2008; Choi, 2008; Sterba, 2010, Robison, 2013).

5.1.7 Cyclophosphamide

Cyclophosphamide is a nitrogen mustard alkylating agent. Cyclophosphamide at the maximally tolerated dose is a component of a large number of pediatric and adult treatment regimens.

There are reported cases of dramatic palliation using oral low-dose cyclophosphamide (Asou, 2000; Sung, 2009). In a phase-II clinical trial, the combination of low-dose metronomic cyclophosphamide and bevacizumab had significant activity in recurrent ovarian cancer (Garcia, 2008). In another study, metronomic cyclophosphamide and capecitabine combined with bevacizumab provided long-term disease control in a high proportion of patients with advanced breast cancer, without significant toxicity despite prolonged use (Dellapasqua, 2008). Low-dose cyclophosphamide in combination with dexamethasone demonstrated clinical activity against prostate carcinoma (Glode, 2003). Low-dose cyclophosphamide given continuously in combination with celecoxib was shown to be effective in patients with relapsed or refractory aggressive non-Hodgkin’s lymphoma (Buckstein, 2006).

In pediatric patients, a metronomic regimen containing cyclophosphamide was not associated with major toxicities (Kieran, 2005; Stempak, 2006; André, 2008; Choi, 2008, Robison, 2013).

5.1.8 Rationale for combining different oral drugs

Kieran et al showed that antiangiogenic metronomic chemotherapy combining four oral drugs (thalidomide, celecoxib, etoposide, cyclophosphamide) showed prolonged or persistent disease-free status in a group of heavily pretreated pediatric patients with recurrent or progressive cancer (Kieran, 2005). More recently, the combination of low-dose oral etoposide with the cyclooxygenase-2 (COX2) inhibitor celecoxib and the peroxisome-proliferator activated receptor (PPAR) ligand rosiglitazone has been shown to have synergistic anti-tumor activity and enhanced the anti-tumor activity of low-dose oral etoposide by 69% (Panigrahy, 2010).

In the current protocol, the oral drug regime will include the established oral five-drug combination thalidomide, celecoxib, etoposide, cyclophosphamide, augmented with the oral PPAR-ligand fenofibrate (Robison, 2013). A 101 patient trial of the combination of thalidomide, celecoxib, fenofibrate and alternating oral VP-16 and cyclophosphamide has recently been completed. The 5-drug combination again showed excellent tolerability (Robison, 2013).

5.1.9 Intraventricular chemotherapy

Treatment of recurrent medulloblastoma is complicated by the high rate of leptomeningeal dissemination. At recurrence, more than two thirds of medulloblastomas show leptomeningeal

dissemination (Packer, 1999). Since antiangiogenic metronomic therapy does not reach the CSF compartment, regional intraventricular chemotherapy is one approach to treat tumor cells floating in the CSF.
Intraventricular chemotherapy via an Ommaya-reservoir with etoposide and liposomal cytarabine has been shown to be well-tolerated in pediatric patients (Peyrl, 2009; Fleischhack, 2001). Ommaya reservoirs have shown to be safe and complications are infrequent providing that all personnel involved in implanting and subsequently accessing the device are specially trained and pay meticulous attention to strict aseptic conditions (Peyrl, 2014).

5.1.9.1 Aqueous cytarabine/ Liposomal cytarabine
For cell-cycle-specific antineoplastic drugs such as cytarabine, duration of exposure of neoplastic cells to cytotoxic drug concentrations is a critical factor influencing therapeutic efficacy. Due to the short half-life of free cytarabine after intrathecal injection of only 3.4 hours, cytotoxic concentrations are maintained for about 24 hours (Zimm, 1984). Liposomal cytarabine (Depocyte®) is a slow-release formulation, in which cytarabine is encapsulated in microscopic particles that average 19 µm in diameter, containing numerous nonconcentric chambers bound by a single bilayer lipid membrane (Kim, 1987). Cytarabine is gradually released from these particles into the CSF, thereby prolonging exposure to the drug and intensifying the effect.
The recommended dose of liposomal cytarabine for adults is a single injection of 50 mg every 2 weeks, leading to a mean elimination half-life of free cytarabine in CSF of 80 h, versus 3.4 h for native cytarabine (Kim, 1993). Children showed a more rapid elimination of liposomal cytarabine compared with adults treated at similar dose levels (Bomgars, 2004). In adult patients with solid tumor neoplastic meningitis, liposomal cytarabine produced a response rate comparable to that of methotrexate and significantly increased the time to neurological progression while offering the benefit of a less demanding dose schedule (Glantz, 1999). In high-risk embryonal tumors of children and young adults, liposomal cytarabine was shown to be an active drug, with demonstrable responses, and was associated with longer median time to progression-free and overall survival (Partap, 2011).
The administration of 25 mg liposomal cytarabine in children less than 3 years of age appeared to be safe and well-tolerated if combined with concomitant dexamethasone. With this dose, the drug exposure in CSF in children below 3 years is comparable to that achieved in adult patients after administration of 50 mg (Peyrl, 2009). Liposomal cytarabine in doses of 35 mg for children aged 3-10 years and 50 mg for older patients showed sufficient drug exposure for at least 1 week and appeared to be well tolerated (Peyrl, 2014).
After discontinuation of the production of liposomal cytarabine in 2017, aqueous cytarabine will be used instead of liposomal cytarabine. Since liposomal cytarabine is a slow release formulation with a long exposure time, aqueous cytarabine will be administered on a twice weekly schedule for two weeks (days 1, 4, 8, and 11) out of every four weeks instead of the one dose liposomal cytarabine every four weeks as published previously (Glantz, 1999) and similar to intensified intrathecal therapy in CNS-positive mature B-Cell Non-Hodgkin's Lymphoma (Attarbaschi, personal communication; Cairo, 2012).

Figure 1: Pharmacokinetics of intraventricular treatment

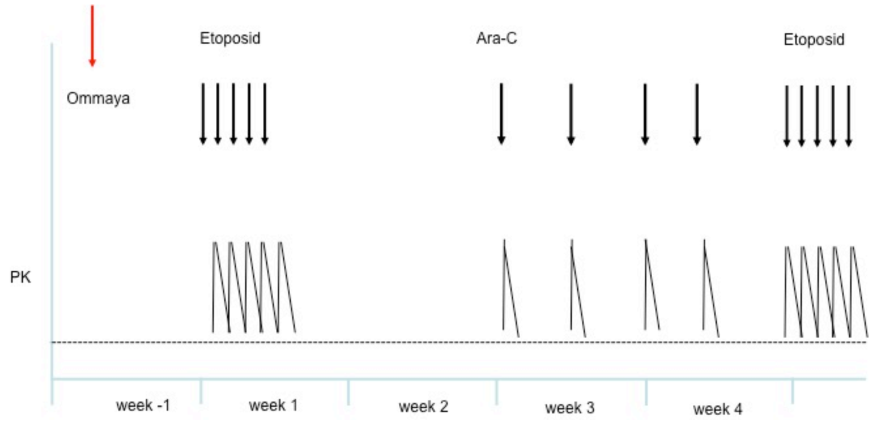

5.1.9.2 Etoposide

Experimental data on medulloblastoma cell lines as well as clinical data on systemic administration of etoposide in patients with neuroectodermal brain tumors reveal a significant cytotoxic activity of etoposide in these tumors (TomLinson, 1991; Dunkel, 1998). Pharmacokinetic data of intraventricular administration of 0.5 mg etoposide for 5 consecutive days showed that no drug accumulation occurred (Fleischhack, 2001). Intraventricularly administered etoposide at a dose of 0.5 mg × 5 days has been shown to be feasible, safe and may produce responses in children with embryonal tumors (Slavc, 2003).

5.2 Rationale for the study

Prognosis of recurrent medulloblastoma, ependymoma and ATRT remain dismal and new treatment strategies are urgently needed. Moreover, children with recurrent medulloblastoma, ependymoma and ATRT are heavily pretreated, thus salvage treatment has to be well tolerated if used in a palliative setting.

In this study we will evaluate the use of intravenous bevacizumab every two weeks in combination with continuous oral thalidomide, celecoxib, fenofibrate and low-dose oral chemotherapy with etoposide and cyclophosphamide.

Since these agents all inhibit angiogenesis via a number of different mechanisms, it is expected that the combination may potentially be more effective than individual agents alone. Therapy is augmented with alternating intraventricular etoposide and liposomal cytarabine or aqueous cytarabine, to treat or prevent leptomeningeal dissemination. Five of the drugs offer the convenience of oral dosing, bevacizumab is administered by infusion every two weeks, and all of the drugs have been well tolerated in adults and children alike.

5.3 Previous experience with this regimen

The agents used in the proposed antiangiogenic therapy are based on a report by Kieran et al. with oral thalidomide, celecoxib and fenofibrate with alternating etoposide and cyclophosphamide every 21 days (Kieran, 2005; Robison, 2013), augmented with intravenous bevacizumab and intraventricular chemotherapy. We have used this regimen in children with recurrent embryonal brain tumors with satisfactory clinical success (Peyrl, 2012; Peyrl, 2014). Therapy consisted of daily oral thalidomide, fenofibrate, celecoxib, and 21-day cycles of low dose oral etoposide alternating with cyclophosphamide augmented with IV bevacizumab and intraventricular therapy (etoposide and liposomal cytarabine; after 2017 aequeous cytarabine).

As of end of 2019, the median OS of 20 patients with medulloblastoma treated according to this approach was 44 months, the 3-year OS 55.0 ±11.1% and 5-year OS 40.0 ±11.0% (Slavc, 2019). The median OS of 12 patients with ATRT was 24 months, the 3-year OS 48.6 ±14.8% and 5-year OS 36.5 ±15.3% (Slavc, 2018). The median OS of 10 patients with ependymoma was 20 months, the 3-year and 5-year OS 33.3 ±17.2% (Slavc, 2018).

Therapy was generally well tolerated and toxicities were manageable (Peyrl, 2012; Slavc, 2018; Slavc, 2019).

5.4 Aim of the study

The aim of the study is to extend therapy options for children with recurrent or progressive medulloblastoma, ependymoma, and ATRT, for which no known curative therapy exists, by prolonging survival while maintaining good quality of life. The current trial has been proposed in three independent strata to determine whether clinical benefit of this combination can be realized.

5.4.1 Objectives

5.4.1.1 Primary objectives

The primary objective is to determine the response rate defined as the percentage of patients with CR, PR, SD or lack of recurrence (see 6.2.4.3 for radiological criteria for response) at 6 months after start of antiangiogenic treatment.

5.4.1.2 Secondary objectives

- To determine the overall survival rate defined as the percentage of patients in the study who are alive at 6, 12, 24, and 36 months after start of treatment with this antiangiogenic multidrug-regimen.
- To determine the progression free survival rate defined as the percentage of patients in the study who are alive without progressive disease at 6, 12, 24, and 36 months after start of treatment with this antiangiogenic multidrug-regimen.
- To evaluate and document toxicities from chronic administration of these drugs at the doses prescribed in this protocol in patients with recurrent or progressive medulloblastoma. These will be descriptive in nature.
- To evaluate quality of life by the KINDL®-questionnaire in patients capable of participating (see 6.2.7).
- To evaluate the performance status at 6 months after start of treatment with this antiangiogenic multidrug-regimen by applying the Karnofsky performance status in children 12 years of age or older and the Lansky play scale ≥50% in infants and children less than 12 years of age (see 6.2.8).
- To evaluate prognostic factors including tumor biology (histology, molecular group, subgroup, changes in *MYC*, *MYCN*, *TP53*, tumor burden/metastatic stage, age at first diagnosis [<3 years, >3 years]), age at start of antiangiogenic therapy, sex, duration of remission prior to antiangiogenic therapy, number of recurrences. These will be descriptive in nature.
- To evaluate tumor/angiogenic markers in serum and CSF.

5.5 Study design

The study will follow an open label, single arm phase II design in three independent strata.

5.6 Study population

Patients who fulfil the eligibility criteria will be evaluated for study participation. All therapeutic decisions (e.g. onset, duration and discontinuation of therapy) need to ensure the safety of the patients at all times.

5.6.1 Number of subjects

In total, 40 patients will be enrolled in the study in Stratum I (medulloblastoma), 30 patients will be enrolled in Stratum II (ependymoma), and 30 patients will be enrolled in Stratum III (ATRT). This number will be sufficient for the purpose of this study using a minimax three-stage design for phase II oncology clinical trials according to Chen and Shan (Chen and Shan, 2008) for statistical analysis (I: alpha 0.05, beta 0.10; II+III: : alpha 0.05, beta 0.20) . We expect to recruit the study patients within a maximum period of ten years.

5.6.2 Eligibility Criteria

Patients with relapsed or progressive medulloblastoma, ependymoma and ATRT, for whom no available curative therapy exists.

5.6.2.1 Inclusion criteria for patients

- Stratum I: Relapsed or progressive medulloblastoma (at least one site of untreated recurrent disease)
- Stratum II: Relapsed or progressive ependymoma (at least one site of untreated recurrent disease)
- Stratum III: Relapsed or progressive ATRT (at least one site of untreated recurrent disease)
- Histological confirmation of medulloblastoma/ependymoma/ATRT at diagnosis or relapse
- Female or male, aged from 0 to <20 years (at time of original diagnosis)
- Participants must have normal organ and bone marrow function (ALT <5x institutional upper limit of normal, creatinine <1.5x institutional upper limit of normal for age, WBC >1000/mm^3, platelets > 20,000/mm^3. Patients with values less than WBC 2000/mm^3 or platelets 50,000/mm^3 will require initiation of treatment with etoposide and cyclophosphamide at a lower starting dose as defined within the protocol.
- Karnofsky performance status ≥50. For infants and children less than 12 years of age, the Lansky play scale ≥50% will be used
- Written informed consent of patients and / or parents

5.6.2.2 Exclusion criteria for patients

- Active infection
- VP-shunt dependency
- Pregnancy or breast feeding
- Conventional chemotherapy, antiangiogenic treatment or complete irradiation of all disease for current relapse (surgery may be performed before antiangiogenic treatment; patients with sites of disease not irradiated are still eligible for the protocol)
- Known hypersensitivity to any of the drugs in the protocol
- Active peptic ulcer
- Any significant cardiovascular disease not controlled by standard therapy e.g. systemic hypertension
- Anticipation of the need for major elective surgery during the course of the study treatment

- Any disease or condition that contraindicates the use of the study medication/treatment or places the patient at an unacceptable risk of experiencing treatment-related complications
- Non-healing surgical wound
- A bone fracture that has not satisfactorily healed

6 Description of Treatment Plan

Prior to inclusion all patients and/or parents will be informed about the objectives of the present study and the risks involved. Upon signing of the informed consent (and assent where appropriate) form and provided that the subjects fulfil the eligibility criteria, patients will be enrolled in the study. If a participant does not receive protocol therapy within 21 days following enrolment, the participants protocol status must be changed.

Bevacizumab is administered every two weeks; thalidomide and fenofibrate are administered daily and celecoxib is administered twice daily. Daily oral etoposide will be alternated with daily oral cyclophosphamide every 21 days.
Intraventricular therapy will be applied with alternating etoposide (day 1-5, one week out of every four weeks) and aqueous cytarabine twice a week for two weeks (days 1, 4, 8 and 11, start +/- 3 days); until 2017 liposomal cytarabine (day 3, +/- 3 days, one week out of every four weeks)

Doses of oral etoposide and cyclophophamide will be modified according to ANC (see 6.5.5 and 6.5.6). Should either etoposide or cyclophosphamide be held, bevacizumab, thalidomide, fenofibrate and celecoxib, as well as intraventricular chemotherapy will be given continuously at the treating physician's discretion. The course is completed after 21 days, even if therapy with either etoposide or cyclophosphamide has been held. *Missed doses are not made up.* Patients who are too young or are incapable of swallowing pills may have medications sprinkled on ice cream, apple sauce, pudding etc., or through a gastric tube. If a pill is vomited and can be identified, then the dose can be repeated. If the pill is not identifiable, then the dose is not repeated. Doses that have been sprinkled will not be made up if vomited, even if pill fragments are visible. All such occurrences will be recorded in the case report form.

Official treatment duration will be one year but should be extended depending on tolerability and response for a second year.
Intervals of intraventricular therapy may be extended after 6 months if no signs of leptomeningeal dissemination are present.
Oral etoposide and cyclophosphamide should be administered for a minimum of 6 months, should continue when tolerated for another 6 months, and may be discontinued at any time after the first 6 months at the investigators discrection for concerns of possible long-term toxicity (i.e., potential risk of secondary leukemia).
If study treatment is missed for an extended period of time due to non-compliance of the subject, the study treatment may be discontinued at the treating physician's discretion.

After the mandatory MRI evaluation for response at 6 months (week 24-28; see 6.1.3), radiotherapy may be performed concomitant with antiangiogenic therapy at the treating physician's discretion.

Recruitment period: A total of 40 patients should be enrolled over a maximum of five years in Stratum I (medulloblastoma), a total of 30 patients should be enrolled over a maximum of five years in Stratum II (ependymoma), and a total of 30 patients should be enrolled over a maximum of five years in Stratum III (ATRT).
Duration of the study: Follow-up from initiation of therapy will continue for at least 3 years. The maximum planned study duration for each stratum is therefore 8 (5+3) years.

6.1 Timepoints

6.1.1 Pre-treatment evaluation (T_{-1})
Prestudy evaluation (T_{-1}, not older than two weeks) includes informed consent, demographic data, routine physical (see 6.2.1) and neurologic examination (see 6.2.1), screening dipstick urinalysis for proteinuria, MR imaging (see 6.2.4), routine laboratory investigations, hormone status, immunology (see 6.2.2), serum, CSF and urine for determination of tumor/angiogenic markers (see 6.2.5), pregnancy test, CTCAE (see 6.2.6), quality of life (see 6.2.7), performance status (see 6.2.8), nerve conduction velocity (see 6.2.10) and X-ray of the left hand (see 6.2.11).

CSF for cytology and malignant cell count from lumbar CSF prior to start of treatment if safe (see 6.2.3).

Serum, CSF and urine should be collected as indicated in 6.2.5 before start of antiangiogenic treatment.

Pregnancy testing must be performed on all pubertal females (breast or pubic hair Tanner stage II or higher) within 24 hours prior to initiation of thalidomide therapy or as per institutional guidelines.

6.1.2 Start of antiangiogenic therapy (T_1)
The start of antiangiogenic therapy is considered when thalidomide, celecoxib and fenofibrate have been initiated.

6.1.2.1 Patients not undergoing a resection at the time of recurrence or progression
Antiangiogenic therapy should start no longer than two weeks after pre-treatment MRI. Medication with etoposide / cyclophospamide and bevacizumab as well as intraventricular treatment with etoposide / aqueous cytarabine should start as soon as appropriate after initiation of oral antiangiogenic therapy.
Bevacizumab should not be initiated within two days of minor surgical procedures (including the placement of a central venous access device) and should not be initiated within 7 days of the placement of an Ommaya-reservoir. Considering impaired wound healing, we recommend non-absorbable sutures for any surgical procedure, and the sutures should be left in place for an extended period.
Intraventricular therapy should not be initiated within five days after placement of the Ommaya reservoir to allow wound-healing.

6.1.2.2 Patients undergoing a resection at the time of recurrence or progression
In case of tumor surgery, a postoperative MRI must be obtained within 72 hours (recommended within 48 hours) after surgery and all treatment except of bevacizumab should start as soon as wound healing has occurred.
Bevacizumab should start no sooner than 28 days after surgery and should not be initiated within two days of minor surgical procedures (see 6.1.2.1). Intraventricular therapy should not be initiated within five days after placement of the Ommaya reservoir to allow wound-healing (see 6.1.2.1).
In case of postoperative complications and delay of start of treatment another MRI must be obtained before start of antiangiogenic therapy.

6.1.3 Response evaluation week 24 – 28 (T_{24-28})

MRI for response evaluation **is mandatory in week 24 – 28** (see 6.2.4).

The tumor response will be evaluated by neuroradiological and cytological response criteria (see 6.2.3.1 and 6.2.4.3).

CTCAE (see 6.2.6), quality of life (see 6.2.7) and performance status (see 6.2.8) is mandatory in week 24 – 28.

6.2 On-Study Evaluation

6.2.1 Medical history, routine physical and neurologic examination

Medical history since the last visit (in particular headache, vomiting, visual disturbances, seizures, paresthesia), routine physical examination (including blood pressure, weight, auscultation of heart and lungs, palpation of abdomen, inspection of tympanic membrane and throat) and neurologic examination (consciousness, cranial nerve examination, muscle strength, muscle tone, deep tendon reflexes, sensory system testing, finger-to-nose test, assessment of gait) at least every two weeks. Measurement of height is recommended every three months.

6.2.2 Routine laboratory investigations

Weekly (+/- 3 days):
Hematology: White blood count (WBC), relative or absolute neutrophil count (ANC), relative or absolute lymphocyte count (ALC), hemoglobin, platelet count. Absolute neutrophil and absolute lymphocyte count can be calculated from the relative neutrophil or lymphocyte number.
Clinical chemistry: Sodium, albumin, total bilirubin, alanine aminotransferase (ALT), creatinine.
CRP is optional but recommended where possible, since patients might not develop fever or pain under continuous treatment with celecoxib.
Weekly investigations are mandatory for the first six weeks, then at the start of each cycle of oral etoposide/cyclophosphamide, as well as with each dose of bevacizumab.
After discontinuation of oral etoposide/cyclophosphamide intervals may be extended.

Biweekly (every two weeks, +/- 3 days):
Screening dipstick urinalysis for proteinuria each time before bevacizumab is administered.

Monthly (+/- 1 week) or as per institutional guidelines:
Pregnancy tests will be done every 4 weeks or as per institutional guidelines in pubertal females. Pubertal females are defined as any female who has entered puberty (breast or pubic hair Tanner stage II or higher) even if menstruation has not commenced.

Every 3 months (+/- 2 weeks):
Hormones: TSH, T4. Cortisol, T3 and fT4 if clinically indicated or at the investigators discretion.

Given the heavy pretreatment of most patients and the immunosuppression and lymphopenia caused by the proposed drug combination, special attention must be paid to viral and opportunistic infections (particularly pulmonary). Measurement of CRP is optional but recommended, since patients might not develop fever or pain under continuous treatment with celecoxib.

6.2.3 CSF evaluation

Cell count, cerebrospinal fluid protein concentration, and cytology once each cycle of intraventricular therapy. In case of multiple testing pathological findings should be reported.

6.2.3.1 Cytological Response Criteria

Complete response (CR): Complete clearing of all malignant cells from CSF (confirmation of CR by a consecutive negative cytology performed at least two weeks apart).
Stable disease (SD): A negative CSF cytology has not been achieved.

Progressive disease (PD): Occurrence of new malignant cells in two consecutive CSFs after two previous consecutive negative CSF samples.

6.2.4 Routine neuroradiological investigations

6.2.4.1 Time points

Neuroradiological investigations will be performed by MRI with and without gadolinium enhancement at the following time points:

- within two weeks before start of treatment with antiangiogenic therapy
- in case of surgery, a post-operative MRI within 72 hours after surgery must be obtained
- assessment of disease response by MRI should be made every approximately 12 weeks
- **MRI 6 months after start of antiangiogenic treatment (week 24 - 28) is mandatory**

If the clinical condition of the patient necessitates another schedule, neuroradiological examinations may be performed earlier or at different time points.
However, a MRI between week 24 – 28 is mandatory.

6.2.4.2 Neuroradiological guidelines

Imaging Recommendations for Pediatric Brain Tumor Studies (Warmuth-Metz, 2013)

Evaluation of primary tumors of the CNS and possible CNS dissemination is core to their management. Patients entering therapeutic trials must therefore meet and adhere to the minimum imaging requirements for recruitment into the various studies. The most important issue is comparability of pre- and post-operative MRI examinations and subsequent follow up studies. Therefore, if the baseline MRI did not conform to these requirements it should either be repeated preoperatively or the postoperative imaging should be performed in a way (e.g. additional sequences to the standard protocol) that will ensure comparability with the preoperative MRI. This is especially important for brain tumors that show little or no enhancement. In these cases the T2, PD, FLAIR and pre-contrast T1 images should be comparable.

In the case of very small primary, residual or recurrent tumors, measurement of such small structures requires smaller slice thicknesses (3mm or less). In-plane resolution is an essential factor in image quality and therefore a 256 (or preferably 512) matrix is optimal for imaging the brain and a 512 matrix for the spinal canal imaging. The field of view (FOV) should be restricted to about 230 mm for the brain and 350 mm for the spinal MRI.

The tumor and any post-operative residue should be measurable in all 3 planes for the calculation of tumor volume (a x b x c/2). 3D-volume calculations may be performed additionally. These volume calculations are the basis for follow-up evaluation and every effort should be made to ensure the highest possible accuracy.

Cranial MRI:

The standard imaging plane for the brain should be the axial plane (aligned to the anterior-posterior commisural axis). Slice thickness should not exceed 5mm and must be adapted to the individual problem. As the signal of a tumor depends on the field strength of the MRI scanner the field strength must not be changed during the study.

Sequences for 1-1.5 Tesla MR scanners:
Axial T1, T2 and PD or FLAIR (Spin Echo SE technique should be preferred), or as per institutional guidelines.

It is not sufficient to perform either T2 or FLAIR as not all tumors can be depicted equally well on both sequences
Coronal FLAIR
Post contrast axial, coronal and sagittal T1
Axial DWI with ADC

Optional: 3D gradient echo T1 post contrast (particularly for computer guided surgical planning); "functional" imaging (e.g. perfusion (PWI), MR-spectroscopy (MRS), diffusion tensor imaging (DTI) and any other individual local imaging protocols).

Sequences for 3 Tesla MR scanners:
The T1 imaging should be undertaken using a 3D-gradient echo T1 volume sequence pre- and post-contrast in addition to a T1 spin echo (SE), inversion recovery (IR) or gradient echo sequence (e.g. in the axial plane), or as per institutional guidelines.

Spinal MRI:
The entire dural sac must be fully visualized.
Slice thickness for sagittal sequences should not exceed 3 mm or as per institutional guidelines. The physiological veins of the cord can be mistaken for nodules of dissemination and therefore axial slices without gaps (slice thickness can be chosen individually) are essential for all suspicious areas. As fat suppression often leads to artefacts and is not necessary for the delineation of meningeal disease, it should not be used routinely.
Optional:
T2 TSE sequences (particularly when the primary tumor does not enhance or minimally enhances) or fat suppression techniques.

6.2.4.3 Radiological criteria for response
Complete response (CR): Total disappearance of all radiological evidence of tumor determined by two observations not less than four weeks apart. There must be no evidence of CSF positive disease.
Partial response (PR): Regression of ≥50% of all tumor volume (the sum of the products of all measured lesions), determined by two observations not less than four weeks apart. No simultaneous progression of any lesion or the appearance of new lesions may occur. Non-measurable lesions must remain stable or regress for this category. Patients that were CSF negative must remain negative.
Stable disease (SD): Regression of <50% of all tumor volume, or progression <25% of at least four weeks duration. There must be no appearance of new lesions for this category.
Progressive disease (PD): Worsening of disease, evidenced by enlargement of existing tumor(s) of ≥25% in one or more measured lesions or appearance of new disease or the appearance of new CSF positive disease.
No evidence of disease (NED): No recurrence or appearance of new lesions in patient with complete resection and no measurable disease after surgery.

Response by MRI at week 24 – 28 is mandatory and has to be assessed and compared to the baseline scan (T_{-1}).

6.2.5 Determination of tumor/angiogenic markers (optional)
Serum, plasma, CSF and urine should be collected for determination of tumor/angiogenic markers before start of antiangiogenic treatment, one week (+/- 3 days) and one month (+/- 1 week) after start of treatment and at each 12-weekly MRI evaluation.

10mL blood should be collected in a SST (Serum Separator Tube) as well as a plasma/EDTA tube and gently invert 10 times. Leave at room temperature for one hour to coagulate.

Centrifuge at 1100 RCF (g) for 10 minutes*. Transfer the supernatant at 1mL aliquots to several Nunc vials and store at -80°C.
CSF and urine should also be transferred to Nunc vials and stored at -80°C.

* Conversion from RCF to RPM: to determine the centrifuge RPM required for the 1100 RCF (g) use the following formula: n=√ [1100 / (0.00001118 * r)]; where the n is the RPM to which to set the centrifuge, √ is the square root of the formula within the brackets [], and r is the radius distance in cm that is measured from the bottom of the tube when the rotor is spinning, to the center of the rotor (see also Appendix).

6.2.6 Common Terminology Criteria for Adverse Events (CTCAE Version 4.0)

Toxicity will be rated according to the descriptions and grading scales of the Common Terminology Criteria for Adverse Events (CTCAE Version 4.0). Clinically relevant abnormal findings as well as worsening of any pre-existing condition at each visit will be reported as adverse event (AE) if considered clinically significant by the Investigator.

CTCAE Version 4.0 can be downloaded from the internet:
http://evs.nci.nih.gov/ftp1/CTCAE/CTCAE_4.03_2010-06-14_QuickReference_5x7.pdf

6.2.7 Quality of Life

Quality of life will be evaluated at the start of treatment and after 6 months by the KINDL®-questionnaire (Ravens-Sieberer and Bullinger, 1998). This 24 item test is a short, methodologically suitable psychometrically sound and flexible measure to assess health related quality of life in children. The questionnaire can be completed both by children and adolescents, and also by their parents (proxy-version). The KINDL®-questionnaire is available for different age groups and stages of: 4-7 years, 8-11 years, 12 - 16 years and parents. It can be used for both healthy and ill children and can be augmented by a cancer-specific module. The following sub-scale scores can be calculated: 1. physical well-being, 2. emotional well-being, 3. self-esteem, 4. family, 5. friends, 6. school, 7. disease. The time needed to complete the KINDL® questionnaire, as measured empirically in a series of studies, varies between 5 and 15 minutes, depending on the age of the children. The average time for completion is 10 minutes. The questionnaire exists in following languages: German, English, French, Spain, Italian, Dutch, Greek, Turkish, Norway, Swedish, Russian, Serbo-Croatian, Vietnamese, Japanese and Arabic.

6.2.8 Performance status

Performance status will be evaluated at the start of the treatment and after 6 months.

6.2.8.1 Karnofsky performance status

Patient's performance status will be graded according to the following scale:

KPS 100	Normal; no complaints; no evidence of disease
KPS 90	Able to carry on normal activity; minor signs or symptoms of disease
KPS 80	Normal activity with effort; some sign or symptoms of disease
KPS 70	Cares for self; unable to carry on normal activity or do active work
KPS 60	Requires occasional assistance, but is able to care for most personal needs
KPS 50	Requires considerable assistance and frequent medical care
KPS 40	Disabled; requires special care and assistance
KPS 30	Severely disabled; hospitalization is indicated, although death no imminent
KPS 20	Very sick; hospitalization necessary; active support treatment is necessary
KPS 10	Moribund; fatal processes progressing rapidly
KPS 0	Dead

6.2.8.2 Lansky performance status for patients less than 12 years of age

Infant patients' performance status will be graded according to the following scale:

LPS 100	Fully active, normal
LPS 90	Minor restrictions in physically strenuous activity
LPS 80	Active, but tires more quickly
LPS 70	Both greater restriction of and less time spent in play activity
LPS 60	Up and around, but minimal active play; keeps busy with quieter activities
LPS 50	Gets dressed, but lies around much of the day; no active play, able to participate in all quiet play and activities
LPS 40	Mostly in bed; participates in quiet activities
LPS 30	In bed; needs assistance even for quiet play
LPS 20	Often sleeping; play entirely limited to very passive activities
LPS 10	No play; does not get out of bed

6.2.9 Nerve conduction velocity

Nerve conduction velocity may be performed at baseline (T_{-1}) and within the first three months after start of antiangiogenic therapy and every six months thereafter, or if clinical signs of peripheral neuropathy are present. Performance of this test is based on institutional practice and is optional.

6.2.10 Bone age evaluation by X-ray of the left hand (including the wrist)

Bone age should be done yearly in pre-pubertal patients until the end of the follow-up period. Providing a baseline bone age has been performed, subsequent bone age assessments can be omitted only in children whose final growth/bone maturation has unequivocally been reached. Otherwise, bone age should be performed at least once per year during follow up. Depending on the investigator's discretion (i.e., bone pain), bone age may be performed more frequently (i.e., bone age every 6 months).

6.3 Post Treatment Follow-up

Patients should have regular blood work, physical and neurological exams. Patients should have repeated craniospinal MRI images every approximately 12 weeks. Patients who terminate the study prematurely will be asked to continue to participate in the follow-up. The long term follow-up includes patients that continue on therapy, complete therapy, or discontinue therapy for toxicity and will be followed for information approximately every 12 weeks for at least 36 months after initiation of antiangiogenic treatment.

6.4 Drug Administration and Dosing

6.4.1 Bevacizumab

Bevacizumab will be administered every two weeks (+/- 3 days) by intravenous administration at 10 mg/kg, dissolved in 100 mL NaCl 0.9% or as per institutional guidelines. For infants, smaller volumes of NaCl 0.9% may be used. The screening dipstick urinalysis for proteinuria must be <2+ before bevacizumab is administered and systolic and/or diastolic blood pressure below the 95th percentile for age.

The first dose should be administered over 90 minutes, if tolerated, the second dose can be given over 60 minutes and if this is tolerated time of infusion can be reduced to 30 minutes for subsequent bevacizumab administrations. The infusion line should be flushed with NaCl 0.9% after infusion in order to administer the whole bevacizumab dose as per institutional guidelines.

Bevacizumab will be given for one year and should be continued for a second year, or until tumor progression or development of intolerable side effects (see 6.5.1).

6.4.1.1 Infusion related reaction/infusional site extravasation management guidelines

CTCAE grade 1:

If a Grade 1 infusion-related or allergic reaction occurs during the infusion, no treatment is needed. Supervise the patient and complete bevacizumab infusion at a slower rate.

CTCAE grade 2:
When a Grade 2 reaction occurs, stop the bevacizumab infusion. Manage the infusion reaction according to institutional guidelines. After recovery, resume infusion at half the previous infusion rate for 15 minutes. If no further symptoms occur, complete the infusion. Pre-medication (systemic corticosteroid and antihistamine as per institutional guidelines) should be given with the next infusion, but the infusion time should not be reduced.
If infusion-related adverse reactions occur, all subsequent infusions should be administered over the shortest period that was well tolerated. For example:

- If an infusion-related AE occurred after the first administration of bevacizumab, the subsequent (i.e., the second) infusion must be administered over a slower infusion rate. If the infusion is then well tolerated with pre-medication, all subsequent infusions should be delivered over this extended infusion time/the subsequent infusion time may be reduced by 30 ± 10 minutes as long as pre-medication continues to be used. If Grade 2 symptoms recur, bevacizumab should be permanently discontinued.
- When an infusion-related adverse reaction occurs during the infusion or 24 hours after the administration of the second cycle of bevacizumab treatment (i.e., the recommended 60-minute infusion), all subsequent infusions should be administered at an infusion time of 90 ± 15 minutes with pre-medication. If this rate of infusion is well tolerated, the next infusion and all subsequent infusions may be delivered at the same infusion time.
- If an infusion-related reaction occurs with the 30 minute infusion on subsequent cycles (i.e., third cycle and onwards), all subsequent infusions should be administered over 60 minutes with pre-medication.

CTCAE grade 3:
In the event of an anaphylactic reaction occurring during the infusion of bevacizumab, it is suggested that the following steps be taken:

- Stop the bevacizumab infusion.
- Maintain an adequate airway.
- Administer antihistamines, corticosteroids, epinephrine, or other medications as required.
- Continue to observe the patient, document observations, and administer further treatment according to the individual clinical case and clinical judgment.

Permanently discontinue bevacizumab.

CTCAE grade 4:
In the event of an anaphylactic reaction occurring during the infusion of bevacizumab, it is suggested that the following steps be taken:

- Stop the bevacizumab infusion.
- Maintain an adequate airway.
- Administer antihistamines, corticosteroids, epinephrine, or other medications as required.
- Continue to observe the patient, document observations, and administer further treatment according to the individual clinical case and clinical judgment.

Permanently discontinue bevacizumab.

Infusion site extravasation:
When extravasation of bevacizumab occurs during an infusion, it is recommended to take the following actions:

- Discontinue the bevacizumab infusion.
- Treat the extravasation according to institutional guidelines for extravasation of a non-caustic agent.
- If a significant volume of bevacizumab remains in the infusion bag, restart the infusion at a more proximal site ipsilaterally or on the contralateral limb.

6.4.2 Thalidomide
Thalidomide will be initiated at approximately 3 mg/kg or 200 mg qhs (quaque hora somni - "every bedtime"), whichever is lower, based on the available drug formulation. Patients demonstrating toxicity are to decrease their dose to the level necessary to tolerate the drug. Thalidomide will be given (orally) continuously for one year and should be extended for a second year, or until tumor progression or development of intolerable side effects.

6.4.3 Celecoxib
Celecoxib is taken by mouth twice daily. Celecoxib is initiated at 100 mg po bid (morning and prior to bed) in patients 10-35 kg. Patients <10 kg receive 50 mg po bid (pharmaceutical compounding or sprinkled on food). Patients >20 kg can increase the dose to 200 mg po bid as tolerated. Patients >35 kg may be initiated at 200mg po bid and can increase the dose to 300 mg po bid as tolerated. Patients >50 kg can escalate the dose to 400 mg po bid as tolerated. Dose escalations can be initiated every one to two weeks or as tolerated. Dose de-escalation will occur by decreasing back to the last dose that was tolerated.
When celecoxib capsules were taken with a high fat meal, peak plasma levels were delayed for about 1 to 2 hours with an increase in total absorption of 10% to 20%. Celecoxib, at doses up to 200 mg bid can be administered without regard to the timing of meals. Higher doses (400 mg bid) should be administered with food. For patients that have difficulty swallowing pills, pharmaceutical compounding or sprinkled on food is permitted.
Celecoxib will be given for one year and should be extended for a second year, or until tumor progression or development of intolerable side-effects.

6.4.4 Fenofibrate
Fenofibrate will be given at an average daily dose of approximately 90 mg/m^2. Doses will be rounded up or down to the nearest available concentration taking into account the available commercial formulations. This will be taken once daily with food to a maximum dose of 200 mg per day. Dose de-escalation will occur by decreasing back to the last dose that was tolerated. The medication should be taken continuously once daily without interruption. For patients that have difficulty swallowing pills, pharmaceutical compounding or sprinkled on food is permitted. Fenofibrate will be given for one year year and should be extended for a second year, or until tumor progression or development of intolerable side effects.

6.4.5 Etoposide
Etoposide will be given at an average daily dose of approximately 50 mg/m^2 (in the morning or prior to bed). Patients with significant prior episodes of myelosuppression will initiate the oral etoposide at 35 mg/m^2. Doses will be rounded up or down to the nearest available concentration taking into account the available commercial formulations. Patients will take the drug orally once daily for 21 consecutive days. This medication will alternate with oral cyclophosphamide (see 6.4.6) and will continue alternating for one year, or until tumor progression or the development of intolerable side effects. Dose de-escalation will occur by decreasing back to the last dose that was tolerated. If doses are >10% off based on the oral capsule form of etoposide or if patients are unable or unwilling to swallow the capsules, the orally administered, commercially available parenteral form of etoposide containing 20 mg/mL of etoposide or as per institutional guidelines is an alternative.

6.4.6 Cyclophosphamide
Cyclophosphamide should be given at an average daily dose of approximately 2.5 mg/kg or 100 mg po qam (quaque ante meridiem - "every morning"), whichever is lower. Doses will be rounded up or down to the nearest available concentration taking into account the available commercial formulations. Patients will take the drug orally once daily for 21 consecutive days. This medication will alternate with oral etoposide (see 6.4.5) and will continue alternating for one year, or until tumor progression or development of intolerable side effects. Dose de-escalation will occur by decreasing back to the last dose that was tolerated. If doses

are >10% off based on the oral capsule form of cyclophosphamide or if patients are unable or unwilling to swallow the capsules, the commercially available parenteral form of cyclophosphamide is an alternative, when administered orally. Adequate hydration is recommended.

6.4.7 Intraventricular etoposide

Etoposide will be given intraventricularly at a dose of 0.5 mg × 5 days every 4 weeks (+/- 3 days), alternating with aqueous cytarabine/liposomal cytarabine. In infants less than one year, etoposide will be given at a dose of 0.25 mg x 5 days. Intervals of intraventricular therapy may be extended after 6 months if no signs of leptomeningeal dissemination are present and should be maintained if tolerated and if leptomeningeal disease is still present.

6.4.8 Intraventricular cytarabine

Aqueous cytarabine will be administered twice a week for two weeks (days 1, 4, 8, and 11, +/- 3 days), at an intraventricular dose of 30 mg in children older than 3 years (16 mg < 1 year, 20 mg 1-2 years, 26 mg 2-3 years; Schrappe, 2011). If no signs of leptomeningeal dissemination are present in the MRI after three months, administration of aqueous cytarabine may be reduced to twice weekly for one week instead of two weeks out of every four weeks. No concomitant dexamethasone is routinely required during treatment with free aqueous cytarabine.
Liposomal cytarabine will be administered every 4 weeks (+/- 3 days), alternating with intraventricular etoposide.

Intervals between administration of intraventricular therapy may be extended after 6 months when no signs of leptomeningeal dissemination are present and should be maintained if tolerated and if leptomeningeal disease is still present.

6.5 Dose modifications

6.5.1 Bevacizumab

The decision whether to continue bevacizumab administration should be based on the individual patient's circumstances and the physician's judgment that continuation is in the patient's best interest. All dose modifications and the reasons for the changes should be recorded in the chart and on the data form.

6.5.1.1 Treatment delays

Bevacizumab administration should be delayed if any of the following side effects have been observed:

- Hypertension: CTCAE grade 2-3
- Wound healing complications: CTCAE any grade
- Proteinuria: CTCAE grade 3
- Venous thrombosis/embolism (including vascular access device): CTCAE grade 3 and asymptomatic grade 4
- Any other clinically significant (CTCAE grade 3/4) AEs that, according to the physician's discretion, are not clearly associated with chemotherapy and could be related to bevacizumab

Once a patient has met the re-treatment criteria (see 6.5.1.3), bevacizumab administration should recommence.
Bevacizumab should be temporarily withheld in the event of febrile grade 4 neutropenia and/or grade 4 thrombocytopenia (regardless of the relationship to treatment). Regardless of the reason for the delay in bevacizumab, patients must discontinue bevacizumab when the administration of bevacizumab has to be interrupted for more than 6 weeks. When a patient

has to discontinue bevacizumab early because of bevacizumab-related toxicity, he or she should complete therapy without bevacizumab.

6.5.1.2 Bevacizumab discontinuation

Treatment with bevacizumab should be discontinued after a patient has been diagnosed with tumor progression, tumor recurrence, or second primary non-HGG malignancy. Bevacizumab administration should be permanently discontinued if a patient develops any of the following bevacizumab adverse reactions:

- Hypertension: CTCAE grade 4 (hypertensive crisis), hypertensive encephalopathy, medically significant hypertension not controlled with medication
- Left ventricular systolic dysfunction: CTCAE grade 3/4
- Heart failure: CTCAE any grade
- Gastrointestinal perforation: CTCAE any grade
- Tracheo-oesophageal fistula: CTCAE any grade
- Any non-tracheo-oesophageal fistula: CTCAE any grade
- Hemorrhage, non-pulmonary or non-CNS: CTCAE grade 3/4
- Hemorrhage, pulmonary or CNS: CTCAE grade 2/3/4
- Proteinuria: nephrotic syndrome
- Posterior reversible encephalopathy syndrome: any grade
- Venous thrombosis/embolism: CTCAE grade 4 or relapsed grade 3
- Any arterial thrombosis/embolism: CTCAE any grade
- Myocardial infarction: CTCAE any grade
- Cerebrovascular ischemia: transient ischaemic attack, cerebrovascular accident (stroke)

Since the Avastin (bevacizumab) IB is regularly updated to reflect any new safety information, please refer to the latest version of the Avastin (bevacizumab) IB for the most current information on the safety profile of bevacizumab (also see 8.1.14).

6.5.1.3 Toxicity Management Guidelines

Treatment-emergent toxicities must be managed according to national and international guidelines. For management guidelines of expected toxicities see 6.5.1.3.1-6.5.1.3.4.

6.5.1.3.1 Proteinuria (see Appendix)

1. The screening dipstick urinalysis for proteinuria must be <2+ before bevacizumab is administered. For proteinuria grade 2/3 (2+ and higher) see guidelines below.
2. Protein/creatinine ratio has been shown to reflect 24-hour urine protein excretion quite accurately particularly since the first morning specimen eliminates the possibility of postural proteinuria.
3. A referral to a nephrologist is recommended when a patient develops prolonged proteinuria.

CTCAE grade 2/3:

Perform an early morning (first sample) protein/creatinine ratio or 24-hour urinary collection. Delay bevacizumab when clinically significant proteinuria is present (protein/creatinine ratio >190 mg/g or 24-hour urinary protein excretion ≥0.5 g). Resume bevacizumab when either the protein/creatinine ratio <190 mg/g or 24-hour urinary protein excretion <0.5 g. Permanently discontinue bevacizumab if bevacizumab had to be delayed for >6 consecutive weeks.

6.5.1.3.2 Wound complications (non-infectious)/wound dehiscence

CTCAE any grade:

Delay bevacizumab until the wound has satisfactorily healed.

6.5.1.3.3 Hypertension
Age and sex-appropriate systolic and/or diastolic blood pressure that is persistently above the 95th percentile (ULN) requires further evaluation.
It is strongly recommended that patients who develop hypertension during the study be evaluated in conjunction with a (pediatric) cardiologist. In children, blood pressure varies with the age and is closely related to height and weight. Variability in blood pressure in children of similar age and body build should be expected, and it is recommended that serial measurements are obtained when a patient's blood pressure is assessed. Exercise, excitement, coughing, crying, and struggling may raise the systolic pressure of infants and children as much as 40 − 50 mmHg greater than their usual level. Steroid usage (e.g., tumor-related intracranial pressure, allergic reaction, emesis, etc.) may also increase blood pressure, and patients should be weaned off as soon as clinical situation permits. Guidelines to age-specific percentiles of blood pressure are reviewed here (Ingelfinger, 2014).

CTCAE grade 2/3:
1. Delay bevacizumab administration
2. Initiate anti-hypertensive therapy
3. Resume bevacizumab once systolic and diastolic blood pressure for age and sex is below the 95th percentile

CTCAE grade 4 not controlled with medication:
Permanently discontinue bevacizumab.

6.5.1.3.4 Venous thrombo-embolism (including vascular access device):
CTCAE grade 3:
1. Delay bevacizumab
2. Bevacizumab may be resumed once the patient has been anti-coagulated and if the patient has not experienced a grade 3 or 4 haemorrhagic event.
3. Permanently discontinue bevacizumab if the venous thrombo-embolism worsens or recurs after resuming therapy.

Low molecular weight heparin (LMWH) should be prescribed and the treatment monitored in compliance with the approved product labelling or according to local clinical practice guidelines. Similarly, for patients on a coumarin derivative or unfractionated heparin, the INR and aPTT, respectively, should be within therapeutic range.

6.5.2 Thalidomide
The dose of thalidomide should be reduced if toxicity is observed. If the toxicity persists, then continued reduction should be undertaken until the toxicity resolves. Thalidomide may be discontinued in case of intolerable side effects. Once toxicity has resolved, patients can be re-escalated as directed by the treating physician. All dose modifications and the reasons for the changes should be recorded in the chart and on the data form.

6.5.3 Celecoxib
Patients not tolerating the full dose can be decreased to a lower dose level as directed by the treating physician. All dose modifications and the reasons for the changes should be recorded in the chart and on the data form.

6.5.4 Fenofibrate
If ALT is ≥5x normal, fenofibrate is held until the level is <3x normal. Fenofibrate dose will be re-initiated at 50% of the previous dose and escalated to 75% after 1 week, and then 100% after 1 week if the ALT remain <3x normal. All dose modifications and the reasons for the changes should be recorded in the chart and on the data form.

6.5.5 Initiation of etoposide/cyclophosphamide
A 21-day pulse of full dose etoposide or cyclophosphamide may begin when the ANC is ≥1500/mm^3, platelet count is ≥50,000/mm^3 (unsupported), total bilirubin <1.5 mg/dL (<25.5µmol/l) and creatinine <1.5x ULN. If a patient does not meet the criteria above in regards to ANC / PLT count, then etoposide or cyclophosphamide will be started at a lower dose as follows below:
ANC 750 - <1500/mm^3 or Plt 30-50,000/mm^3, give etoposide or cyclophosphamide at 70% of the prior dose or of the full dose for the first cycle. For patients with ANC <750/mm^3 or Plt <30,000/mm^3, hold the etoposide or cyclophsophamide until counts recover. If the counts recover such that the ANC is ≥1500/mm^3, platelet count is ≥50,000/mm^3 (unsupported), then etoposide / cyclophosphamide may be resumed at last dose or full dose for the first cyle. If the ANC recovers to 750 - <1500/mm^3 or Plt 30-50,000/mm^3, in patients who are holding their etoposide or cyclophosphamide, give etoposide or cyclophosphamide at 70% of the prior dose. In case the ANC remains <1500/mm^3 or Plt <30,000/mm^3 for more than one week, please contact the study team in Vienna for further proceeding.
Missed doses are not made up. If ANC / PLT starting criteria are met for the start of the next 21-day pulse of etoposide / cyclophosphamide, dosing can be escalated.

6.5.5.1 Etoposide
Patients not tolerating the 50 mg/m^2/day dose may decrease to 35 mg/m^2/day or less as directed by the treating physician. If at any time during therapy ANC falls to <1500/mm^3, then etoposide will be reduced or held until the ANC returns to ≥1500/mm^3. Missed doses are not made up. All dose modifications and the reasons for the changes should be recorded in the chart and on the data form.

6.5.5.2 Cyclophosphamide
Patients can be dose reduced in 0.5 mg/kg levels as directed by the treating physician until the toxicity resolves. If at any time during therapy ANC falls to <1500/mm^3, then cyclophosphamide will be reduced or held until the ANC returns to ≥1500/mm^3. Missed doses are not made up. All dose modifications and the reasons for the changes should be recorded in the chart and on the data form.

6.5.6 Dose modifications with respect to ANC (Absolute neutrophil count)
If at any time during therapy ANC falls to <1500/mm^3, then etoposide or cyclophosphamide will be reduced or held until the ANC returns to ≥1500/mm^3. Missed doses are not made up. Bevacizumab, thalidomide, fenofibrate, celebrex and intraventricular therapy should be continued.

6.5.7 Dose modifications for elevated transaminases
If ALT is <5x upper limit of normal, therapy with fenofibrate will be continued. If ALT is ≥5x normal, fenofibrate is held until the level is <3x normal. Fenofibrate dose will be re-initiated at 50% of the previous dose and escalated to 75% after 1 week, and then 100% after 1 week if the ALT remain <3x normal.

6.5.8 Intraventricular chemotherapy
In case of intolerable side effects, intraventricular chemotherapy should be reduced in frequency, if the intolerable side effects continue then omitted.

6.6 Supportive Care Guidelines
Patients will receive supportive care for acute or chronic toxicity whenever indicated, including blood components or antibiotics, and other intervention as appropriate according to institutional standards. All treatment administered concurrently should be documented on the case report forms. Particular attention should be paid to treatment that could influence the intended effects or mask side effects of the treatment.

The Common Terminology Criteria for Adverse Events (CTCAE Version 4.0) for grading toxicity are outlined in 6.2.6. For consistency, toxicity for all patients will be quantified using these criteria.

Concurrent supportive care and medications including glucocorticoids and anti-seizure drugs are allowed. Caution should be exercised when patients are given oxcarbazepine due to the risk of hyponatriemia. A switch to levetiracetam at the treating physician's discretion is therefore recommended if possible. Since some antiepileptic drugs may interfere with the cytochrome P450 hepatic enzyme system (e.g. oxcarbazepine, carbamazepine), drug metabolism may change with chronic treatment. Thus, serum anti-seizure medication levels should be monitored as per institutional guidelines.
Caution should be exercised when taking primary psychoactive substances such as benzodiazepines, anti-depressants, or sleep medications due to their sedative effects. Tricyclic anti-depressants will be allowed secondary to their non-sedative nature.

Caution should be exercised when patients are given any other non-steroidal anti-inflammatory drugs (NSAIDs) to avoid increased risk of bleeding.

Clinical assessment of drug toxicities should be performed at each clinic visit and following termination of therapy.

6.6.1 Pneumocystis prophylaxis

It is suggested that all patients be maintained on prophylaxis for *Pneumocystis jirovecii* with trimethoprim / sulfamethoxazole (5 mg/kg/d of trimethoprim in two divided doses given three days per week) or as per institutional guidelines. Other medications can be substituted for patients not tolerating trimethoprim / sulfamethoxazole as per institutional guidelines.

6.6.2 Constipation

Since constipation is a likely complication from treatment with thalidomide, aggressive prophylactic management of bowel function is prudent. If the patient does not have a bowel movement for two days, progress bowel regimen as per institutional guidelines.

6.6.3 Diarrhea

In case of diarrhea, the absorption of drugs may be impaired. If the patient suffers from severe diarrhea, loperamide may be administered (as per institutional guidelines). Since toxic megacolon might occur following loperamide, its use in infectious gastroenteritis should be avoided when features of mucosal involvement are present (McGregor, 2007; Kato, 2007).

6.6.4 Antiemetic

Antiemetic prophylaxis with a 5-hydroxytryptamine$_3$-antagonist can be used during antiangiogenic therapy. Other agents may be used as per institutional guidelines.

6.6.5 Proton pump inhibitor

Proton pump inhibitor can be prescribed in order to prevent potential gastrointestinal bleeding due to celecoxib.

6.6.6 Growth factors

Hematopoietic growth factors should not be administered prophylactically. However, therapeutic use of G- or GM-CSF in patients with serious neutropenic complications such as sepsis may be considered at the investigator's discretion.

6.7 Patient identification

Each enrolled patient will be identified by a code for the center and given a consecutive number.

7 Analysis section

This study is a prospective, multi-center phase-II study, not blinded and not randomized in three independent strata. The aim of this study is to examine the effectiveness of an antiangiogenic multidrug-regime for recurrent medulloblastoma, ependymoma or ATRT. The therapy is regarded as effective, if the response rate (CR – complete remission, PR – partial remission, SD – stable disease) or lack of signs of recurrence after gross total resection is greater than 35 per cent. In total, 40 patients will be enrolled in the study in Stratum I, 30 patients will be enrolled in Stratum II, and 30 patients will be enrolled in Stratum III.
The planned recruiting time is 60 months; follow-up time for each patient will last at least 24 months or until death occurs. The following questions will be examined within this study.

7.1. Formulation of hypotheses

7.1.1 Main question

Is the antiangiogenic multidrug-regime with thalidomide, celecoxib, etoposide, cyclophosphamide, fenofibrate, and bevacizumab, augmented with intraventricular etoposide and aqueous cytarabine/liposomal cytarabine, effective in the treatment of recurrent medulloblastoma/ependymoma/ATRT? The therapy regime is judged as effective in each stratum if the rate of response 6 months after start of antiangiogenic treatment is greater than 35 per cent. Response is defined on the basis of neuroradiological and cytological findings.

7.1.2 Additional questions

1. What is the overall survival rate as determined from the beginning of treatment with this antiangiogenic multidrug-regimen?
2. What is the progression free survival rate as determined from the beginning of treatment with this antiangiogenic multidrug-regimen?
3. What is the toxicity (frequency and severity) with this antiangiogenic multidrug-regimen in accordance to CTCAE (Version 4.0)?
4. What is the quality of life of patients treated with this antiangiogenic multidrug-regimen? Is there a change in the quality of life from beginning of the treatment with this antiangiogenic multidrug-regimen to the evaluation after 6 months?
5. What is the performance status of patients treated with this antiangiogenic multidrug-regimen? Is there a change in the performance status from beginning of the treatment with this antiangiogenic multidrug-regimen to the evaluation after 6 months?
6. The following variables will be evaluated with regard to their influence on outcome: Tumor biology (histology, molecular subgroups, metastatic stage, age at first diagnosis [<3 years, >3 years]), tumor size, age at start of antiangiogenic therapy, sex, duration of remission prior to antiangiogenic therapy, number of recurrences, resection / extent of resection prior to antiangiogenic therapy, additional therapy (e.g. RTX), previous radiotherapy, switch to aqueous cytarabine for intraventricular therapy.
7. Examination of tumor/angiogenic markers.

7.2 Statistical evaluation

Evaluable patients will be studied.
The main question of this study will be examined using a two-sided analysis with a local level of significance of 0.05. The p-values for the examination of the additional questions and the per-protocol-evaluation are explorative.

7.2.1 Main hypothesis

The following main hypothesis will be tested (π: probability for response, lack of recurrence after gross total resection):
H0: $\pi \leq 15\%$ The study therapy is considered as ineffective (type I error rate $\alpha=0.05$, two sided).

H1: $\pi \geq 35\%$ The study therapy is considered as effective (type II error rate $\beta=0.10$ in Stratum I, $\beta=0.20$ in Stratum II+III), further clinical studies are warranted.

Decision rule –Stratum I:

1. Stage 1:
- In the first stage, 16 patients are recruited and their response to therapy will be determined.
- If one or fewer of the 16 patients shows a response to therapy, this study with the retention of the null hypothesis will be terminated.
- If at least two patients show a response to therapy, the study is resumed and a further 11 patients will be recruited into stage 2 (see below).

2. Stage 2:
- In the second stage, the response to therapy of 27 patients is determined.
- If 4 or fewer of the 27 patients demonstrate a response to therapy, this study with the retention of the null hypothesis will be terminated.
- If at least five patients show a response to therapy, the study will be resumed and a further 11 patients will be recruited to stage 3 (see below).

3. Stage 3:
- If 9 or fewer of the 38 recruited patients show a response to therapy, then the study will be terminated with the retention of the null hypothesis.
- If ten or more patients demonstrate a response to therapy, the null hypothesis will be rejected and the alternative hypothesis is accepted.

Decision rule –Stratum II+III:

1. Stage 1:
- In the first stage, 11 patients are recruited and their response to therapy will be determined.
- If one or fewer of the 11 patients shows a response to therapy, this study with the retention of the null hypothesis will be terminated.
- If at least two patients show a response to therapy, the study is resumed and a further 9 patients will be recruited into stage 2 (see below).

2. Stage 2:
- In the second stage, the response to therapy of 20 patients is determined.
- If 3 or fewer of the 20 patients demonstrate a response to therapy, this study with the retention of the null hypothesis will be terminated.
- If at least four patients show a response to therapy, the study will be resumed and a further 8 patients will be recruited to stage 3 (see below).

3. Stage 3:
- If 7 or fewer of the 28 recruited patients show a response to therapy, then the study will be terminated with the retention of the null hypothesis.
- If eight or more patients demonstrate a response to therapy, the null hypothesis will be rejected and the alternative hypothesis is accepted.

Our design was developed given the results of former studies with multimodal therapeutic regimens including high-dose chemotherapy and autologous stem cell rescue in children with recurrent embryonal tumors and ependymomas.

Medulloblastoma

A response rate of about 26 per cent for patients with recurrent medulloblastoma, CNS-PNET and ependymoma was reported (Kalifa, 1992). Out of a cohort of 27 malignant CNS relapses including 13 medulloblastomas, 81 per cent of the patients presented with progressive disease after a median time of 4 months (Gururangan, 2008). Altogether, only 2 patients showed partial response with therapy, with 2 therapy related deaths. The estimated progression free survival after 6 months was 30 - 40 per cent. Younger patients without prior irradiation had the best results. Kieran et al. (2005) demonstrated a progression free survival rate after 6

months of approximately 40 per cent in children with various recurrent tumors following an antiangiogenic therapy regimen.

Ependymoma
Robison et al (2013) used a multi-agent oral antiangiogenic (metronomic) regime to treat children with recurrent or progressive cancer. Seven ependymoma patients (37%) completed therapy with SD or better, with a 2-year PFS of 34% and OS of 43%. In retrospective analyses, recurrences of ependymoma treated by various approaches (surgery, irradiation, oral chemotherapy, conventional chemotherapy), a 5-year OS of 37% and 33% was reported. (Antony, 2014; Tippelt, 2016).

ATRT
Fouladi et al. (2006) reported no objective responses in recurrent ATRT treated with oxaliplatin. The use of alisertib in 4 patients with recurrent ATRT showed CR in one patient and PR in 2 patients, with a mean PFS of 14.5 months (Wetmore, 2015). Sustained complete response to metronomic chemotherapy could be achieved in a patient with refractory ATRT (Berland, 2017).

In summary, tumor progression occurs very rapidly in patients with recurrent /progressive medulloblastoma, ependymoma and ATRT, and new therapeutic approaches are needed. Obviously, patients in later relapse will bias the results to more rapid progression. However, this is the group of patients we are treating.
In contrast to the studies mentioned above using conventional and high dose chemotherapy, patients with multiple relapses will be included in this study. We anticipate that therapy will be better tolerated and will be associated with fewer hospitalizations and improved quality of life when compared to regimens using conventional or high dose chemotherapy.

7.2.2 Additional hypothesis

1. Kaplan Meier survival estimates of overall survival rates after 6, 12, 24, and 36 months with 95% confidence intervals will be evaluated and compared to historical samples (Windelberg, 2007; Fleischhack, 2007; Fleischhack 2008; Pizer, 2011, Sabel, 2016, Robison 2013; Antony, 2014; Tippelt, 2016, Fouladi, 2006; Wetmore 2015; Berland 2017).
2. Kaplan Meier estimates of progression free survival after 6, 12, 24, and 36 months with 95% confidence intervals will be evaluated and compared to historical samples (Windelberg, 2007; Fleischhack, 2007; Fleischhack 2008; Pizer, 2011; Sabel 2016;).
3. The number and relative frequency of toxicities (CTCAE Version grade 3, 4 and 5) will be evaluated every 12 weeks.
4. Performance status will be compared from the start of therapy and after 6 months of therapy.
5. Subscale-scores and total scores of the KINDL assessments at the start of therapy, after 6 months of therapy will be evaluated.
6. The aforementioned variables will be evaluated comparing their influence on response rate, overall survival rate, progression free survival rate, toxicity, quality of life and performance status by multivariate cox-regression.
7. Examination of angiogenic factors (e.g. VEGF, EPC and prostaglandin E2 levels) will be descriptive.

7.3 Interruption due to toxicities

The systematic evaluation of organ toxicities will use the Common Terminology Criteria for Adverse Events (CTCAE Version 4.0; see 6.2.6). Hematological toxicity is determined by blood count controls, further toxicity by laboratory examinations and clinical investigations. Due to multiple chemo- and radiotherapeutic pretreatment in this patient population, accumulated toxicities with continued therapy are to be expected. Toxicities grade 1 and 2 will only be collected descriptively and reported in the eCRF if clinically significant. With

toxicities ≥ grade 3, dose modification or discontinuation of certain agents is likely to be required. Only medications thought responsible for the toxicity will be dose reduced or discontinued, based on the known side effect profile of each medication. Patients do not need to come off protocol if one or more drugs are dose reduced or discontinued because of toxicity.

7.4 Interim analyses and end analysis

Stratum I: The first interim analysis of data will be performed following the MRI after 6 months of treatment (week 24-28) of the 16th patient. If response (CR, PR, SD) or lack of recurrence after surgery is observed in one or fewer of the first 16 patients at this time, then the study will be terminated prematurely and the null hypothesis is retained. If response (CR, PR, SD) or lack of recurrence is observed in at least two of the first 16 patients at this time, then an additional 11 patients will be accrued and a second interim analysis performed. The study will be terminated prematurely if 4 or fewer of the 27 patients are responders to the therapy. If there are at least 5 responders at this time, the study will accrue an additional 11 patients. An additional interim analysis will be performed with 38 patients. The alternative hypothesis will be accepted if there are at least 7 responders during the first interim analysis or at least 10 responders during the second interim analysis or this additional analysis. If the alternative hypothesis is accepted during first or second interim analysis, the study will continue to accrue patients as planned until the end analysis. Sequential characteristic: In the case of a clinically insufficient response rate of only 15 percent early termination is initiated with a probability of 29 per cent after 16 patients and with a probability of 64 percent after 27 patients. The expected number of patients is therefore 27 if the therapy is ineffective with a response rate of 15 percent.

Stratum II+III: The first interim analysis of data will be performed following the MRI after 6 months of treatment (week 24-28) of the 11th patient in each stratum. If response (CR, PR, SD) or lack of recurrence after surgery is observed in one or fewer of the first 11 patients at this time, then the study will be terminated prematurely and the null hypothesis is retained. If response (CR, PR, SD) or lack of recurrence is observed in at least two of the first 11 patients at this time, then an additional 9 patients will be accrued and a second interim analysis performed. The study will be terminated prematurely if 3 or fewer of the 20 patients are responders to the therapy. If there are at least 4 responders at this time, the study will accrue an additional 8 patients. An additional interim analysis will be performed with 28 patients. The alternative hypothesis will be accepted if there are at least 6 responders during the first interim analysis or at least 7 responders during the second interim analysis or this additional analysis. If the alternative hypothesis is accepted during first or second interim analysis, the study will continue to accrue patients as planned until the end analysis. Sequential characteristic: In the case of a clinically insufficient response rate of only 15 per cent early termination is initiated with a probability of 49 per cent after 11 patients and with a probability of 72 per cent after 20 patients. The expected number of patients is therefore 20 if the therapy is ineffective with a response rate of 15 per cent.

7.5 Sample size

Stratum I: The aim of this study is to examine if the response rate of an antiangiogenic multidrug-regime 6 months after start of treatment for recurrent medulloblastoma is over 35 per cent. With a level of significance of 5 per cent (α=0.05), a recruiting time of 60 months, a treatment duration of at least 12 months, a follow-up time of 24 months, and a dropout rate of 5 percent, 40 patients are necessary to reach an acceptable test power of 90 per cent (1-β=0.90) for the execution of a minimax three-stage design for phase II oncology clinical trials according to Chen and Shan (2008). The clinically sufficient response rate was specified with $\pi1$=35%; the clinically insufficient response rate with $\pi0$=15%. That corresponds to an annual recruiting rate of 8 patients.

Stratum II+III: The aim of this study is to examine if the response rate of an antiangiogenic multidrug-regime 6 months after start of treatment for recurrent medulloblastoma is over 35 percent. With a level of significance of 5 per cent (α=0.05), a recruiting time of 60 months, a treatment duration of at least 12 months, a follow-up time of 24 months, and a dropout rate of 5 percent, 30 patients are necessary to reach an acceptable test power of 80 per cent (1-β=0.80) for the execution of a minimax three-stage design for phase II oncology clinical trials according to Chen and Shan (2008). The clinically sufficient response rate was specified with $\pi 1$=35%; the clinically insufficient response rate with $\pi 0$=15%. That corresponds to an annual recruiting rate of 6 patients in each stratum.

7.6 Relevant protocol deviations

Patients that discontinue therapy for reasons other than progression, toxicity or going for an additional therapy for patients who achieved complete remission (e.g. transplant) prior to completing their 6 month evaluation will be considered inevaluable for the current assessment and will be replaced. All protocol deviations will be listed in the study report.

8 Study drugs

This section lists the most relevant drug actions and side effects, information for application and supportive measures. The investigational medicinal products used in the study will be labeled in a GMP certified pharmacy according to regulatory requirements or as per institutional guidelines.
These guidelines do not exempt the treating physician from his / her obligation to inform himself / herself about the latest experiences with the respective drugs by use of the most recent publications and the information material provided by the drug companies, especially concerning the range of possible drug interactions.
Despite the fact that chemotherapy within this protocol is designed primarily for heavily pretreated patients, its use can have a profound impact upon the patients' general prognosis and daily life. Thus, the intensity of this protocol is justified, but requires responsible monitoring to avoid uncontrolled or severe, progressive side effects.

8.1 Bevacizumab

8.1.1 Description

Bevacizumab is a recombinant humanized anti-VEGF monoclonal antibody, consisting of 93% human and 7% murine amino acid sequences. The agent is composed of human IgG framework and murine antigen-binding complementarity-determining regions. The estimated adult half-life of bevacizumab is approximately 20 days (range 11-50 days). The predicted time to reach steady state was 100 days in 491 patients who received 1 to 20 mg/kg weekly, every 2 weeks, or every 3 weeks.

8.1.2 Classification

Recombinant humanized monoclonal antibody.

8.1.3 Molecular Weight

Approximate molecular weight is 149,000 daltons.

8.1.4 Mode of Action

Bevacizumab blocks the binding of vascular endothelial growth factor (VEGF) to its receptors resulting in inhibition of angiogenesis.

8.1.5 Availability and drug storage conditions

25 mg/mL concentrate for solution for infusion is available. The necessary amount of bevacizumab should be withdrawn and diluted to the required administration volume with sodium chloride 9 mg/mL (0.9%) solution for injection. Store in a refrigerator (2°C - 8°C). Do not freeze. Keep the vial in the outer carton in order to protect from light. In-use storage times and conditions should not be longer than 24 hours at 2°C to 8°C.

8.1.6 Stability

The sterile single use vials contain no antibacterial preservatives. Therefore, discard vials 8 hours after initial entry or as per institutional standard procedure.

8.1.7 Preparation

Vials contain no preservatives and are intended for single use only. Dilute the dose volume in 0.9% sodium chloride for injection to a final concentration within the range of 1.4-16.5 mg/mL. It is recommended that bevacizumab be administered within 8 hours after preparation; if this is not feasible, in-use storage times and conditions are the responsibility of the user and would normally not be longer than 24 hours at 2°C−8°C, unless dilution has taken place in controlled and validated aseptic conditions. Bevacizumab is incompatible with D_5W (solution of 5% dextrose in water).

8.1.8 Route of Administration
Intravenous.

8.1.9 Administration
Bevacizumab is administered at 10 mg/kg/dose approximately every two weeks (+/- 3 days) by intravenous infusion.

8.1.10 Premedication
Routine premedication is not required for the first dose of bevacizumab. Bevacizumab is incompatible with D_5W (the drug is inactivated).

8.1.11 Application
Intravenous infusion in 100 mL NaCl 0.9% for 30 minutes. For infants, smaller volumes may be used. The first dose can be administered over 90 minutes, if tolerated, the second dose can be given over 60 minutes and if this is tolerated time of infusion should be reduced to 30 minutes for subsequent bevacizumab administrations. The infusion line should be flushed with NaCl 0.9% after infusion in order to administer the whole bevacizumab dose or as per institutional guidelines. Bevacizumab will be given for one year and should be extended for a second year, or until tumor progression or development of intolerable side effects.

8.1.12 Vital Signs
Check vital signs prior to infusion and monitor for infusion-related reactions as per institutional standards.

8.1.13 Infusion reactions
See 6.4.1.1.

8.1.14 Frequencies of Toxicities

	**Common** Happens to 21-100 children out of every 100	**Occasional** Happens to 5-20 children out of every 100	**Rare** Happens to <5 children out of every 100
**Immediate:** Within 1-2 days of receiving drug	Asthenia, hypertension, nausea, vomiting, anorexia, diarrhea, constipation, cough, pruritis, itching, headache, dyspnea	Dyspepsia, fever, fatigue, lethargy, malaise, asthenia, chest, back and abdominal pain, dizziness, rhinitis, hypertension	Infusion reactions and anaphylaxis: (dyspnea, $\downarrow O_2$, bronchospasm, wheezing, hypertensive crisis, chest pain, muscle pain, pain NOS, rigors, headache, diaphoresis, syncope, hypotension), flatulence, dry mouth, taste disorder, tearing, rash, urticaria, hives, drowsiness, acidosis, allergic reaction, rigors, chills, acute infusion reaction

	**Common**<br>Happens to 21-100 children out of every 100	**Occasional**<br>Happens to 5-20 children out of every 100	**Rare**<br>Happens to <5 children out of every 100
**Prompt:**<br>Within 2-3 weeks, prior to next course	Epistaxis, proteinuria, infection, myalgia, arthralgia, neutropenia when added to chemotherapy regimens, allergic rhinitis	Stomatitis, mucositis, hematuria, ulceration, upper respiratory infection, GI hemorrhage, GI ulcer, alopecia, weight loss, sinusitis, anemia, neutropenia, leucopenia, voice change, depression, UTI, febrile, rash, desquamation, vertigo	Intra-abdominal thrombosis, DVT, arterial thromboembolic events (CVA, MI, TIA, angina), cerbrovascular ischemia, visceral arterial ischemia, supraventricular arrhythmia, acute coronary syndrome, ventricular fibrillation, cardiac ischemia/infarction, cardiac troponin elevation, left ventricular diastolic dysfunction, left ventricular systolic dysfunction, exfoliative dermatitis, skin ulcer, ↑ AST/ALT, ↑ creatinine, ↑ alkyline phosphatase, hemorrhage (pulmonary, nasal, CNS, GU, GI, vaginal), colitis, GI perforation (L), GI ulcer, GI obstruction, ileus, hypokalemia, hyperkalemia, hyperbilirubinemia, thrombocytopenia, thrombosis, hyperesthesia, paresthesia, Reversible Posterior Leukoencephalopathy Syndrome (RPLS), Posterior Reversible Encephalopathy Syndrome (PRES) or similar leukoencephalopathy syndrome, fatal infections when used in combination with chemotherapy regimens, heartburn/dyspepsia, infection with normal ANC
**Delayed:**<br>Any time later during therapy, excluding the above conditions		Nasal-septal perforation	Wound dehiscence, wound complications, CHF (L), nephrotic syndrome, fistula of the GI tract including the esophagus, trachea or main bronchus, renal injury or failure, urinary fistula, vaginal fistula, necrotizing fasciitis, osteonecrosis, tumor necrosis leading to pneumothorax, leakage of anastamoses, sinusitis, voice change, dysarthria, bone metaphyseal dysplasia, ovarial/testicular dysfunction, aneurysm, necrotizing fasciitis

	**Common** Happens to 21-100 children out of every 100	**Occasional** Happens to 5-20 children out of every 100	**Rare** Happens to <5 children out of every 100
**Unknown Frequency and Timing**:	Bevacizumab has been shown to be teratogenic in rabbits when administered in doses that are two-fold greater than the recommended human dose on a mg/kg basis. Observed effects included decreases in maternal and fetal body weights, an increased number of fetal resorption, and an increased incidence of specific gross and skeletal fetal alterations. Adverse fetal outcomes were observed at all doses tested. Angiogenesis is critical to fetal development and the inhibition of angiogenesis following administration of bevacizumab is likely to result in adverse effects on pregnancy. It is not known whether bevacizumab is secreted in human milk. Because human IgG1 is secreted into human milk, the potential for absorption and harm to the infant after ingestion is unknown.		

8.1.14.1 Additional adverse events, regardless of causality
Blood/Bone marrow: Idiopathic thrombocytopenic purpura;
Cardiac general: Cardiac arrest; pericardial effusion; pulmonary hypertension
Coagulation: Disseminated intravascular coagulation
Death: Sudden death (cause unknown)
Dermatology/Skin: Hypopigmentation
Gastrointestinal: Rectal abscess/necrosis; small bowel obstruction; taste alteration
Metabolic/Laboratory: Hyperglycemia; hypoglycemia; hypomagnesemia; hyponatremia
Muscosceletal/Soft tissue: Aseptic necrotic bone; myasthenia gravis
Neurology: Aseptic meningitis; confusion; peripheral neuropathy; seizure; syncope
Ocular/Visual: Cataract; watery eye
Pulmonary/Upper respiratory: Acute respiratory distress syndrome; pneumonitis/pulmonary infiltrates; pneumothorax
Note: Bevacizumab in combination with other agents could cause an exacerbation of any adverse event currently known to be caused by the other agent, or the combination may result in events never previously associated with either agent.

8.1.14.2 CNS hemorrhage (punctuate lesions)
Patients with punctate hemorrhage will be allowed to initiate or continue bevacizumab therapy but will be closely monitored for signs and symptoms of worsening hemorrhage. Any non-surgical hemorrhage > 1cm should result in holding bevacizumab administration.

8.1.15 Method of Procurement
Bevacizumab is commercially available and will be prelabeled by pharmacy as per institutional standards (according to national law). Drug Batch Numbers and expiry date will be documented in the study drug accountability log. Used and unused vials will be checked by the study monitor and thereafter disposed of as per local practice.

8.2 Thalidomide

8.2.1 Description
The empirical formula for thalidomide is $C_{13}O_4N_2H_9$ and the gram molecular weight is 258.2 g/mol. The CAS number of thalidomide is 50-35-1. Thalidomide is an off white to white, nearly odorless, crystalline powder that is soluble at 25°C in dimethyl sulfoxide and sparingly soluble in water and ethanol. The glutarimide moiety contains a single asymmetric center and, therefore, may exist in either of two optically active forms designated S(-) or R(+). Thalidomide is an equal mixture of the S(-) and R(+) forms and, therefore, has net optical rotation of zero.

8.2.2 Formulation

Thalidomide is available in 25, 50, 100 and 200 mg capsules for oral administration. Active ingredient: thalidomide. Inactive ingredients: anhydrous lactose, microcrystalline cellulose, polyvinylpyrrolidone, stearic acid, colloidal anhydrous silica, and gelatin.

8.2.3 Reconstitution

None. Capsules should not be opened. Thalidomide has been shown to be stable for up to 36 months when stored under ambient conditions. Over this time period, the capsules show no significant loss in potency and no increase in degradation products. Thalidomide has been shown also to be stable when stored under accelerated conditions (40°C/75% relative humidity, 3 months). Clinical supplies should be retained in a secure, cool dry place.

8.2.4 Application

Thalidomide will be initiated at 3 mg/kg or 200 mg qhs (taken 1-2 hours before bed), whichever is lower.

8.2.5 Pharmacology

Thalidomide is a non-polar agent that can effectively cross membranes. Non-enzymatic cleavage of 1 or more of the amide bonds results in the generation of at least 1 carboxyl group, which gives the molecule a more polar nature and results in less efficient crossing of biological membranes. Given the possible combinations of hydrolysis, hydroxylation and optical activity, there may be more than 50 metabolites of thalidomide *in vivo*. Experimental studies in animals have shown high concentrations of thalidomide in the gastrointestinal tract, liver and kidney, and lower concentrations in muscle, brain and adipose tissue. Thalidomide is able to cross the placental barrier. In animals, the main pathway of degradation appears to be non-enzymatic hydrolytic cleavage. Minor amounts of hydroxylated products have been detected in the urine of some species. Hepatic metabolism of thalidomide is thought to involve enzymes of the cytochrome P450 family. Only the parent compound is enzymatically modified.

Thalidomide itself does not cause enzyme induction, although it may interfere with enzyme induction caused by other compounds. Oral administration of thalidomide at 100 to 200 mg in humans results in maximal blood concentrations of 0.9 to 1.5 mg/L after 4 to 6h (Chen, 1989). Absorption and elimination half-lives calculated from data of 8 healthy subjects were 1.7 + 1.05 and 8.7 + 4.11 h, respectively; a lag time of 0.41 + 0.17 h was observed in 6 individuals. Using a 1-compartment model, the authors calculated a volume of distribution of 120.64 + 45.36L, a total body clearance of 10.41+ 2.04 L/h, and a renal clearance of 0.08 + 0.03 L/h. Only 0.6 + 0.22% of the administered dose was excreted as unchanged compound in the urine. The hydrolytic cleavage in serum is much slower than that *in vitro* at pH 7.4. This may be because thalidomide is highly bound to plasma proteins. Drug interactions with thalidomide have not been systematically studied. Thalidomide enhances the activity of barbiturates, alcohol, chlorpromazine and reserpine, while its sedative action is antagonized by methyl amphetamine and methylphenidate.

8.2.6 Toxicity

Based on its known immunosuppressive effects, thalidomide has been tested for activity in a number of diseases including acute and chronic graft vs host disease, leprosy, rheumatoid arthritis, Behcet's disease and recurrent aphthosis in both HIV infected and non-infected individuals. From this large experience the toxicities of the drug are well known (although not necessarily the mechanism). The most worrisome of these toxicities is a peripheral neuropathy secondary to axonal dropout. The neuropathy clinically resembles that seen with the vinca alkaloids, and is generally not progressive if therapy is terminated in a timely manner. Continued treatment, however, can result in permanent neurologic damage, particularly in patients with a baseline neuropathy. Other neurologic side effects previously reported are somnolence, confusion, frank encephalopathy (the latter only having been seen in patients

with AIDS). Other reported side effects have included constipation, swelling of the limbs, erythema of the limbs, hair loss, fever, rash, increased appetite, loss of libido, nausea, and amenorrhea, dizziness, headaches, hypotension, leg cramps, rhinitis and diarrhea.
Serious dermatologic reactions including toxic epidermal necrolysis and Stevens-Johnson syndrome, which may be fatal, have been reported in association with thalidomide therapy. Thalomide should be discontinued if a skin rash occurs and only resumed following appropriate clinical evaluation. If the rash is purpuric, vasculitic, exfoliative, or bullous, or if Stevens-Johnson syndrome or toxic epidermal necrolysis is suspected, use of thalidomide should not be resumed.
Thalidomide can cause severe birth defects in humans. Women and men taking thalidomide must take special precautions, and be willing and able to comply with all aspects of the FDA-mandated S.T.E.P.S.® program (USA).

8.2.6.1 Neurologic
The most common side effect is drowsiness (thalidomide was originally used as a sedative) and sensory peripheral neuropathy (axonal in origin), that usually resolves if therapy is terminated. Rarely, this can be permanent, although it is unclear whether thalidomide was responsible. Secondary to the sedative effects of thalidomide, patients should be warned against driving a motorized vehicle while on study.

8.2.6.2 Teratogenicity
Thalidomide causes profound birth defects when taken by pregnant women. The characteristic defect is dysmelia. The drug is strictly contraindicated in pregnant individuals.

8.2.6.3 Miscellaneous
A number of side effects other than neurologic have been occasionally reported, most prominently those related to the anti-cholinergic activity of the drug. Reported adverse reactions include constipation, confusion, increased appetite, decreased libido, nausea, pruritis, swelling of limbs, erythema of limbs, hair loss, fever, rash, hypotension, leg cramps, rhinitis, eosinophilia, amenorrhea, dry mouth, tremors, blood clots, colitis, severe infection that may be serious or life-threatening, and wounds, cuts and bruises may not heal as quickly.
Although not reported from pre-marketing controlled clinical trials, seizures, including grand mal convulsions, have been reported during post-approval use of thalidomide in clinical practice. Because these events are reported voluntarily from a population of unknown size, estimates of frequency cannot be made. Most patients had disorders that may have predisposed them to seizure activity, and it is not currently known whether thalidomide has any epileptogenic influence. During therapy with thalidomide, patients with a history of seizures or with other risk factors for the development of seizures should be monitored closely for clinical changes that could precipitate acute seizure activity.

Men taking thalidomide must use latex condoms every time they have sex with women since thalidomide may be found in semen.
This medicine is for subject use ONLY. IT SHOULD NOT BE SHARED WITH ANYONE.
It should be safely stored. It must be kept out of the reach of children and should never be given to women who are able to have children, and used only as directed by the physician.
Do not drink alcohol or take any other medicine that has not been prescribed by the doctor, especially nonprescription drugs that makes the subject sleepy.

Women:
Thalidomide does not induce abortion of the fetus and should never be used for contraception. The subject has been informed and understands the risk of birth defects, and that she agrees not to become pregnant while taking thalidomide.

If there is ANY chance that the subject can get pregnant, she must begin TWO methods of birth control 4 weeks before she starts taking thalidomide, as outlined in S.T.E.P.S.®, unless she completely abstains from reproductive heterosexual intercourse.
The doctor must give the subject a pregnancy test 24 hours before she begins taking thalidomide. If the subject is pregnant, she cannot take thalidomide.
Female subjects of child bearing potential will have pregnancy tests before and during treatment as outlined in S.T.E.P.S.®, even if she agrees not to have reproductive heterosexual intercourse.
The subject will be given information about the following acceptable birth control methods:
Effective Methods

• Intrauterine device (IUD)
• Latex condom
• Hormonal (birth control pills, injections, implants)
• Diaphragm
• Tubal ligation
• Cervical Cap
• Partner's vasectomy

Remember: The subject must use at least one highly effective method and one additional effective method AT THE SAME TIME. However, the doctor may recommend that the subject will use two barrier methods for medical reasons.
These birth control methods must be initiated 4 weeks before starting thalidomide therapy (unless the patient completely abstains from reproductive heterosexual intercourse), and must be used all during thalidomide therapy and for at least 4 weeks after thalidomide therapy has stopped.
The subject must talk to the doctor before changing any birth control methods she has already agreed to use.
The subject must have a pregnancy test done by the doctor every 4 weeks. The subject may also need to have a pregnancy test if she misses her period or has unusual menstrual bleeding.
The subject may also need to have a pregnancy test if she misses her period or have unusual menstrual bleeding.
If the subject has sex without birth control or if for any reason she thinks she may be pregnant, she must IMMEDIATELY stop taking thalidomide and tell the doctor.
If the subject gets pregnant, she must IMMEDIATELY stop taking thalidomide. Contact the doctor immediately to discuss the pregnancy.
The subject must not breast-feed a baby while she is being treated with thalidomide.

The subject must NEVER donate blood or ova while she is being treated with thalidomide.

Men:
The subject has been informed and understands the risk of birth defects, and that he agrees to NEVER have sex with a woman unless he uses a latex condom.
The subject must use a latex condom every time he has sex with a woman while he is taking thalidomide and for 4 weeks after he stopped taking the drug.
The subject must tell the doctor if he has sex with a woman without using a latex condom, or if he thinks for any reason that his partner may be pregnant.

The subject must NOT be a sperm or blood donor while he is being treated with thalidomide.

8.2.7 Frequencies of Toxicities

		Common Happens to 21-100 patients out of every 100	Occasional Happens to 5-20 patients out of every 100	Rare Happens to <5 patients out of every 100
Body as a Whole				
				Abdominal pain (3.1%)
		Asthenia (21.9%)		
			Chills (9.4%)	
		Fever (21.9%)		
			Headache (18.7%)	
			Infection (6.3%)	
				Pain (3.1%)
Digestive System				
			Anorexia (9.4%)	
			Constipation (9.4%)	
			Diarrhea (18.7%)	
			Dry mouth (9.4%)	
			Liver function tests multiple abnormalities (9.4%)	
			Nausea (12.5%)	
			Oral moniliasis (6.3%)	
Hemic and Lymphatic				
			Anemia (12.5%)	
		Leukopenia (25.0%)		
			Lymphadenopathy (12.5%)	
Metabolic and Endocrine Disorders				
				Edema peripheral (3.1%)
			Hyperlipemia (9.4%)	
			AST increased (12.5%)	
Nervous System				
			Agitation (9.4%)	
			Dizziness (18.7%)	
			Insomnia (9.4%)	
			Nervousness (9.4%)	
			Paresthesia (15.6%)	
		Somnolence (37.5%)		

Respiratory System				
			Pharyngitis (6.3%)	
				Sinusitis (3.1%)
Skin and Appendages				
				Acne (3.1%)
			Dermatitis fungal (9.4%)	
				Nail disorder (3.1%)
			Pruritus (6.3%)	
		Rash (25.0%)		
			Rash maculo-papular (18.7%)	
			Sweating (12.5%)	
Urogenital System				
				Albuminuria (3.1%)

8.2.7.1 Additional adverse events, regardless of causality

Body as a whole: Abdomen enlarged, fever, photosensitivity, upper extremity pain.
Cardiovascular System: Bradycardia, hypertension, hypotension, peripheral vascular disorder, tachycardia, vasodilation.
Digestive system: Anorexia, appetite increase/weight gain, dry mouth, dyspepsia, enlarged liver, eructation, flatulence, increased liver function tests, intestinal obstruction, vomiting.
Hemic and lymphatic: ESR decrease, eosinophilia, granulocytopenia, hypochromic anemia, leukemia, leukocytosis, leukopenia, MCV elevated, RBC abnormal, spleen palpable, thrombocytopenia.
Metabolic and endocrine: ADH inappropriate, amyloidosis, bilirubinemia, BUN increased, creatinine increased, cyanosis, diabetes, edema, electrolyte abnormalities, hyperglycemia, hyperkalemia, hyperuricemia, hypocalcemia, hypoproteinemia, LDH increased, phosphorus decreased, ALT increased.
Muscular skeletal: Arthritis, bone tenderness, hypertonia, joint disorder, leg cramps, myalgia, myasthenia, periosteal disorder.
Nervous system: Abnormal thinking, agitation, amnesia, anxiety, causalgia, circumoral paresthesia, confusion, depression, euphoria, hyperesthesia, insomnia, nervousness, neuralgia, neuritis, neuropathy, paresthesia, peripheral neuritis, psychosis.
Respiratory system: Cough, emphysema, epistaxis, pulmonary embolus, rales, upper respiratory infection, voice alteration.
Skin and appendages: Acne, alopecia, dry skin, eczematous rash, exfoliative dermatitis, ichthyosis, perifollicular thickening, skin necrosis, seborrhea, sweating, urticaria, vesiculobullous rash.
Special senses: Amblyopia, deafness, dry eye, eye pain, tinnitus.
Urogenital: Decreased creatinine clearance, hematuria, orchitis, proteinuria, pyuria, urinary frequency.

8.2.8 Method of Procurement

Thalidomide is commercially available and will be prelabeled by pharmacy as per institutional standards (according to national law). The drug will be handed to the patient by a designated person. Drug Batch Numbers and expiry date will be documented in the study drug accountability log. The returned blister packs will be checked by the study monitor and thereafter disposed of as per local practice. Patients, prescribers and dispensing pharmacies in the USA must be registered in the FDA-mandated S.T.E.P.S.® program.

8.3 Celecoxib

8.3.1 Description
The empirical formula for celecoxib is $C_{17}H_{14}F_3N_3O_2S$, and the molecular weight is 381.38 g/mol.

8.3.2 Formulation
Celecoxib oral capsules contain 100 mg and 200 mg of celecoxib. The inactive ingredients in celecoxib capsules include: croscarmellose sodium, edible inks, gelatin, lactose monohydrate, magnesium stearate, povidone, sodium lauryl sulfate and titanium dioxide.

8.3.3 Reconstitution
None.

8.3.4 Application
Celecoxib is taken by mouth twice daily. Celecoxib is initiated at 100 mg po bid (morning and prior to bed) in patients 10-35 kg. Patients <10 kg receive 50 mg po bid (magistral formula). Patients >20 kg can increase the dose to 200 mg po bid as tolerated. Patients >35 kg can increase the dose to 300 mg po bid as tolerated. Patients >50 kg can escalate the dose to 400 mg po bid as tolerated. Dose escalations can be initiated every one to two weeks.

8.3.5 Pharmacology
Absorption: Peak plasma levels of celecoxib occur approximately 3 hrs after an oral dose. Under fasting conditions, both peak plasma levels (C_{max}) and area under the curve (AUC) are roughly dose proportional up to 200 mg BID; at higher doses there are less than proportional changes in C max and AUC. Absolute bioavailability studies have not been conducted. With multiple dosing, steady state conditions are reached on or before day 5 (Davies, 2000).

8.3.6 Distribution
In healthy subjects, celecoxib is highly protein bound (~97%) within the clinical dose range. In vitro studies indicate that celecoxib binds primarily to albumin and, to a lesser extent, (alpha) 1 -acid glycoprotein. The apparent volume of distribution at steady state (V_{ss}/F) is approximately 400 L, suggesting extensive distribution into the tissues. Celecoxib is not preferentially bound to red blood cells.

8.3.7 Metabolism
Celecoxib metabolism is primarily mediated via cytochrome P450 2C9.
Three metabolites, a primary alcohol, the corresponding carboxylic acid and its glucuronide conjugate, have been identified in human plasma. These metabolites are inactive as COX-1 or COX-2 inhibitors. Patients who are known or suspected to be P450 2C9 poor metabolizers based on a previous history should be administered celecoxib with caution as they may have abnormally high plasma levels due to reduced metabolic clearance.

8.3.8 Excretion
Celecoxib is eliminated predominantly by hepatic metabolism with little (<3%) unchanged drug recovered in the urine and feces. Following a single oral dose of radiolabeled drug, approximately 57% of the dose was excreted in the feces and 27% was excreted into the urine. The primary metabolite in both urine and feces was the carboxylic acid metabolite (73% of dose) with low amounts of the glucuronide also appearing in the urine. It appears that the low solubility of the drug prolongs the absorption process making terminal half-life (t ½) determinations more variable. The effective half-life is approximately 11 hours under fasted conditions. The apparent plasma clearance is about 500 mL/min. The rate of absorption of celecoxib is moderate when given orally (peak plasma drug concentration occurs after 2 to 4

hours), although the extent of absorption is not known. Celexocib is extensively protein bound, primarily to plasma albumin, and has an apparent volume of distribution of 455+/-166L in humans. Racial differences in drug disposition and pharmacokinetic changes in the elderly have been reported for celecoxib. Plasma concentrations (AUC) of celecoxib appear to be 43% lower in patients with chronic renal insufficiency compared with individuals with healthy renal function, with a 47% increase in apparent clearance (Davies, 2000).
Compared with healthy controls, it has been reported that the steady-state AUC is increased by approximately 40% and 180% in patients with mild and moderate hepatic impairment, respectively. Celecoxib does not appear to interact with warfarin, ketoconazole or methotrexate; however, clinically significant drug interactions with fluconazole and lithium have been documented. As celecoxib is metabolized by CYP2C9, increased clinical vigilance is required during the co-administration of other substrates or inhibitors of this enzyme.

8.3.9 Dosing in hepatic disease
A pharmacokinetic study in subjects with mild (Child-Pugh Class I) and moderate (Child-Pugh Class II) hepatic impairment has shown that steady-state celecoxib AUC is increased about 40% and 180%, respectively, above that seen in healthy control subjects. Therefore, the daily-recommended dose of celecoxib capsules should be reduced by approximately 50% in patients with moderate (Child-Pugh Class II) hepatic impairment. Patients with severe hepatic impairment have not been studied. The use of celecoxib in patients with severe hepatic impairment is not recommended.

8.3.10 Renal Insufficiency
In a cross-study comparison, celecoxib AUC was approximately 40% lower in patients with chronic renal insufficiency than that seen in subjects with normal renal function. No significant relationship was found between GFR and celecoxib clearance. Patients with severe renal insufficiency have not been studied.

8.3.11 Drug Interactions
Significant interactions may occur when celecoxib is administered together with drugs that inhibit P450 2C9. In vitro studies indicate that celecoxib is not an inhibitor of cytochrome P450 2C9, 2C19 or 3A4. Clinical studies with celecoxib have identified potentially significant interactions with fluconazole and lithium. Experience with nonsteroidal anti-inflammatory drugs (NSAIDs) suggests the potential for interactions with furosemide and ACE inhibitors. The effects of celecoxib on the pharmacokinetics and/or pharmacodynamics of glyburide, ketoconazole, methotrexate, phenytoin, and tolbutamide have been studied in vivo and clinically important interactions have not been found.

8.3.12 Toxicity
Of the celecoxib treated patients in controlled trials, approximately 4,250 were patients with osteoarthritis, approximately 2,100 were patients with rheumatoid arthritis, and approximately 1,050 were patients with post-surgical pain. More than 8,500 patients have received a total daily dose of celecoxib of 200 mg (100 mg BID or 200 mg QD) or more, including more than 400 treated at 800 mg (400 mg BID). Approximately 3,900 patients have received celecoxib at these doses for 6 months or more; approximately 2,300 of these have received it for 1 year or more and 124 of these have received it for 2 years or more.
In placebo- or active-controlled clinical trials, the discontinuation rate due to adverse events was 7.1% for patients receiving celecoxib and 6.1% for patients receiving placebo. Among the most common reasons for discontinuation due to adverse events in the celecoxib treatment groups were dyspepsia and abdominal pain (cited as reasons for discontinuation in 0.8% and 0.7% of celecoxib patients, respectively). Among patients receiving placebo, 0.6% discontinued due to dyspepsia and 0.6% withdrew due to abdominal pain. The following adverse events occurred in <0.1-1.9% of patients regardless of causality.

8.3.12.1 Gastrointestinal

Constipation, diverticulitis, dysphagia, eructation, esophagitis, gastritis, gastroenteritis, gastroesophageal reflux, hemorrhoids, hiatal hernia, melena, dry mouth, stomatitis, tenesmus, tooth disorder, vomiting, hepatic function abnormal, AST increased, ALT increased; intestinal obstruction, intestinal perforation, gastrointestinal bleeding, colitis with bleeding, esophageal perforation, pancreatitis, cholelithiasis, ileus, cholelithiasis, hepatitis, jaundice, liver failure. Patients treated on this protocol may be placed on a proton pump inhibitor or H2 blocker for GI bleed prophylaxis.

8.3.12.2 Cardiovascular

Aggravated hypertension, angina pectoris, coronary artery disorder, myocardial infarction; syncope, congestive heart failure, ventricular fibrillation, pulmonary embolism, cerebrovascular accident, peripheral gangrene, thrombophlebitis, vasculitis, palpitation, tachycardia, increased risk (2.5-3.4x greater) of heart attack or stroke.

8.3.12.3 General

Allergy aggravated, allergic reaction, asthenia, chest pain, cyst NOS, edema generalized, face edema, fatigue, fever, hot flushes, influenza-like symptoms, pain, peripheral pain; sepsis, sudden death, anaphylactic reaction, angioedema, insomnia, sinusitis.

8.3.12.4 Resistance mechanism disorders

Herpes simplex, herpes zoster, infection bacterial, infection fungal, infection soft tissue, infection viral, candidiasis, moniliasis genital, otitis media.

8.3.12.5 Central, peripheral nervous system

Leg cramps, hypertonia, hypoesthesia, migraine, neuralgia, neuropathy, paresthesia, vertigo, deafness, ear abnormality, earache, tinnitus, anorexia, anxiety, appetite increased, depression, nervousness, somnolence, taste perversion, blurred vision, cataract, conjunctivitis, eye pain, glaucoma; ataxia, suicide.

8.3.12.6 Reproductive

Breast fibroadenosis, breast neoplasm, breast pain, dysmenorrhea, menstrual disorder, vaginal hemorrhage, vaginitis, prostatic disorder, birth defects.

8.3.12.7 Metabolic and nutritional

BUN increased, CPK increased, diabetes mellitus, hypercholesterolemia, hyperglycemia, hypokalemia, creatinine increased, alkaline phosphatase increased, weight increase; hypoglycemia.

8.3.12.8 Musculoskeletal

Arthralgia, arthrosis, bone disorder, fracture accidental, myalgia, neck stiffness, synovitis, tendonitis.

8.3.12.9 Blood

Anemia, ecchymosis, epistaxis, thrombocythemia; thrombocytopenia, agranulocytosis, aplastic anemia, pancytopenia, leukopenia.

8.3.12.10 Respiratory

Bronchitis, bronchospasm, bronchospasm aggravated, coughing, dyspnea, laryngitis, pneumonia.

8.3.12.11 Skin
Alopecia, dermatitis, nail disorder, photosensitivity reaction, pruritus, rash erythematous, rash maculopapular, skin disorder, skin dry, sweating increased, urticaria; erythemia multiforme, exfoliative dermatitis, Stevens-Johnson syndrome, toxic epidermal necrolysis.

8.3.12.12 Application site disorders
Cellulitis, dermatitis contact, skin nodule.

8.3.12.13 Genitourinary
Albuminuria, cystitis, dysuria, hematuria, micturition frequency, renal calculus, urinary incontinence, urinary tract infection; acute renal failure, interstitial nephritis.

8.3.13 Frequencies of Toxicities

	Common<br>Happens to 21-100 patients out of every 100	Occasional<br>Happens to 5-20 patients out of every 100	Rare<br>Happens to <5 patients out of every 100
Gastrointestinal			
			Abdominal Pain (4.1%)
		Diarrhea (5.6%)	
		Dyspepsia (8.8%)	
			Flatulence (2.2%)
			Nausea (3.5%)
Body as a Whole			
			Back Pain (2.8%)
			Peripheral Edema (2.1%)
			Injury-Accidental (2.9%)
Central and Peripheral Nervous system			
			Dizziness (2.0%)
		Headache (15.8%)	
Psychiatric			
			Insomnia (2.3%)
Respiratory			
			Pharyngitis (2.3%)
			Rhinitis (2.0%)
		Sinusitis (5.0%)	
		Upper Respiratory Infection (8.1%)	
Skin			
			Rash (2.2%)

8.3.13.1 Additional adverse events, regardless of causality
Gastrointestinal: Constipation, diverticulitis, dysphagia, eructation, esophagitis, gastritis, gastroenteritis, gastroesophageal reflux, hemorrhoids, hiatal hernia, melena, dry mouth, stomatitis, tenesmus, vomiting, intestinal obstruction, intestinal perforation, gastrointestinal bleeding, colitis with bleeding, esophageal perforation, pancreatitis, ileus
Cardiovascular: Aggravated hypertension, angina pectoris, coronary artery disorder, myocardial infarction, syncope, congestive heart failure, ventricular fibrillation, pulmonary

embolism, cerebrovascular accident, peripheral gangrene, thrombophlebitis, vasculitis, deep venous thrombosis
General: Allergy aggravated, allergic reaction, chest pain, cyst NOS, edema generalized, face edema, fatigue, fever, hot flushes, influenza-like symptoms, pain, peripheral pain, sepsis, sudden death, anaphylactoid reaction, angioedema
Nervous system: Leg cramps, hypertonia, hypoesthesia, migraine, paresthesia, vertigo, ataxia, suicide, aseptic meningitis, ageusia, anosmia, fatal intracranial hemorrhage
Hearing and vestibular: Deafness, tinnitus
Heart rate and rhythm: Palpitation, tachycardia
Liver and biliary: Hepatic function abnormal, AST increased, ALT increased, cholelithiasis, hepatitis, jaundice, liver failure
Metabolic and nutritional: BUN increased, CPK increased, hypercholesterolemia, hyperglycemia, hypokalemia, creatinine increased, alkaline phosphatase increased, weight increased, hypoglycemia, hyponatremia
Musculoskeletal: Arthralgia, arthrosis, myalgia, synovitis, tendinitis
Platelets (bleeding clotting): Ecchymosis, epistaxis, thrombocythemia
Psychiatric: Anorexia, anxiety, appetite increased, depression, nervousness, somnolence
Hemic and lymphatic: Thrombocytopenia, agranulocytosis, aplastic anemia, pancytopenia, leucopenia
Respiratory: Bronchitis, bronchospasm, bronchospasm aggravated, coughing, dyspnea, laryngitis, pneumonia
Skin and appendages: Alopecia, dermatitis, photosensitivity reaction, pruritus, rash erythematous, rash maculopapular, skin disorder, skin dry, sweating increased, urticaria, erythema multiforme, exfoliative dermatitis, Stevens-Johnson syndrome, toxic epidermal necrolysis
Application site disorders: Cellulitis, dermatitis
Renal: Acute renal failure, interstitial nephritis
Urinary: Albuminuria, cystitis, dysuria, hematuria, micturition frequency, renal calculus

8.3.14 Method of Procurement
Celecoxib is commercially available and will be prelabeled by pharmacy as per institutional standards (according to national law). Drug Batch Numbers and expiry date will be documented in the study drug accountability log. The returned blister packs will be checked by the study monitor and thereafter disposed of as per local practice.

8.4 Fenofibrate

8.4.1 Ingredients
The empirical formula of fenofibrate is $C_{20}H_{21}O_4C_1$ and the molecular weight is 360.83 g/mol. Fenofibrate is insoluble in water. The melting point is 79-82°C. Fenofibrate is a white solid which is stable under ordinary conditions.

8.4.2 Formulation
The drug is available in two forms, tablets and capsules containing 43, 48, 67, 87, 130, 134, 145, 160, and 200 mg of drug depending on the commercial source. For specific brand and strength availability, refer to a pharmaceutical compendium. Micronized Fenofibrate is administered once daily.

8.4.3 Reconstitution
None.

8.4.4 Application
Fenofibrate will be given at an average daily oral dose of approximately 90 mg/m^2. This will be taken once daily with food to a maximum dose of 200 mg per day.

8.4.5 Pharmacology

Fenofibrate, a pro-drug, is rapidly and completely converted to its active form, fenofibric acid by hydrolysis. Elimination of the drug is similar for both the micronized and non-micronized forms. 90% of the micronized form of the drug is absorbed when taken with meals. It is protein bound, and reaches steady state levels in approximately 5 days. It is largely eliminated via the kidney with an elimination half-life of 20-22 hours. In patients with normal renal function, drug accumulation does not occur. Elderly patients can exhibit an increased time to drug excretion, as can patients with renal failure. The drug does not impact the P450 cytochrome system. Fenofibrate is insoluble in aqueous media required for injection although it is well absorbed from the gastrointestinal tract. In healthy volunteers, oral administration resulted in approximately 60% of a single dose of radiolabeled fenofibrate appearing in urine, primarily as fenofibric acid and its glucuronate conjugate, while 25% was excreted in the feces. Peak plasma levels occur within 6 to 8 hours after administration of fenofibric acid. Absorption of fenofibrate is increased when administered with food by 35% when compared to fasting conditions. Steady-state plasma levels are achieved within 5 days of dosing and do not demonstrate accumulation across time following multiple dose administration in normal volunteers. Approximately 99% of the drug is protein bound.

Fenofibric acid is eliminated with a T ½ of 20 hours, allowing once daily administration. Fenofibrate has been associated with increases in serum transaminases; AST and/or ALT. Increases to >3 times the upper limit of normal occurred in 5.3% of patients taking fenofibrate versus 1.1% of patients treated with placebo. When transaminase determinations were followed either after discontinuation of treatment or during continued treatment, a return to normal limits was usually observed.

8.4.5.1 Cholelithiasis

Fenofibrate may increase cholesterol excretion into the bile leading to cholelithiasis. If cholelithiasis is suspected, gallbladder studies are indicated. Continued fenofibrate therapy should be discussed with the Principal Investigator if gallstones are found.

8.4.5.2 Concomitant Oral Anticoagulants

Caution should be exercised when anticoagulants are given in conjunction with fenofibrate because of the potentiation of coumarin-type anticoagulants in prolonging the prothrombin time / INR.

8.4.5.3 Concomitant HMG-CoA Reductase Inhibitors

The combined use of fenofibrate and 3-hydroxy-3-methylglutaryl-coenzyme A (HMG-CoA) reductase inhibitors should be avoided. The combined use of fibric acid derivatives and HMG-CoA reductase inhibitors has been associated, in the absence of a marked pharmacokinetic interaction, in numerous case reports, with rhabdomyolysis, markedly elevated creatine phosphokinase (CPK) levels and myoglobinuria, leading in a high proportion of cases to acute renal failure. The use of fibrates alone, including fenofibrate, may occasionally be associated with myositis, myopathy, or rhabdomyolysis. Patients receiving fenofibrate and complaining of muscle pain, tenderness, or weakness should have prompt medical evaluation for myopathy, including serum creatine kinase level determination.

8.4.5.4 Pancreatitis

Pancreatitis has been reported in patients taking fenofibrate and may represent a failure of efficacy in patients with severe hypertriglyceridemia, a direct drug effect, or a secondary phenomenon mediated through biliary tract stone or sludge formation with obstruction of the common bile duct.

8.4.5.5 Hypersensitivity Reactions

Acute hypersensitivity reactions including severe skin rashes requiring patient hospitalization and treatment with steroids have occurred very rarely during treatment with fenofibrate, including rare spontaneous reports of Stevens-Johnson syndrome, and toxic epidermal necrolysis. Urticaria was seen in 1.1 vs 0%, and rash in 1.4 vs 0.8% of fenofibrate and placebo patients respectively in controlled trials.

8.4.5.6 Hematologic Changes

Mild to moderate hemoglobin, hematocrit, and white blood cell decreases have been observed in patients following initiation of fenofibrate therapy. However, these levels stabilize during long-term administration. Extremely rare spontaneous reports of thrombocytopenia and agranulocytosis have been received during post-marketing surveillance.

8.4.5.7 Skeletal muscle

The use of fibrates alone, including fenofibrate, may occasionally be associated with myopathy. Treatment with drugs of the fibrate class has been associated on rare occasions with rhabdomyolysis, usually in patients with impaired renal function. Myopathy should be considered in any patient with diffuse myalgias, muscle tenderness or weakness, and/or marked elevations of creatine phosphokinase levels. Patients should be advised to report promptly unexplained muscle pain, tenderness or weakness, particularly if accompanied by malaise or fever. CPK levels should be assessed in patients reporting these symptoms, and continued fenofibrate therapy should be discussed with the study Principal Investigator if markedly elevated CPK levels occur or myopathy is diagnosed.

8.4.6 Drug Interactions

8.4.6.1 Oral Anticoagulants

Caution should be exercised when coumarin anticoagulants are given in conjunction with fenofibrate. The dosage of the anticoagulants should be reduced to maintain the prothrombin time / INR at the desired level to prevent bleeding complications. Frequent prothrombin time / INR determinations are advisable until it has been definitely determined that the prothrombin time / INR has stabilized.

Fenofibrate has been demonstrated to be devoid of mutagenic potential in the following tests: Ames test, mouse lymphoma, chromosomal aberration and unscheduled DNA synthesis.

8.4.7 Toxicity

8.4.7.1 Clinical

Adverse events reported by 2% or more of patients treated with fenofibrate during the double-blind, placebo-controlled trials, regardless of causality, are listed in the table below. Adverse events led to discontinuation of treatment in 5.0% of patients treated with fenofibrate and in 3.0% treated with placebo. Increases in liver function tests were the most frequent events, causing discontinuation of fenofibrate treatment in 1.6% of patients in double-blind trials.

Additional adverse events reported by three or more patients in placebo-controlled trials or reported in other controlled or open trials, regardless of causality are listed below.

8.4.7.2 Body as a whole

Chest pain, pain (unspecified), infection, malaise, allergic reaction, cyst, hernia, fever, photosensitivity reaction, and accidental injury.

8.4.7.3 Cardiovascular

Angina pectoris, hypertension, vasodilatation, coronary artery disorder, abnormal electrocardiogram, ventricular extrasystoles, myocardial infarct, migraine, varicose vein, cardiovascular disorder, hypotension, palpitation, vascular disorder, arrhythmia, phlebitis, tachycardia, extrasystoles, and atrial fibrillation.

8.4.7.4 Digestive
Dyspepsia, flatulence, nausea, increased appetite, gastroenteritis, cholelithiasis, rectal disorder, esophagitis, gastritis, colitis, tooth disorder, vomiting, anorexia, gastrointestinal disorder, duodenal ulcer, nausea and vomiting, peptic ulcer, rectal hemorrhage, liver fatty deposit, cholecystitis, eructation, gamma glutamyl transpeptidase, and diarrhea.

8.4.7.5 Endocrine
Diabetes mellitus

8.4.7.6 Hemic and lymphatic
Anemia, leukopenia, ecchymosis, eosinophilia, lymphadenopathy, and thrombocytopenia.

8.4.7.7 Metabolic and nutritional disorders
Creatinine increased, weight gain, hypoglycemia, gout, weight loss, edema, hyperuricemia, and peripheral edema.

8.4.7.8 Musculoskeletal
Myositis, myalgia, arthralgia, arthritis, tenosynovitis, joint disorder, arthrosis, leg cramps, bursitis, and myasthenia.

8.4.7.9 Nervous system
Dizziness, insomnia, depression, vertigo, libido decreased, anxiety, paresthesia, dry mouth, hypertonia, nervousness, neuralgia, and somnolence.

8.4.7.10 Respiratory
Pharyngitis, bronchitis, cough increased, dyspnea, asthma, pneumonia, laryngitis, and sinusitis.

8.4.7.11 Skin and appendages
Rash, pruritus, eczema, herpes zoster, urticaria, acne, sweating, fungal dermatitis, skin disorder, alopecia, contact dermatitis, herpes simplex, maculopapular rash, nail disorder, and skin ulcer.

8.4.7.12 Special Senses
Conjunctivitis, eye disorder, amblyopia, ear pain, otitis media, abnormal vision, cataract specified, and refraction disorder.

8.4.7.13 Urogenital System
Urinary frequency, prostatic disorder, dysuria, kidney function abnormal, urolithiasis, gynecomastia, unintended pregnancy, vaginal moniliasis, and cystitis.

8.4.8 Frequencies of Toxicities

		Common Happens to 21-100 patients out of every 100	Occasional Happens to 5-20 patients out of every 100	Rare Happens to <5 patients out of every 100
Body as a Whole				
				Abdominal Pain (4.6%)
				Back Pain (3.4%)

				Headache (3.2%)
				Asthenia (2.1%)
				Flu Syndrome (2.1%)
Digestive				
			Liver Function Tests Abnormal (7.5%)	
				Diarrhea (2.3%)
				Nausea (2.3%)
				Constipation (2.1%)
Metabolic and nutritional disorders				
				ALT Increased (3.0%)
				Creatine Phosphokinase Increased (3.0%)
				AST Increased (3.4%)
Respiratory				
			Respiratory Disorder (6.2%)	
				Rhinitis (2.3%)

8.4.8.1 Additional adverse events, regardless of causality

Body as a whole: Accidental injury, allergic reaction, chest pain, cyst, fever, hernia, infection, malaise and pain (unspecified).

Cardiovascular system: Angina pectoris, arrhythmia, atrial fibrillation, cardiovascular disorder, coronary artery disorder, electrocardiogram abnormal, extrasystoles, hypertension, hypotension, migraine, myocardial infarct, palpitation, peripheral vascular disorder, phlebitis, tachycardia, varicose vein, vascular disorder, vasodilatation, venous thromboembolic events (deep vein thrombosis, pulmonary embolus) and ventricular extrasystoles.

Digestive system: Anorexia, cholecystitis, cholelithiasis, colitis, diarrhea, duodenal ulcer, dyspepsia, eructation, esophagitis, flatulence, gastritis, gastroenteritis, gastrointestinal disorder, increased appetite, jaundice, liver fatty deposit, nausea, pancreatitis, peptic ulcer, rectal disorder, rectal hemorrhage, tooth disorder and vomiting.

Endocrine system: Diabetes mellitus.

Hemic and lymphatic system: Anemia, ecchymosis, eosinophilia, leukopenia, lymphadenopathy, and thrombocytopenia.

Laboratory investigations: Alkaline phosphatase increased, bilirubin increased, blood urea nitrogen increased, serum creatinine increased, gamma glutamyl transpeptidase increased, lactate dehydrogenase increased, AST and ALT increased.

Metabolic and nutritional disorders: Edema, gout, hyperuricemia, hypoglycemia, peripheral edema, weight gain, and weight loss.
Musculoskeletal system: Arthralgia, arthritis, arthrosis, bursitis, joint disorder, leg cramps, myalgia, myasthenia, myositis, rhabdomyolysis and tenosynovitis.
Nervous system: Anxiety or nervousness, depression, dizziness, dry mouth, hypertonia, insomnia, libido decreased, neuralgia, paresthesia, somnolence and vertigo.
Respiratory system: Allergic pulmonary alveolitis, asthma, bronchitis, cough increased, dyspnea, laryngitis, pharyngitis, pneumonia and sinusitis.
Skin and appendages: Acne, alopecia, contact dermatitis, eczema, fungal dermatitis, herpes simplex, herpes zoster, maculopapular rash, nail disorder, photosensitivity reaction, pruritus, rash, sweating, skin disorder, skin ulcer and urticaria.
Special senses: Abnormal vision, amblyopia, cataract specified, conjunctivitis, ear pain, eye disorder, otitis media and refraction disorder.
Urogenital system: Abnormal kidney function, cystitis, dysuria, gynecomastia, prostatic disorder, unintended pregnancy, urinary frequency, urolithiasis and vaginal candidiasis.

8.4.9 Method of Procurement
Fenofibrate is commercially available and will be prelabeled by pharmacy as per institutional standards (according to national law). Drug Batch Numbers and expiry date will be documented in the study drug accountability log. The returned blister packs will be checked by the study monitor and thereafter disposed of as per local practice.

8.5 Etoposide

8.5.1 Description
Etoposide (also commonly known as VP-16) is a semisynthetic derivative of podophyllotoxin used in the treatment of certain neoplastic diseases. It is 4'- demethylepipodophyllotoxin 9-[4,6-0-(R)-ethylidene-(beta)-D-glucopyranoside]. It is very soluble in methanol and chloroform, slightly soluble in ethanol, and sparingly soluble in water and ether. It is made more miscible with water by means of organic solvents. It has a molecular weight of 588.58 g/mol and a molecular formula of $C_{29}H_{32}O_{13}$.

8.5.2 Formulation
Etoposide for oral use can be made from the injectable form. It is available in 100 mg (5 mL), 150 mg (7.5 mL), 500 mg (25 mL), or 1 gram (50 mL), sterile, multiple dose vials. The pH of the clear, nearly colorless to yellow liquid is 3 to 4. Each mL contains 20 mg etoposide, 2 mg citric acid, 30 mg benzyl alcohol, 80 mg modified polysorbate 80/tween 80, 650 mg polyethylene glycol 300, and 30.5 percent (v/v) alcohol. Vial headspace contains nitrogen. Etoposide is also available as 25 and 50 mg capsules. Each liquid filled, soft gelatin capsule contains etoposide in a vehicle consisting of citric acid, glycerin, purified water, polyethylene glycol 400, gelatin, sorbitol, and parabens (ethyl and propyl) with the following dye system: iron oxide (red) and titanium dioxide; the capsules are printed with edible ink.

8.5.3 Reconstitution
A liquid form of oral etoposide can be prepared from the IV formulation as per institutional guidelines.

8.5.4 Application
Etoposide will be given at an average daily oral dose of approximately 50 mg/m^2 (in the morning or prior to bed). Etoposide for oral use can be made from the injectable form. Patients will take the drug orally once daily for 21 consecutive days. This medication will alternate with oral cyclophosphamide.

8.5.5 Pharmacology

On intravenous administration, the disposition of etoposide is best described as a biphasic process with a distribution half-life of about 1.5 hours and terminal elimination half-life ranging from 4 to 11 hours. Total body clearance values range from 33 to 48 mL/min or 16 to 36 mL/min/m^2 and, like the terminal elimination half-life, are independent of dose over a range 100-600 mg/m^2. Over the same dose range, the areas under the plasma concentration vs. time curves (AUC) and the maximum plasma concentration (C_{max}) values increase linearly with dose. Etoposide does not accumulate in the plasma following daily administration of 100 mg/m^2 for 4 to 5 days.

The mean volumes of distribution at steady state fall in the range of 18 to 29 liters or 7 to 17 L/m^2. Etoposide enters the CSF poorly. Although it is detectable in CSF and intracerebral tumors, the concentrations are lower than in extracerebral tumors and in plasma. *In vitro*, etoposide is highly protein bound (97%) to human plasma proteins. An inverse relationship between plasma albumin levels and etoposide renal clearance is found in children. In a study determining the effect of other therapeutic agents on the *in vitro* binding of carbon-14 labeled etoposide to human serum proteins, only phenylbutazone, sodium salicylate, and aspirin displaced protein-bound etoposide at concentrations achieved *in vivo*.

The etoposide binding ratio correlates directly with serum albumin in patients with cancer and in normal volunteers. The unbound fraction of etoposide significantly correlated with bilirubin in a population of cancer patients. Data have suggested a significant inverse correlation between serum albumin concentration and free fraction of etoposide. After intravenous administration of 3 H-etoposide (70-290 mg/m^2), mean recoveries of radioactivity in the urine range from 42 to 67%, and fecal recoveries range from 0 to 16% of the dose. Less than 50% of an intravenous dose is excreted in the urine as etoposide with mean recoveries of 8 to 35% within 24 hours. In children, approximately 55% of the dose is excreted in the urine as etoposide in 24 hours. The mean renal clearance of etoposide is 7 to 10 mL/min/m^2 or about 35% of the total body clearance over a dose range of 80 to 600 mg/m^2. Etoposide, therefore, is cleared by both renal and nonrenal processes, i.e., metabolism and biliary excretion. The effect of renal disease on plasma etoposide clearance is not known. Biliary excretion appears to be a minor route of etoposide elimination. Only 6% or less of an intravenous dose is recovered in the bile as etoposide. Metabolism accounts for most of the nonrenal clearance of etoposide.

The major urinary metabolite of etoposide in adults and children is the hydroxy acid [4'-demethylepipodophyllic acid-9-(4,6-0-(R)-ethylidene-(beta)-D-glucopyranoside)], formed by opening of the lactone ring. It is also present in human plasma, presumably as the trans isomer. Glucuronide and/or sulfate conjugates of etoposide are excreted in human urine and represent 5 to 22% of the dose. In addition, O-demethylation of the dimethoxyphenol ring occurs through the CYP450 3A4 isoenzyme pathway to produce the corresponding catechol. After either intravenous infusion or oral capsule administration, the C_{max} and AUC values exhibit marked intra- and inter-subject variability. This results in variability in the estimates of the absolute oral bioavailability of etoposide oral capsules. C_{max} and AUC values for orally administered etoposide capsules consistently fall in the same range as the C_{max} and AUC values for an intravenous dose of one-half the size of the oral dose. The overall mean value of oral capsule bioavailability is approximately 50% (range 25-75%). The bioavailability of etoposide capsules appears to be linear up to a dose of at least 250 mg/m^2. There is no evidence of a first-pass effect for etoposide. For example, no correlation exists between the absolute oral bioavailability of etoposide capsules and nonrenal clearance. No evidence exists for any other differences in etoposide metabolism and excretion after administration of oral capsules as compared to intravenous infusion. In adults, the total body clearance of etoposide is correlated with creatinine clearance, serum albumin concentration, and nonrenal clearance. Patients with impaired renal function receiving etoposide have exhibited reduced total body clearance, increased AUC and a lower volume of distribution at steady state. In children, elevated serum ALT levels are associated with reduced drug total body clearance. Prior use of

cisplatin may also result in a decrease of etoposide total body clearance in children. Although some minor differences in pharmacokinetic parameters between age and gender have been observed, these differences were not considered clinically significant.

8.5.6 Toxicity

8.5.6.1 Hematologic Toxicity

Myelosuppression is dose related and dose limiting with the IV schedule, with granulocyte nadirs occurring 7 to 14 days after drug administration and platelet nadirs occurring 9 to 16 days after drug administration. Bone marrow recovery is usually complete by day 20, and no cumulative toxicity has been reported. Fever and infection have also been reported in patients with neutropenia. Death associated with myelosuppression has been reported. The occurrence of acute leukemia with or without a preleukemic phase has been reported rarely in patients treated with etoposide in association with other antineoplastic agents. Myelosuppression is less prominent with oral administration.

8.5.6.2 Gastrointestinal

Nausea and vomiting are the major gastrointestinal toxicities. The severity of such nausea and vomiting is generally mild to moderate with treatment discontinuation required in 1% of patients. Nausea and vomiting can usually be controlled with standard antiemetic therapy. Mild to severe mucositis / esophagitis may occur. In rare cases some patients have had diarrhea. Gastrointestinal toxicities are slightly more frequent after oral administration than after intravenous infusion.

8.5.6.3 Hypotension

Transient hypotension following rapid intravenous administration has been reported in 1% to 2% of patients. It has not been associated with cardiac toxicity or electrocardiographic changes. This has not been observed with the oral form. No delayed hypotension has been noted.

8.5.6.4 Allergic Reactions

Anaphylactic-like reactions characterized by chills, fever, tachycardia, bronchospasm, dyspnea, and / or hypotension have been reported to occur in 0.7% to 2% of patients receiving intravenous etoposide and in less than 1% of the patients treated with the oral capsules. These reactions have usually responded promptly to the cessation of the infusion and administration of pressor agents, corticosteroids, antihistamines, or volume expanders as appropriate; however, the reactions can be fatal. Hypertension and/or flushing have also been reported. Blood pressure usually normalizes within a few hours after cessation of the infusion. Anaphylactic-like reactions have occurred during the initial infusion of etoposide. Facial/tongue swelling, coughing, diaphoresis, cyanosis, tightness in throat, laryngospasm, back pain, and/or loss of consciousness have sometimes occurred in association with the above reactions. In addition, an apparent hypersensitivity-associated apnea has been reported rarely. Rash, urticaria, and/or pruritus have infrequently been reported at recommended doses. At investigational doses, a generalized pruritic erythematous maculopapular rash, consistent with perivasculitis, has been reported.

8.5.6.5 Alopecia

Reversible alopecia, sometimes progressing to total baldness, was observed in up to 66% of patients. Patients on the oral form can also demonstrate alopecia, although it may be less severe than that reported for the intravenous form.

8.5.7 Frequencies of Toxicities

	Common Happens to 21-100 patients out of every 100	Occasional Happens to 5-20 patients out of every 100	Rare Happens to <5 patients out of every 100
Hematologic toxicity			
		Leukopenia (less than 1,000 WBC/mm^3) (3-17%)	
	Leukopenia (less than 4,000 WBC/mm^3) (60-91%)		
		Thrombocytopenia (less than 50,000 platelets/mm^3) (1-20%)	
	Thrombocytopenia (less than 100,000 platelets/mm^3) (22-41%)		
	Anemia (0-33%)		
Gastrointestinal toxicity			
	Nausea and vomiting (31-43%)		
			Abdominal pain (0-2%)
		Anorexia (10-13%)	
		Diarrhea (1-13%)	
		Stomatitis (1-6%)	
			Hepatic (0-3%)
Nervous System Disorders			
			Peripheral neurotoxicity (1-2%)
Body as a Whole			
	Alopecia (8-66%)		
			Hypotension (1-2%)
			Allergic reaction (1-2%)

8.5.7.1 Additional adverse events, regardless of causality

The following adverse reactions have been infrequently reported with either the oral or IV form of etoposide: abdominal pain, aftertaste, constipation, dysphagia, asthenia, fatigue, malaise, somnolence, transient cortical blindness, optic neuritis, interstitial

pneumonitis/pulmonary fibrosis, fever, seizure (occasionally associated with allergic reactions), severe infection that may be serious or life-threatening, Stevens-Johnson syndrome, and toxic epidermal necrolysis, pigmentation, and a single report of radiation recall dermatitis. Hepatic toxicity, generally in patients receiving higher doses of the drug than those recommended, has been reported with etoposide. Metabolic acidosis has also been reported in patients receiving higher doses.

8.5.8 Method of Procurement
Etoposide is commercially available and will be prelabeled by pharmacy as per institutional standards (according to national law). Drug Batch Numbers and expiry date will be documented in the study drug accountability log. The returned blister packs or vials will be checked by the study monitor and thereafter disposed of as per local practice.

8.6 Cyclophosphamide

8.6.1 Description
Cyclophosphamide is a synthetic antineoplastic drug chemically related to the nitrogen mustards. Cyclophosphamide is a white crystalline powder with the molecular formula of $C_7H_{15}Cl_2N_2O_2$ P•H_2O and a molecular weight of 279.1. The chemical name for cyclophosphamide is 2-[bis(2-chloroethyl)amino]tetrahydro-2H-1,3,2- oxazaphosphorine 2-oxide monohydrate. Cyclophosphamide is soluble in water, saline, or ethanol.

8.6.2 Formulation
Cyclophosphamide for oral use can be made from the injectable form. Lyophilized cyclophosphamide for injection is a sterile white lyophilized cake, or partially broken cake, containing 75 mg mannitol per 100 mg cyclophosphamide (anhydrous). Cyclophosphamide tablets are for oral use and contain 25 mg or 50 mg cyclophosphamide (anhydrous). Inactive ingredients in cyclophosphamide tablets are: acacia, FD&C Blue No. 1, D&C Yellow No. 10 Aluminum Lake, lactose, magnesium stearate, starch, stearic acid, and talc.

8.6.3 Reconstitution
A liquid form of oral cyclophosphamide can be prepared from the IV formulation as per institutional guidelines.

8.6.4 Application
Cyclophosphamide should be given at an average daily dose of approximately 2.5 mg/kg/day or 100 mg po qam (quaque ante meridiem - "every morning"), whichever is lower. A liquid form of oral cyclophosphamide can be prepared from the IV formulation. Patients will take the drug orally once daily for 21 consecutive days. This medication will alternate with oral etoposide.

8.6.5 Pharmacology
Cyclophosphamide is biotransformed principally in the liver to active alkylating metabolites by a mixed function microsomal oxidase system.
These metabolites interfere with the growth of susceptible rapidly proliferating malignant cells. The mechanism of action is thought to involve cross-linking of tumor cell DNA. Cyclophosphamide is well absorbed after oral administration with a bioavailability greater than 75%. The unchanged drug has an elimination half-life of 3 to 12 hours. It is eliminated primarily in the form of metabolites, but from 5 to 25% of the dose is excreted in urine as unchanged drug. Several cytotoxic and noncytotoxic metabolites have been identified in urine and in plasma. Concentrations of metabolites reach a maximum in plasma 2 to 3 hours after an intravenous dose. Plasma protein binding of unchanged drug is low but some metabolites are bound to an extent greater than 60%. It has not been demonstrated that any single metabolite is responsible for either the therapeutic or toxic effects of cyclophosphamide.

Although elevated levels of metabolites of cyclophosphamide have been observed in patients with renal failure, increased clinical toxicity in such patients has not been demonstrated.

8.6.6 Toxicity

8.6.6.1 Carcinogenesis, Mutagenesis, Impairment of Fertility

Second malignancies have developed in some patients treated with cyclophosphamide used alone or in association with other antineoplastic drugs and/or modalities. Most frequently, they have been urinary bladder, myeloproliferative, or lymphoproliferative malignancies. Second malignancies most frequently were detected in patients treated for primary myeloproliferative or lymphoproliferative malignancies or nonmalignant disease in which immune processes are believed to be involved pathologically. In some cases, the second malignancy developed several years after cyclophosphamide treatment had been discontinued. In a single breast cancer trial utilizing two to four times the standard dose of cyclophosphamide in conjunction with doxorubicin, a small number of cases of secondary acute myeloid leukemia occurred within two years of treatment initiation. Urinary bladder malignancies generally have occurred in patients who previously had hemorrhagic cystitis. In patients treated with cyclophosphamide-containing regimens for a variety of solid tumors, isolated case reports of secondary malignancies have been published. One case of carcinoma of the renal pelvis was reported in a patient receiving long-term cyclophosphamide therapy for cerebral vasculitis. Cyclophosphamide can cause fetal harm when administered to a pregnant woman and such abnormalities have been reported following cyclophosphamide therapy in pregnant women. Abnormalities were found in two infants and a 6-month-old fetus born to women treated with cyclophosphamide. Ectrodactylia was found in two of the three cases. Normal infants have also been born to women treated with cyclophosphamide during pregnancy, including the first trimester. If this drug is used during pregnancy, or if the patient becomes pregnant while receiving this drug, the patient should be apprised of the potential hazard to the fetus. Women of childbearing potential should be advised to avoid becoming pregnant. Cyclophosphamide interferes with oogenesis and spermatogenesis. It may cause sterility in both sexes. Development of sterility appears to depend on the dose of cyclophosphamide, duration of therapy, and the state of gonadal function at the time of treatment. Cyclophosphamide induced sterility may be irreversible in some patients. Amenorrhea associated with decreased estrogen and increased gonadotropin secretion develops in a significant proportion of women treated with cyclophosphamide. Affected patients generally resume regular menses within a few months after cessation of therapy. Girls treated with cyclophosphamide during prepubescence generally develop secondary sexual characteristics normally and have regular menses. Ovarian fibrosis with apparently complete loss of germ cells after prolonged cyclophosphamide treatment in late prepubescence has been reported. Girls treated with cyclophosphamide during prepubescence subsequently have conceived. Men treated with cyclophosphamide may develop oligospermia or azoospermia associated with increased gonadotropin but normal testosterone secretion. Sexual potency and libido are unimpaired in these patients. Boys treated with cyclophosphamide during prepubescence develop secondary sexual characteristics normally, but may have oligospermia or azoospermia and increased gonadotropin secretion. Some degree of testicular atrophy may occur. Cyclophosphamide-induced azoospermia is reversible in some patients, though the reversibility may not occur for several years after cessation of therapy. Men temporarily rendered sterile by cyclophosphamide have subsequently fathered normal children.

8.6.6.2 Urinary System

Hemorrhagic cystitis may develop in patients treated with high doses of cyclophosphamide. Rarely, this condition can be severe and even fatal. Fibrosis of the urinary bladder, sometimes extensive, also may develop with or without accompanying cystitis. Atypical urinary bladder epithelial cells may appear in the urine. These adverse effects appear to depend on the dose of cyclophosphamide and the duration of therapy. Such bladder injury is thought to be due to cyclophosphamide metabolites excreted in the urine. Forced fluid intake helps to assure an

ample output of urine, necessitates frequent voiding, and reduces the time the drug remains in the bladder. This helps to prevent cystitis. Hematuria usually resolves in a few days after cyclophosphamide treatment is stopped, but it may persist. Medical and/or surgical supportive treatment may be required, rarely, to treat protracted cases of severe hemorrhagic cystitis. It is usually necessary to discontinue cyclophosphamide therapy in instances of severe hemorrhagic cystitis.

8.6.6.3 Cardiac Toxicity

Although a few instances of cardiac dysfunction have been reported following use of recommended doses of IV cyclophosphamide, no causal relationship has been established. Acute cardiac toxicity has been reported with doses as low as 2.4 g/m^2 to as high as 26 g/m^2, usually as a portion of an intensive antineoplastic multidrug regimen or in conjunction with transplantation procedures. In a few instances with high doses of cyclophosphamide, severe, and sometimes fatal, congestive heart failure has occurred after the first cyclophosphamide dose. Histopathologic examination has primarily shown hemorrhagic myocarditis. Hemopericardium has occurred secondary to hemorrhagic myocarditis and myocardial necrosis. Pericarditis has been reported independent of any hemopericardium. No residual cardiac abnormalities, as evidenced by electrocardiogram or echocardiogram appear to be present in patients surviving episodes of apparent cardiac toxicity associated with high doses of cyclophosphamide. Cyclophosphamide has been reported to potentiate doxorubicin-induced cardiotoxicity.

8.6.6.4 Infections

Treatment with cyclophosphamide may cause significant suppression of immune responses. Serious, sometimes fatal, infections may develop in severely immunosuppressed patients. Cyclophosphamide treatment may not be indicated or should be interrupted or the dose reduced in patients who have or who develop viral, bacterial, fungal, protozoan, or helminthic infections.

8.6.6.5 Other

Anaphylactic reactions have been reported; death has also been reported in association with this event. Possible cross-sensitivity with other alkylating agents has been reported. Nausea, vomiting and hair loss have also been reported.

8.6.7 Frequencies of Toxicities

	Common Happens to 21-100 patients out of every 100	Occasional Happens to 5-20 patients out of every 100	Rare Happens to <5 patients out of every 100
Hematologic toxicity			
		Neutropenia (8%)	
		Thrombocytopenia (13%)	
Body as a Whole			
			Hypokalemia (3%)
			Hypophosphatemia (3%)
Infection			
		Pneumonia (5%)	
			Varicella zoster

			(3%)
Gastrointestinal toxicity			
		Nausea and vomiting (8%)	
			Diarrhea (3%)
			Hepatic (3%)
Neurology			
		Peripheral neuropathy (8%)	

8.6.7.1 Additional adverse events, regardless of causality
Digestive system: Anorexia and, less frequently, abdominal discomfort or pain and diarrhea may occur. There are isolated reports of hemorrhagic colitis, oral mucosal ulceration and jaundice occurring during therapy.
Skin: Alopecia occurs commonly in patients treated with cyclophosphamide. Skin rash occurs occasionally in patients receiving the drug. Pigmentation of the skin and changes in nails can occur. Very rare reports of Stevens-Johnson syndrome and toxic epidermal necrolysis.
Urinary system: Hemorrhagic ureteritis and renal tubular necrosis have been reported to occur in patients treated with cyclophosphamide.
Other: Anaphylactic reactions have been reported, SIADH (syndrome of inappropriate ADH secretion) has been reported with the use of cyclophosphamide. Malaise and asthenia have been reported.

8.6.8 Method of Procurement
Cyclophosphamide is commercially available and will be prelabeled by pharmacy as per institutional standards (according to national law). Drug Batch Numbers and expiry date will be documented in the study drug accountability log. The returned blister packs or vials will be checked by the study monitor and thereafter disposed of as per local practice.

8.7 Intraventricular cytarabine

8.7.1 Description
Aqueous cytarabine is a sterile, injectable suspension of the antimetabolite cytarabine. Chemically, cytarabine is 4-amino-1 -β-D-arabinofuranosyl-2(1H)-pyrimidinone, also known as cytosine arabinoside ($C_9H_{13}N_3O_5$, molecular weight 243.22 g/mol).

8.7.2 Formulation
Aqueous cytarabine Injection is a sterile solution of cytarabine for intravenous, intrathecal or subcutaneous administration. Each mL contains 20 mg cytarabine in 100 mg (20 mg/mL) single dose vial and 100 mg cytarabine in 2 g (100 mg/mL) single dose vial.
Liposomal cytarabine 50 mg suspension for injection. Each 5 mL vial contains a white to off-white suspension of 50 mg cytarabine (10 mg/mL) for injection. Store in a refrigerator (2°C - 8°C). Do not freeze. Shelf life is 18 months.
Vials should be allowed to warm to room temperature (18°C -22°C) for a minimum of 30 minutes and be gently inverted to resuspend the particles immediately prior to withdrawal from the vial. Avoid vigorous shaking. No further reconstitution or dilution is required. Liposomal cytarabine should be withdrawn from the vial immediately before administration. Since it is a single use vial and does not contain any preservative, the drug should be used within 4 hours of withdrawal from the vial. Do not mix liposomal cytarabine with any other medicinal products. Do not dilute the suspension.

8.7.3 Application

Aqueous cytarabine will be injected slowly intraventricularly at an age-dependent dose of 16-30 mg twice a week for two weeks out of every four weeks alternating with intraventricular etoposide.
Liposomal cytarabine will be injected slowly intraventricularly at a dose of 25-50 mg every 4 weeks (+/- 3 days), alternating with intraventricular etoposide. Concomitantly with liposomal cytarabine, intravenous or oral dexamethasone (approximately 0.15 mg/kg) will be administered twice daily for a total of three to five days as a routine measure to avoid arachnoiditis. An appropriate GI tract protection, e.g. a proton pump inhibitor, should be administered as per institutional guidelines.

8.7.4 Pharmacology

Cytarabine is a cell-cycle phase specific antineoplastic agent, affecting cells only during the S-phase of cell division. Intracellularly, cytarabine is converted into cytarabine-5'-triphosphate (Ara-CTP), which is the active metabolite. It appears that ara-CTP acts primarily through inhibition of DNA synthesis. Incorporation into DNA and RNA may also contribute to cytarabine cytotoxicity. Cytarabine is cytotoxic to a wide variety of proliferating mammalian cells in culture.

8.7.5 Frequencies of Toxicities

	Common<br>Happens to 21-100 patients out of every 100	Occasional<br>Happens to 5-20 patients out of every 100	Rare<br>Happens to <5 patients out of every 100
Nervous System Disorders			
			Neuritis neural toxicity
			Headache
			Cerebral dysfunction
			Cerebellar dysfunction
			Personality changes
			Somnolence
			Coma
			Peripheral motor neuropathy
			Peripheral sensory neuropathy
General Disorders			
		Nausea	
		Fever	
			Dizziness
Gastrointestinal Disorders			
		Vomiting	
Infections and Infestations			
		Bacterial infection	

8.7.6 Method of Procurement
Aqueous cytarabine is commercially available and will be prelabeled by pharmacy as per institutional standards (according to national law). Drug Batch Numbers and expiry date will be documented in the study drug accountability. Used and unused vials will be checked by the study monitor and thereafter disposed of as per local practice.

8.8 Intraventricular etoposide

8.8.1 Description
Etoposide (also commonly known as VP-16) is a semisynthetic derivative of podophyllotoxin used in the treatment of certain neoplastic diseases. It is 4'- demethylepipodophyllotoxin 9-[4,6-0-(R)-ethylidene-(beta)-D-glucopyranoside]. It is very soluble in methanol and chloroform, slightly soluble in ethanol, and sparingly soluble in water and ether. It is made more miscible with water by means of organic solvents. It has a molecular weight of 588.58 g/mol and a molecular formula of $C_{29}H_{32}O_{13}$.

8.8.2 Formulation
See above (8.5.2). Use a concentration of 0.1 mg/mL etoposide diluted in 0.9% Sodium Chloride Intravenous Infusion BP.

8.8.3 Application
Etoposide will be given intraventricular at a dose of 0.5 mg (0.25 mg in infants <1 year) × 5 d every 4 weeks (+/- 3 days), alternating with cytarabine.

8.8.4 Pharmacology
Ventricular etoposide concentration decreased in a biexponential fashion. The highest concentrations were measured 0.25 hours after intraventricular etoposide administration. Following administration, significant cytotoxic levels of total etoposide were detected for 24 hours, with an elimination half-life of 7.65 hours. Following an intraventricular administration of 0.5 mg etoposide for 5 consecutive days, no drug accumulation was observed (Fleischhack, 2001).

8.8.5 Frequencies of Toxicities

	Common Happens to 21-100 patients out of every 100	Occasional Happens to 5-20 patients out of every 100	Rare Happens to <5 patients out of every 100
Transient headache			5%
Meningitis			3%
Vomiting			2%
Confusion			2%
Transient coma			2%
Seizure			2%
Hyponatraemia			2%

8.8.6 Method of Procurement
Etoposide is commercially available and will be prelabeled by pharmacy as per institutional standards (according to national law). Drug Batch Numbers and expiry date will be

documented in the study drug accountability. Used and unused vials will be checked by the study monitor and thereafter disposed of as per local practice.

9 Ethical section

9.1 Toxicity, inconveniences and risks for subjects

All patients enrolled in this study will receive treatment for therapeutic reasons. Relapse of a brain tumor is a severe and life-threatening disease. Death from tumor progression is typically the natural course of a relapsed / progressive brain tumor and is not considered a side effect of this trial treatment.

9.2 Toxicity / Safety precautions

The responsible physicians will be continuously aware about medication, new diagnostic findings, the course and the state of the disease of the patient. All therapeutic measures to optimize the benefit to patients will be paramount and will not be affected by study procedures. Adverse events will be followed until they have resolved or improved.

Safety variables to be assessed will include physical and neurological examinations, laboratory evaluations, vital signs and adverse events. Monitoring for adverse events will be performed at each visit and during follow-up.

9.3 Adverse Events (AE)

Adverse event = any adverse change experienced by the patient in the course of this trial, irrespective of a correlation with the trial medication. Signs and symptoms considered as lack of efficacy (tumor progression) will not be considered as an adverse event. All adverse events occurring after signing the informed consent and starting before follow-up visit of the last cycle are to be recorded on the adverse event pages of the eCRF. Periodic analysis of aggregate data will be performed at least 6-monthly, and in case of increased rate of occurrence or increase of severity of such events, a timely report will be submitted to all regulatory authorities of the countries where the study is conducted. After occurrence of adverse events, the patient must receive appropriate therapy. The patient will remain under medical supervision until all adverse events have resolved or improved to baseline. The investigator shall establish a possible correlation between an adverse event and the trial substance(s) on the basis of the following considerations:

9.3.1 Causal Relationship of Adverse Events

The investigator shall establish a possible correlation between an adverse event and the trial substance(s) on the basis of the following considerations:

**not related:**<br>Adverse events which are clearly and incontrovertibly due to extraneous causes (disease, environment etc.).
**unlikely:**<br>Those adverse events which are judged to be unrelated to the test drug. An adverse event may be considered as unlikely related if or when (must have two points positive):<br>• it does not follow a reasonable temporal sequence from administration of the test drug<br>• it could readily have been produced by the patient's clinical state, environmental or toxic factors, or other modes of therapy administered to the patient<br>• it does not follow a known pattern of response to the test drug<br>• it does not reappear or worsen when the drug is re-administered
**possible:**<br>Those adverse events for which a connection with the test drug administration appears unlikely but cannot be ruled out with certainty. An adverse experience may be considered as possibly related if or when (must have all points positive):<br>• it follows a reasonable temporal sequence from administration of the drug<br>• it could not readily have been produced by the patient's clinical state, environmental or toxic factors, or other modes of therapy administered to the patient<br>• it follows a known pattern of response to the test drug
**probable:**<br>This category applies to those adverse events which are felt with a high degree of certainty to be related to the test drug. An adverse event may be considered as probably related if or when (must have all points positive):<br>• it follows a reasonable temporal sequence from administration of the drug<br>• it cannot be reasonably explained by the known characteristics of the patient's clinical state, environmental or toxic factors, or other modes of therapy administered to the patient<br>• it disappears or decreases on cessation or reduction in dose. There are important exceptions when an adverse event does not disappear upon discontinuation of the drug, yet drug-relatedness clearly exists (for example: bone marrow depression, fixed drug eruptions, tardive dyskinesia)<br>• it follows a known pattern of response to the test drug
**definitive:**<br>This category applies to those adverse events which the investigator feels are incontrovertibly related to the test drug. An adverse event may be assigned to an attribution of definitively related if or when (must have all points positive):<br>• it follows a reasonable temporal sequence from administration of the drug<br>• it cannot be reasonably explained by the known characteristics of the patient's clinical state, environmental or toxic factors, or other modes of therapy administered to the patient<br>• it disappears or decreases on cessation or reduction in dose and recurs with re-exposure to drug. (Note: this is not to be construed as requiring re-exposure of the patient, however, a category of definitely related can only be used when recurrence is observed)<br>• it follows a known pattern of response to the test drug

Withdrawal from the trial and therapeutic measures shall be at the discretion of the clinical investigator. A full explanation for withdrawal from the trial will be made on the appropriate Case Report Form.

9.3.2 Grading of Adverse Events

The grading of adverse events will be based upon the National Cancer Institute Common Terminology Criteria for Adverse Events (Version 4.0).

9.3.3 Intensity of Adverse Events

For this study, the intensity of an AE will be rated according to the following definitions:

**Mild:**<br>Symptoms barely noticeable to the patient, does not influence performance of functioning;
**Moderate:**<br>Symptoms of a sufficient intensity to make the patient uncomfortable, performance of daily activities influenced;
**Severe:**<br>Symptoms causes severe discomfort. May be of such intensity that the patient cannot continue in the study.

9.3.4 Outcome of Adverse Events

The assessment of the outcome of an adverse event is based on following considerations:

1 = Resolved
2 = Resolved with sequelae
3 = Ongoing
4 = Unknown
5 = Death

9.3.5 Reporting of Adverse Events

The investigator will enter time, occurrence, duration, grading, intensity and a possible causal relationship with the trial substance as well as measures / consequences in detail in the source records and into the eCRF. Abnormal laboratory values corresponding to grade 1 or 2 without clinical symtpoms should not be recorded as AEs.

9.4 Serious Adverse Events (SAEs)

SAE is any experience that suggests a significant hazard, contraindication, side effect, or precaution. It is an AE occurring any time during the entire duration of the study that, at any dose of investigational treatment,

- Results in death,
- Is immediately life-threatening,
- Requires inpatient hospitalization or prolongation of existing hospitalization,
- Results in persistent or significant disability/incapacity,
- Is a congenital anomaly/birth defect, or
- Is medically significant or requires intervention to prevent one or other of the outcomes listed above.

All SAEs occurring after signing the informed consent and starting before a period of 30 days after the last study drug administration are to be reported and recorded on the adverse event pages of the eCRF. Planned hospitalisation due to a pre-existing condition will not be regarded as an SAE. Hospitalisation for treatment or any diagnostic procedures, as well as hospitalisation for safety precautions as per institutional guidelines, will not be considered as an SAE. Expected hematological toxicity CTCAE°1-4, antibiotic treatment of CTCAE°1-3 infections, or other expected toxicity CTCAE°1-3 is not to be reported on an SAE form.

9.4.1 Reporting of Serious Adverse Events (SAEs)

Irrespective of their cause, any SAEs must be immediately reported (within 24 hours of observation or notification of an SAE). The investigator or the investigators designee must fax a complete SAE Reporting Form to the sponsor (+43 1 40400 61028) and indicate if it’s suspected as SUSAR (Suspected unexpected serious adverse reaction). All SAEs must also be recorded on the appropriate Adverse Events pages in the eCRF.

Death and life-threatening events as well as suspected SUSARs should be reported immediately by telephone to Dr. Peyrl (+43 676 7031912) or Dr. Slavc (+43 676 3009204) and the completed SAE form should follow by fax (+43 1 40400 61028) within 24 hours.

It is the local investigators obligation to relay the information to the Ethics Committees, institutional review boards and responsible authorities, according to the local regulations and institutional policies.

Any information about an SAE which changes or adds information to the initial SAE form has to be reported with a follow-up SAE report form to the sponsor within 72 hours.

SAEs considered as related to study drug will be reported until 6 months after the last dose of study treatment. Any SAEs must be followed up until final resolution.

9.5 Unexpected Outcomes

All unexpected study outcomes, including any beneficial findings of benefit in a smaller size, or other unexpected results, will be reported following study completion. Any unanticipated safety concern arising during the course of the study will be submitted on an expedited basis to the respective regulatory authorities.

9.6 Contact persons for emergencies

Three physicians, familiar with the study protocol and the course of the study, will be designated as contact persons for emergency cases. Phone numbers of the contact persons have to be stated explicitly in the informed consent or as per institutional guidelines.

9.7 Risk/benefit assessment

The prognosis of recurrent or progressive medulloblastoma is bleak despite intensive therapeutic approaches. Antiangiogenic therapy inhibits vascular formation, thereby preventing tumor progression indirectly. The major risks of antiangiogenic therapy used in this protocol such as infection are comparable to conventional chemotherapy. In contrast to conventional chemotherapy, antiangiogenic therapy is given mainly orally and is performed in an outpatient setting.

In summary, the potential benefit of the study clearly outweighs the potential risks.

9.8 Informed consent

It is the responsibility of the investigator to obtain written informed consent from the patients and/or parents of each patient participating in the trial, after oral and written explanation of the aims, methods, benefits and potential hazards of the trial. Patients below the age of eight years will be informed orally according to their age, patients between eight and fourteen years receive a special children's informed consent, and patients older than fourteen years receive the parent's informed consent. Institutional guidelines can be used for consent and assent procedures. The consent must be obtained before any trial–specific procedures are performed on the patient unless the trial procedures are being performed as part of routine care. It must be made completely and unambiguously clear to the patients and / or parents that they are free to refuse to participate in the trial, or withdraw their consent at any time and for any reason, without incurring any penalty or withholding of treatment on the part of the investigator. Signed informed consent forms must be kept on file by the investigator and documented in the eCRF.

9.9 Ethical and legal aspects

The investigator will ensure that this trial is conducted in full conformance with the principles of the Declarations of Helsinki (1964), Tokyo (1975), Venice (1983), Hongkong (1989), Somerset West (1996), Edinburgh (2000), Tokyo (2004), Seoul (2008). Furthermore, it is the responsibility of the investigator to ensure that the trial is performed in accordance with the Guidelines of Good Clinical Practice of the European Commission and with the country specific regulations according to the local requirements concerning clinical studies (ICH/CPMP/135/95 Step 4 errata), and according to the Directive (2001/20/EC) of the European Parliament and of the Council of 4 April 2001.

The trial will be submitted to the Ethics Committee for approval. For trials taking longer than one year, an annual status report is provided to the Ethics Committee. If any modifications become necessary or desirable, these will be documented in writing; major changes require the approval of all investigators and the local Ethics Committee. The amendments, Annual Safety Update Reports and the End of Trial Report will be distributed to the local investigators by the sponsor. It is the local investigators obligation to relay information to the

local Ethics Committee, Institutional Review Boards and responsible authorities, according to their own interpretations of the local regulations and their own institutional policies.

9.10 Withdrawal criteria
Patients or parents may withdraw their consent to participate in the trial without giving any reason.
The investigator may discontinue study medication prematurely for any of the following reasons:

- Occurrence of adverse events or inter-current diseases
- Occurrence of exclusion criteria
- For other medical reasons
- Rapid tumor progression leading to rapid deterioration of the patient's clinical condition (assessed by the treating physician and the principal investigator)

Rapid tumor progression with decline of neurological functions and general physical condition (i.e. if the treating physician estimate that the treatment has had no significant effect on tumor growth) may lead to discontinuation of study drug administration. The patient may receive further anti-tumor treatments, but will still be evaluated, in order to assess the outcome variables of OS and PFS.

The following reasons oblige the investigator to take a patient off the study medication:

- Upon patient's or parents' request
- In case of intolerable adverse events or intercurrent diseases which the investigator deems to be unacceptable

Any patient discontinuing the treatment per protocol will remain under medical supervision until discharge is considered medically acceptable.
The aspired sample size of 40 patients in stratum I and 30 patients in stratum II and III contain an expected dropout rate of approximately 5 percent (n=2).

9.11 Premature termination of the trial (stopping rules)
The trial will be terminated prematurely in any of the following cases:

- If adverse events occur, which are so serious that the resulting risk-benefit ratio becomes unacceptable
- If the number of dropouts is so large that proper conclusion of the trial is no longer a realistic possibility
- If the results of parallel clinical trials reveal unacceptable risks
- Attempted or proven fraud
- Poor quality of data

Premature termination of the trial must be documented.

10 Administrative section

10.1 Documentation

Prior to the enrolment of any subject all documents regarding to ICH-GCP E6 (R1) in Europe and local regulations must be available in the investigator's study file.
This also applies to those subjects who fail to complete the study. If a subject withdraws from the study, the reason must be noted on the eCRF. Case report forms are to be completed on an on-going basis. The forms will include the study number, study dates of the patient, records of vital signs, medical history or examinations, laboratory results, and any adverse events. Adverse events will be recorded on separate forms as source data in native language. Suspected unexpected serious adverse reactions (SUSARs) will be reported to the responsible authorities.
eCRF entries and corrections will only be performed by study site staff, authorized by the investigator.
The subjects will be monitored and treated throughout the course of the trial by the designated team.

10.2 Quality control and quality assurance

The designated monitor will contact and visit the investigator regularly and will be allowed to have access to all source documents needed to verify the entries in the eCRFs and other protocol-related documents provided that subject confidentiality is maintained in agreement with local regulations. It will be the monitor's responsibility to inspect the eCRFs at regular intervals throughout the study, to verify the adherence to the protocol and the completeness, consistency and accuracy of the data being entered on them. The monitoring standards require full verification for the presence of informed consent, adherence to the inclusion/exclusion criteria, documentation of SAEs and the recording of the main efficacy, safety, and tolerability endpoints.
To be GCP compliant at least 3 monitoring visits are scheduled. An initiation visit, one routine visit and a final visit after the last patient has finished the study. The monitor will be working according to SOPs and will provide a GCP-compliant monitoring report after each visit for the sponsor. Depending on the quality of the data, additional monitoring visits will be necessary according to the sponsor's discretion. The investigator will cooperate with the CRA to ensure that any identified discrepancies are resolved.
Upon request, the investigator will make all study-related source data and records available to a qualified quality assurance auditor mandated by the sponsor or to CA inspectors. The main purpose of an audit or inspection is to confirm that the rights and welfare of the subjects have been adequately protected, and that all data relevant for assessment of safety and efficacy of the investigational product have appropriately been reported to the sponsor.

10.3 Publication of study results

Publication of study results from this investigation in an appropriate medical journal will be anticipated. At least 2 weeks before submission the manuscript will be circulated to all parties involved.

10.4 Protocol amendment

If any modifications become necessary or desirable, these will be documented in writing; major changes require the approval of all investigators and the local Ethics Committee.

10.5 Finance and insurance

During their participation in the clinical trial the patients will be insured as defined by legal requirements or institutional guidelines. The principal investigator of the clinical trial will receive a copy of the insurance conditions of the patients' insurance for European sites.
Details on the existing patients' insurance are given in the patient information sheet.

10.6 Error correction

For error corrections in source data and study documents, the investigator or an authorised clinical staff member will cross out the part to be corrected (no correction fluid!) with one line, so that the incorrect data is still readable, and then entering the correct data, initialling and dating the change.

10.7 Storage of study documents

All correspondence relating to this clinical trial should be kept in appropriate file folders. Records of subjects, source documents and informed consent forms pertaining to the trial must be kept on the Investigator's File. Records must be retained as defined by legal requirements or institutional guidelines.
According to the European Community record storage is required for 15 years.
If an investigator leaves, withdraws or retires, the responsibility for maintaining the records may be transferred to another person who will accept the responsibility.

11 Study schedule for patients

**Timepoints**	**T_{-1}**	**T_1**											
Week	-1	1	2	3	4	5	6	7	8	9	10	11	12	13
Date														
Informed consent	X													
MRI	X													X
Quality of life	X													
Performance status	X													
CTCAE[3]	X													X
Basic patient data	X													
Former therapy	X													
Medical history	X	X		X		X		X		X		X		X
Physical examination	X	X		X		X		X		X		X		X
Neurological examination	X	X		X		X		X		X		X		X
Blood count[4]	X	X	X	X	X	X	X	X	X	X	X	X	X	X
Blood chemistry[4]	X	X		X		X		X		X		X		X
Dipstick urinalysis (proteinuria)		X		X		X		X		X		X		X
Hormones/immunology	X											X		X
Pregnancy test	(X)[1]					(X)				(X)				(X)
Tumor/angiogenic markers (optional)	X[2]		X[2]			X[2]								X[2]
Nerve conduction velocity	(X)													(X)
Bone age	X													
Bevacizumab		X		X		X		X		X		X		X
Thalidomide		X	X	X	X	X	X	X	X	X	X	X	X	X
Celecoxib		X	X	X	X	X	X	X	X	X	X	X	X	X
Fenofibrate		X	X	X	X	X	X	X	X	X	X	X	X	X
Etoposide		X	X	X				X	X	X				X
Cyclophosphamide					X	X	X				X	X	X	
Etoposide IT		X				X				X				X
Aqueous cytarabine IT				X	X			X	X			X	X	

[1] <24 hours before starting thalidomide

[2] Serum, plasma, CSF and urine will be collected for determination of tumor/angiogenic markers before surgery and/or start of antiangiogenic treatment, one week and one month after start of treatment and at each 12-weekly MRI evaluation

[3] continuous monitoring

[4] can be performed by outside laboratories

**Timepoints**											**T_{24-28}**	
Week	14	15	16	17	18	19	20	21	22	23	24	25
Date												
Informed consent												
MRI											X[5]	X[5]
Quality of life												X
Performance status												X
CTCAE[3]												X
Basic patient data												
Former therapy												
Medical history		X		X		X		X		X		X
Physical examination		X		X		X		X		X		X
Neurological examination		X		X		X		X		X		X
Blood count[4]	X	X	X	X	X	X	X	X	X	X	X	X
Blood chemistry[4]		X		X		X		X		X		X
Dipstick urinalysis (proteinuria)		X		X		X		X		X		X
Hormones/immunology												X
Pregnancy test				(X)				(X)				(X)
Tumor/angiogenic markers (optional)												X
Nerve conduction velocity												(X)
Bone age												
Bevacizumab		X		X		X		X		X		X
Thalidomide	X	X	X	X	X	X	X	X	X	X	X	X
Celecoxib	X	X	X	X	X	X	X	X	X	X	X	X
Fenofibrate	X	X	X	X	X	X	X	X	X	X	X	X
Etoposide	X	X				X	X	X				X
Cyclophosphamide			X	X	X				X	X	X	
Etoposide IT				X				X				X
Aqueous cytarabine IT		X	X			X	X			X	X	

[3] continuous monitoring
[4] can be performed by outside laboratories
[5] MRI after 6 months has to be performed between week 24 – 28

**Timepoints**	**T_{24-28}**											
Week	26	27	28	29	30	31	32	33	34	35	36	37
Date												
MRI	X[5]	X[5]	X[5]									X
CTCAE[3]												X
Medical history		X		X		X		X		X		X
Physical examination		X		X		X		X		X		X
Neurological examination		X		X		X		X		X		X
Blood count[4]	(X)	X	(X)	X	(X)	X	(X)	X	(X)	X	(X)	X
Blood chemistry[4]		X		X		X		X		X		X
Dipstick urinalysis (proteinuria)		X		X		X		X		X		X
Hormones/immunology												X
Pregnancy test				(X)				(X)				(X)
Tumor/angiogenic markers (optional)												X
Nerve conduction velocity												(X)
Bone age												
Bevacizumab		X		X		X		X		X		X
Thalidomide	X	X	X	X	X	X	X	X	X	X	X	X
Celecoxib	X	X	X	X	X	X	X	X	X	X	X	X
Fenofibrate	X	X	X	X	X	X	X	X	X	X	X	X
Etoposide*	(X)	(X)				(X)	(X)	(X)				(X)
Cyclophosphamide*			(X)	(X)	(X)				(X)	(X)	(X)	
Etoposide IT°				(X)				(X)				(X)
Aquous cytarabine IT°		(X)	(X)			(X)	(X)			(X)	(X)	

[3] continuous monitoring

[4] can be performed by outside laboratories

* Oral etoposide and cyclophosphamide should be administered for a minimum of 6 months and continuation should take into account the long term toxicity of this drug (for example secondary leukemia).

° Intervals of intraventricular therapy may be extended after 6 months if no signs of leptomeningeal dissemination are present.

**Timepoints**												
Week	38	39	40	41	42	43	44	45	46	47	48	49
Date												
MRI												X
CTCAE[3]												X
Medical history		X		X		X		X		X		X
Physical examination		X		X		X		X		X		X
Neurological examination		X		X		X		X		X		X
Blood count[4]	(X)	X	(X)	X	(X)	X	(X)	X	(X)	X	(X)	(X)
Blood chemistry[4]		X		X		X		X		X		X
Dipstick urinalysis (proteinuria)		X		X		X		X		X		X
Hormones/immunology										X		X
Pregnancy test				(X)				(X)				
Tumor/angiogenic markers (optional)											X	X
Nerve conduction velocity											(X)	(X)
Bone age												X[#]
Bevacizumab		X		X		X		X		X		X
Thalidomide	X	X	X	X	X	X	X	X	X	X	X	X
Celecoxib	X	X	X	X	X	X	X	X	X	X	X	X
Fenofibrate	X	X	X	X	X	X	X	X	X	X	X	X
Etoposide*	(X)	(X)				(X)	(X)	(X)				(X)
Cyclophosphamide*			(X)	(X)	(X)				(X)	(X)	(X)	
Etoposide IT°				(X)				(X)				(X)
Aqueous cytarabine IT°		(X)	(X)			(X)	(X)			(X)	(X)	

[3] continuous monitoring

[4] can be performed by outside laboratories

* Oral etoposide and cyclophosphamide should be administered for a minimum of 6 months and continuation should take into account the long term toxicity of this drug (for example secondary leukemia).

° Intervals of intraventricular therapy may be extended after 6 months if no signs of leptomeningeal dissemination are present.

[#] only week 49 (+/- 2 weeks) and at least once per year (+/- 1 month) during follow-up

**Timepoints**												
Week	50	51	52									
Date												
MRI												
CTCAE[3]												
Medical history		X										
Physical examination		X										
Neurological examination		X										
Blood count[4]	(X)	X	(X)									
Blood chemistry[4]		X										
Dipstick urinalysis (proteinuria)		X										
Hormones/immunology												
Pregnancy test												
Tumor/angiogenic markers (optional)												
Nerve conduction velocity												
X-ray left hand												
Bevacizumab		X										
Thalidomide	X	X	X									
Celecoxib	X	X	X									
Fenofibrate	X	X	X									
Etoposide*	(X)	(X)										
Cyclophosphamide*			(X)									
Etoposide IT°												
Aqueous cytarabine IT°		(X)	(X)									

[3] continuous monitoring

[4] can be performed by outside laboratories

* Oral etoposide and cyclophosphamide should be administered for a minimum of 6 months and continuation should take into account the long term toxicity of this drug (for example secondary leukemia).

° Intervals of intraventricular therapy may be extended after 6 months.

[#] only week 49 (+/- 2 weeks) and at least once per year (+/- 1 month) during follow-up

12 References

1. Ahmed et al. PPARs and their metabolic modulation: new mechanisms for transcriptional regulation?. J Intern Med (2007) vol. 262 (2) pp. 184-98
2. Alvarez-Soria et al. Long-term NSAID treatment directly decreases COX-2 and mPGES-1 production in the articular cartilage of patients with osteoarthritis. Osteoarthr Cartil (2008) vol. 16 (12) pp. 1484-93
3. André and Pasquier. For cancer, seek and destroy or live and let live?. Nature (2009) vol. 460 (7253) pp. 324
4. André et al. Metronomic etoposide/cyclophosphamide/celecoxib regimen given to children and adolescents with refractory cancer: a preliminary monocentric study. Clin Ther (2008) vol. 30 (7) pp. 1336-40
5. Antony et al. A retrospective analysis of recurrent intracranial ependymoma. Pediatr Blood Cancer. (2014) vol 61 (7) pp.1195-201.
6. Ashley et al. Response of recurrent medulloblastoma to low-dose oral etoposide. J Clin Oncol (1996) Jun vol 14(6) pp. 1922-7
7. Asou et al. Long-term remission in an elderly patient with mantle cell leukemia treated with low-dose cyclophosphamide. Am J Hematol (2000) vol. 63 (1) pp. 35-7
8. Attarbaschi, personal communication: Treatment and outcome in children and adolescents with CNS-positive mature B-NHL – pattern of CNS disease and bone marrow involvement matter for outcome. A Berlin-Frankfurt-Muenster-Group report
9. Bello et al. Low-dose chemotherapy combined with an antiangiogenic drug reduces human glioma growth in vivo. Cancer Res (2001) vol. 61 (20) pp. 7501-6
10. Bergers and Hanahan. Combining antiangiogenic agents with metronomic chemotherapy enhances efficacy against late-stage pancreatic islet carcinomas in mice. Cold Spring Harb Symp Quant Biol (2002) vol. 67 pp. 293-300
11. Berland M et al. Sustained Complete Response to Metronomic Chemotherapy in a Child with Refractory Atypical Teratoid Rhabdoid Tumor: A Case Report. Front Pharmacol. (2017) vol 8 pp. 792.
12. Bingham et al. Efficacy and safety of etoricoxib 30 mg and celecoxib 200 mg in the treatment of osteoarthritis in two identically designed, randomized, placebo-controlled, non-inferiority studies. Rheumatology (Oxford) (2007) vol. 46 (3) pp. 496-507
13. Bishop-Bailey. Peroxisome proliferator-activated receptors in the cardiovascular system. Br J Pharmacol (2000) vol. 129 (5) pp. 823-34
14. Blann et al. Plasma vascular endothelial growth factor and its receptor Flt-1 in patients with hyperlipidemia and atherosclerosis and the effects of fluvastatin or fenofibrate. Am J Cardiol (2001) vol. 87 (10) pp. 1160-3
15. Bomgaars et al. Phase I trial of intrathecal liposomal cytarabine in children with neoplastic meningitis. J Clin Oncol (2004) vol. 22 (19) pp. 3916-21
16. Bouffet et al. Improving survival in recurrent medulloblastoma: earlier detection, better treatment or still an impasse?. Br J Cancer (1998) vol. 77 (8) pp. 1321-6
17. Browder et al. Antiangiogenic scheduling of chemotherapy improves efficacy against experimental drug-resistant cancer. Cancer Res (2000) vol. 60 (7) pp. 1878-86
18. Buckstein et al. High-Dose celecoxib and metronomic "low-dose" cyclophosphamide is an effective and safe therapy in patients with relapsed and refractory aggressive histology non-Hodgkin's lymphoma. Clin Cancer Res (2006) vol. 12 (17) pp. 5190-8
19. Cairo et al. Advanced stage, increased lactate dehydrogenase, and primary site, but not adolescent age ($\geq$ 15 years), are associated with an increased risk of treatment failure in children and adolescents with mature B-cell non-Hodgkin's lymphoma: results of the FAB LMB 96 study. J Clin Oncol (2012) vol 30 (4) pp. 387-93
20. Carew and Huang. Mitochondrial defects in cancer. Mol Cancer (2002) vol. 1 pp. 9
21. Chamberlain and Grafe. Recurrent chiasmatic-hypothalamic glioma treated with oral etoposide. J Clin Oncol (1995) vol. 13 (8) pp. 2072-6

22. Chamberlain. Recurrent supratentorial malignant gliomas in children. Long-term salvage therapy with oral etoposide. Arch Neurol (1997) vol. 54 (5) pp. 554-8
23. Chen and Shan. Optimal and minimax three-stage designs for phase II oncology clinical trials. Contemporary clinical trials (2008) vol. 29 (1) pp. 32-41
24. Chen et al. Plasma pharmacokinetics and urinary excretion of thalidomide after oral dosing in healthy male volunteers. Drug Metab Dispos (1989) vol. 17(4) pp. 402-405.
25. Choi et al. Feasibility of metronomic maintenance chemotherapy following high-dose chemotherapy for malignant central nervous system tumors. Pediatr. Blood Cancer (2008) vol. 50 (5) pp. 970-5
26. Choi et al. Treatment of hyperlipidemia associated with Niemann-Pick disease type B by fenofibrate. Eur J Pediatr (2006) vol. 165 (2) pp. 138-9
27. Cree et al. Insulin sensitivity and mitochondrial function are improved in children with burn injury during a randomized controlled trial of fenofibrate. Ann Surg (2007) vol. 245 (2) pp. 214-21
28. D'Amato et al. Thalidomide is an inhibitor of angiogenesis. Proc Natl Acad Sci USA (1994) vol. 91 (9) pp. 4082-5
29. Davidson et al. 21-day schedule oral etoposide in children--a feasibility study. Eur J Cancer (1993) vol. 29A (16) pp. 2223-5
30. Davies et al. Clinical pharmacokinetics and pharmacodynamics of celecoxib: a selective cyclooxygenase- 2 inhibitor. Clin Pharmacokinet (2000) vol. 38(3) pp. 225-242
31. Davies et al. Correlation between cyclooxygenase-2 expression and angiogenesis in human breast cancer. Clin Cancer Res (2003) vol. 9 (7) pp. 2651-6
32. Dellapasqua et al. Metronomic cyclophosphamide and capecitabine combined with bevacizumab in advanced breast cancer. J Clin Oncol (2008) vol. 26 (30) pp. 4899-905
33. Desjardins et al. Bevacizumab plus irinotecan in recurrent WHO grade 3 malignant gliomas. Clin Cancer Res (2008) vol. 14 (21) pp. 7068-73
34. Dunkel et al. High-dose carboplatin, thiotepa, and etoposide with autologous stem cell rescue for patients with previously irradiated recurrent medulloblastoma. Neuro-Oncology (2010) vol. 12 (3) pp. 297-303
35. Dunkel et al. High-dose carboplatin, thiotepa, and etoposide with autologous stem-cell rescue for patients with recurrent medulloblastoma. Children's Cancer Group. J Clin Oncol (1998) vol. 16 (1) pp. 222-8
36. Evans et al. The treatment of medulloblastoma. Results of a prospective randomized trial of radiation therapy with and without CCNU, vincristine, and prednisone. J Neurosurg (1990) vol. 72 (4) pp. 572-82
37. Fadul et al. A phase II study of thalidomide and irinotecan for treatment of glioblastoma multiforme. J Neurooncol (2008) vol. 90 (2) pp. 229-35
38. Finck and Kelly. Peroxisome proliferator-activated receptor alpha (PPARalpha) signaling in the gene regulatory control of energy metabolism in the normal and diseased heart. J Mol Cell Cardiol (2002) vol. 34 (10) pp. 1249-57
39. Finck et al. The cardiac phenotype induced by PPARalpha overexpression mimics that caused by diabetes mellitus. J Clin Invest (2002) vol. 109 (1) pp. 121-30
40. Fischer-Valuck BW et al. Assessment of the treatment approach and survival outcomes in a modern cohort of patients with atypical teratoid rhabdoid tumors using the National Cancer Database. Cancer (2017) vol 123 (4) pp. 682-687
41. Fleischhack et al. Feasibility of intraventricular administration of etoposide in patients with metastatic brain tumours. Br J Cancer (2001) vol. 84 (11) pp. 1453-9
42. Foeldvari et al. A prospective study comparing celecoxib with naproxen in children with juvenile rheumatoid arthritis. J Rheumatol (2009) vol. 36 (1) pp. 174-82
43. Folkman and Shing. Angiogenesis. J Biol Chem (1992) vol. 267 (16) pp. 10931-4

44. Folkman. Tumor angiogenesis: therapeutic implications. N Engl J Med (1971) vol. 285 (21) pp. 1182-6
45. Fouladi M et al. Phase II study of oxaliplatin in children with recurrent or refractory medulloblastoma, supratentorial primitive neuroectodermal tumors, and atypical teratoid rhabdoid tumors: a pediatric brain tumor consortium study. Cancer. (2006) vol 107 (9) pp 2291-7.
46. Garcia et al. Phase II clinical of bevacizumab and low-dose metronomic oral cyclophosphamide in recurrent ovarian cancer: a trial of the California, Chicago, and Princess Margaret Hospital phase II consortia. J Clin Oncol (2008) vol. 26 (1) pp. 76-82
47. García-Rodríguez and Huerta-Alvarez. Reduced risk of colorectal cancer among long-term users of aspirin and nonaspirin nonsteroidal antiinflammatory drugs. Epidemiology (2001) vol. 12 (1) pp. 88-93
48. Gatenby and Gillies. Why do cancers have high aerobic glycolysis?. Nat Rev Cancer (2004) vol. 4 (11) pp. 891-9
49. Gesundheit et al. Successful antiangiogenic therapy for neuroblastoma with thalidomide. J Clin Oncol (2007) vol. 25 (33) pp. 5321-4
50. Glade Bender et al. Phase I trial and pharmacokinetic study of bevacizumab in pediatric patients with refractory solid tumors: a Children's Oncology Group Study. J Clin Oncol (2008) vol. 26 (3) pp. 399-405
51. Glantz et al. Randomized trial of a slow-release versus a standard formulation of cytarabine for the intrathecal treatment of lymphomatous meningitis. J Clin Oncol (1999) vol. 17 (10) pp. 3110-6
52. Glode et al. Metronomic therapy with cyclophosphamide and dexamethasone for prostate carcinoma. Cancer (2003) vol. 98 (8) pp. 1643-8
53. Goetze et al. PPAR activators inhibit endothelial cell migration by targeting Akt. Biochem Biophys Res Commun (2002) vol. 293 (5) pp. 1431-7
54. Gordon et al. Phase I safety and pharmacokinetic study of recombinant human anti-vascular endothelial growth factor in patients with advanced cancer. J Clin Oncol (2001) vol. 19 (3) pp. 843-50
55. Groves et al. A North American brain tumor consortium (NABTC 99-04) phase II trial of temozolomide plus thalidomide for recurrent glioblastoma multiforme. J Neurooncol (2007) vol. 81 (3) pp. 271-7
56. Gururangan et al. Efficacy of high-dose chemotherapy or standard salvage therapy in patients with recurrent medulloblastoma. Neuro-Oncology (2008) vol. 10 (5) pp. 745-51
57. Harris et al. Reduction in the risk of human breast cancer by selective cyclooxygenase-2 (COX-2) inhibitors. BMC Cancer (2006) vol. 6 pp. 27
58. Harris et al. Similar reductions in the risk of human colon cancer by selective and nonselective cyclooxygenase-2 (COX-2) inhibitors. BMC Cancer (2008) vol. 8 pp. 237
59. Hsei et al. Effect of body weight on bevacizumab pharmacokinetics in cynomolgus monkeys and humans. Proc Am Soc Clin Oncol (2002) vol. 21 pp. (abstr 1818)
60. Hsei et al. Population pharmacokinetic (PK) analysis of bevacizumab (BV) in cancer subjects. Proc Am Soc Clin Oncol (2001) vol. 20 (69a) pp. (abstr 272)
61. Ingelfinger. Clinical Practice. the Child or Adolescent with Elevated Blood Pressure. N Engl J Med (2014) vol. 370 (24) pp. 2316–25
62. Jacoby et al. The cyclooxygenase-2 inhibitor celecoxib is a potent preventive and therapeutic agent in the min mouse model of adenomatous polyposis. Cancer Res (2000) vol. 60 (18) pp. 5040-4
63. Joki et al. Expression of cyclooxygenase 2 (COX-2) in human glioma and in vitro inhibition by a specific COX-2 inhibitor, NS-398. Cancer Res (2000) vol. 60 (17) pp. 4926-31

64. Jünger S et al. Improved risk-stratification for posterior fossa ependymoma of childhood considering clinical, histological and genetic features - a retrospective analysis of the HIT ependymoma trial cohort. Acta Neuropathol Commun (2019) vol 7(1): pp. 181
65. Kaatsch et al. Population-based epidemiologic data on brain tumors in German children. Cancer (2001) vol. 92 (12) pp. 3155-64
66. Kato et al. A case of toxic megacolon secondary to Clostridium difficile-associated diarrhea worsened after administration of an antimotility agent and molecular analysis of recovered isolates. J Gastroenterol (2007) vol. 42 (6) pp. 507-8
67. Keech et al. Effects of long-term fenofibrate therapy on cardiovascular events in 9795 people with type 2 diabetes mellitus (the FIELD study): randomised controlled trial. Lancet (2005) vol. 366 (9500) pp. 1849-61
68. Keifer et al. Inhibition of NF-kappa B activity by thalidomide through suppression of IkappaB kinase activity. J Biol Chem (2001) vol. 276 (25) pp. 22382-7
69. Kenyon et al. Effects of thalidomide and related metabolites in a mouse corneal model of neovascularization. Exp Eye Res (1997) vol. 64 (6) pp. 971-8
70. Kersten et al. Roles of PPARs in health and disease. Nature (2000) vol. 405 (6785) pp. 421-4
71. Kieran et al. A feasibility trial of antiangiogenic (metronomic) chemotherapy in pediatric patients with recurrent or progressive cancer. J Pediatr Hematol Oncol (2005) vol. 27 (11) pp. 573-81
72. Kim et al. Extended CSF cytarabine exposure following intrathecal administration of DTC 101. J Clin Oncol (1993) vol. 11 (11) pp. 2186-93
73. Kim et al. Multivesicular liposomes containing 1-beta-D-arabinofuranosylcytosine for slow-release intrathecal therapy. Cancer Res (1987) vol. 47 (15) pp. 3935-7
74. Klement et al. Continuous low-dose therapy with vinblastine and VEGF receptor-2 antibody induces sustained tumor regression without overt toxicity. J Clin Invest (2000) vol. 105 (8) pp. R15-24
75. Klement et al. Differences in therapeutic indexes of combination metronomic chemotherapy and an anti-VEGFR-2 antibody in multidrug-resistant human breast cancer xenografts. Clin Cancer Res (2002) vol. 8 (1) pp. 221-32
76. Kliewer et al. Convergence of 9-cis retinoic acid and peroxisome proliferator signalling pathways through heterodimer formation of their receptors. Nature (1992) vol. 358 (6389) pp. 771-4
77. Korones et al. Temozolomide and oral VP-16 for children and young adults with recurrent or treatment-induced malignant gliomas. Pediatr. Blood Cancer (2006) vol. 47 (1) pp. 37-41
78. Kreisl et al. Phase II trial of single-agent bevacizumab followed by bevacizumab plus irinotecan at tumor progression in recurrent glioblastoma. J Clin Oncol (2009) vol. 27 (5) pp. 740-5
79. Krzyzanowska et al. A phase II trial of continuous low-dose oral cyclophosphamide and celecoxib in patients with renal cell carcinoma. Cancer Chemother Pharmacol (2007) vol. 60 (1) pp. 135-41
80. Le Deley et al. High cumulative rate of secondary leukemia after continuous etoposide treatment for solid tumors in children and young adults. Pediatr. Blood Cancer (2005) vol. 45 (1) pp. 25-31
81. Leahy et al. Cyclooxygenase-2 inhibition by celecoxib reduces proliferation and induces apoptosis in angiogenic endothelial cells in vivo. Cancer Res (2002) vol. 62 (3) pp. 625-31
82. Lee et al. Minireview: lipid metabolism, metabolic diseases, and peroxisome proliferator-activated receptors. Endocrinology (2003) vol. 144 (6) pp. 2201-7
83. LENZ. THALIDOMIDE AND CONGENITAL ABNORMALITIES. Lancet (1962) vol. 1 (7219) pp. 45-&

84. Libby. Current concepts of the pathogenesis of the acute coronary syndromes. Circulation (2001) vol. 104 (3) pp. 365-72
85. Liu et al. The antiangiogenic effect of thalidomide on occult liver metastases: an in vivo study in mice. Journal of Gastroenterology and Hepatology (2009) vol. 24 (6) pp. 1077-81
86. López-Aguilar et al. Phase II study of metronomic chemotherapy with thalidomide, carboplatin-vincristine-fluvastatin in the treatment of brain stem tumors in children. Arch Med Res (2008) vol. 39 (7) pp. 655-62
87. Lu et al. Clinical pharmacokinetics of bevacizumab in patients with solid tumors. Cancer Chemother Pharmacol (2008) vol. 62 (5) pp. 779-86
88. Maggiora et al. An overview of the effect of linoleic and conjugated-linoleic acids on the growth of several human tumor cell lines. Int J Cancer (2004) vol. 112 (6) pp. 909-19
89. MCBRIDE. THALIDOMIDE AND CONGENITAL ABNORMALITIES. Lancet (1961) vol. 2 (721) pp. 1358-&
90. McGregor et al. Fulminant amoebic colitis following loperamide use. J Travel Med (2007) vol. 14 (1) pp. 61-2
91. Mombelli et al. Persistently increased HDL-cholesterolemia and reduced triglyceridemia in a large lipid clinic population treated with fenofibrate for 15 years or longer. International Journal of Cardiology (2009) vol. 133 (3) pp. 412-4
92. Moreira et al. Thalidomide and a thalidomide analogue inhibit endothelial cell proliferation in vitro. J Neurooncol (1999) vol. 43 (2) pp. 109-14
93. Moreira et al. Thalidomide exerts its inhibitory action on tumor necrosis factor alpha by enhancing mRNA degradation. J Exp Med (1993) vol. 177 (6) pp. 1675-80
94. Moreno-Sánchez et al. Energy metabolism in tumor cells. FEBS J (2007) vol. 274 (6) pp. 1393-418
95. Needle et al. Phase II study of daily oral etoposide in children with recurrent brain tumors and other solid tumors. Med Pediatr Oncol (1997) vol. 29 (1) pp. 28-32
96. Neve et al. Role of the peroxisome proliferator-activated receptors (PPAR) in atherosclerosis. Biochem Pharmacol (2000) vol. 60 (8) pp. 1245-50
97. Nissen SE et al. Cardiovascular Safety of Celecoxib, Naproxen, or Ibuprofen for Arthritis. N Engl J Med. (2016) vol 375 (26) pp2519-29.
98. Packer et al. Objective response of multiply recurrent low-grade gliomas to bevacizumab and irinotecan. Pediatr. Blood Cancer (2009) vol. 52 (7) pp. 791-5
99. Packer et al. Phase III study of craniospinal radiation therapy followed by adjuvant chemotherapy for newly diagnosed average-risk medulloblastoma. J Clin Oncol (2006) vol. 24 (25) pp. 4202-8
100. Packer. Brain tumors in children. Arch Neurol (1999) vol. 56 (4) pp. 421-5
101. Packer. Childhood brain tumors: accomplishments and ongoing challenges. Journal of Child Neurology (2008) vol. 23 (10) pp. 1122-7
102. Panigrahy et al. Inhibition of tumor angiogenesis by oral etoposide. Experimental and Therapeutic Medicine (2010) vol. 1 pp. 739-746
103. Panigrahy et al. PPARalpha agonist fenofibrate suppresses tumor growth through direct and indirect angiogenesis inhibition. Proc Natl Acad Sci USA (2008) vol. 105 (3) pp. 985-90
104. Partap et al. Liposomal cytarabine for central nervous system embryonal tumors in children and young adults. J Neurooncol (2011) vol. 103 (3) pp. 561-566
105. Pasquier et al. Metronomic chemotherapy: new rationale for new directions. Nat Rev Clin Oncol (2010) vol. 7 (8) pp. 455-65
106. Peyrl et al. A metronomic and targeted antiangiogenesis therapy appears to be promising in recurrent medulloblastoma - experience in 15 patients. Neuro-Oncol (2014) vol. 16 (suppl. 1) pp. 87-88
107. Peyrl et al. Antiangiogenic metronomic therapy for children with recurrent embryonal brain tumors. Pediatr. Blood Cancer (2012) vol 59 (3) pp. 511-7.

108. Peyrl et al. Pharmacokinetics and safety of intrathecal liposomal cytarabine in children aged <3 years. Clin Pharmacokinet (2009) vol. 48 (4) pp. 265-71
109. Peyrl et al. Pharmacokinetics and toxicity of intrathecal liposomal cytarabine in children and adolescents following age-adapted dosing. Clin Pharmacokinet (2014) vol 53 (2) pp. 165-73.
110. Peyrl et al. Safety of Ommaya reservoirs in children with brain tumors: a 20-year experience with 5472 intraventricular drug administrations in 98 patients. J Neurooncol (2014) vol. 120 (1) pp. 139-145
111. Pinter et al. Thalidomide in advanced hepatocellular carcinoma as antiangiogenic treatment approach: a phase I/II trial. Eur J Gastroenterol Hepatol (2008) vol. 20 (10) pp. 1012-9
112. Pizer et al. Treatment of recurrent central nervous system primitive neuroectodermal tumours in children and adolescents: results of a Children's Cancer and Leukaemia Group study. Eur J Cancer (2011) vol 47(9) pp. 1389-97
113. Poulsen et al. Bevacizumab plus irinotecan in the treatment patients with progressive recurrent malignant brain tumours. Acta Oncol (2009) vol. 48 (1) pp. 52-58
114. Pozzi and Capdevila. PPARalpha Ligands as Antitumorigenic and Antiangiogenic Agents. PPAR Research (2008) vol. 2008 pp. 906542
115. Ravens-Sieberer and Bullinger. Assessing health-related quality of life in chronically ill children with the German KINDL: first psychometric and content analytical results. Qual Life Res (1998) vol. 7 (5) pp. 399-407
116. Reismüller et al. Feasibility and tolerability of bevacizumab in children with primary CNS tumors. Pediatr. Blood Cancer (2010) vol. 54 (5) pp. 681-6
117. Robison et al. A phase II trial of a multi-agent oral antiangiogenic (metronomic) regimen in children with recurrent or progressive cancer. Pediatr. Blood Cancer (2013) Oct 4. doi: 10.1002/pbc.24794. [Epub ahead of print]
118. Rosemberg and Fujiwara. Epidemiology of pediatric tumors of the nervous system according to the WHO 2000 classification: a report of 1,195 cases from a single institution. Childs Nerv Syst (2005) vol. 21 (11) pp. 940-4
119. Rostom et al. Gastrointestinal safety of cyclooxygenase-2 inhibitors: a Cochrane Collaboration systematic review. Clin Gastroenterol Hepatol (2007) vol. 5 (7) pp. 818-28, 828.e1-5; quiz 768
120. Sabel et al. Relapse patterns and outcome after relapse in standard risk medulloblastoma: a report from the HIT-SIOP-PNET4 study. J Neurooncol. 2016 Sep;129(3):515-524.
121. Saidi et al. In vitro and in vivo effects of the PPAR-alpha agonists fenofibrate and retinoic acid in endometrial cancer. Mol Cancer (2006) vol. 5 pp. 13
122. Sampaio et al. Thalidomide selectively inhibits tumor necrosis factor alpha production by stimulated human monocytes. J Exp Med (1991) vol. 173 (3) pp. 699-703
123. Sathornsumetee et al. Tumor angiogenic and hypoxic profiles predict radiographic response and survival in malignant astrocytoma patients treated with bevacizumab and irinotecan. J Clin Oncol (2008) vol. 26 (2) pp. 271-8
124. Scatena et al. Bezafibrate induces a mitochondrial derangement in human cell lines: a PPAR-independent mechanism for a peroxisome proliferator. Chem Res Toxicol (2003) vol. 16 (11) pp. 1440-7
125. Scatena et al. Mitochondria, PPARs, and Cancer: Is Receptor-Independent Action of PPAR Agonists a Key?. PPAR Research (2008) vol. 2008 pp. 256251
126. Schrappe et al. Late MRD response determines relapse risk overall and in subsets of childhood T-cell ALL: results of the AIEOP-BFM-ALL 2000 study. Blood (2011) vol 118 pp2077-84.
127. Shih et al. High-dose chemotherapy with autologous stem cell rescue for children with recurrent malignant brain tumors. Cancer (2008) vol. 112 (6) pp. 1345-53

128. Slavc et al. Feasibility of long-term intraventricular therapy with mafosfamide (n = 26) and etoposide (n = 11): experience in 26 children with disseminated malignant brain tumors. J Neurooncol (2003) vol. 64 (3) pp. 239-47
129. Slavc et al. A Metronomic Antiangiogenic Combination Therapy Prolongs Survival for Patients with Recurrent Medulloblastoma: Experience in 20 Consecutive Patients. Pediatric Blood & Cancer (2019) vol. 66 (supp 4) pp. S58
130. Slavc et al. A metronomic antiangiogenic combination therapy may prolong survival for patients with recurrent medulloblastoma and atypical teratoid rhabdoid tumor. Neuro-Oncology (2018) vol. 20 (suppl 6) pp. 145
131. Slavc et al. Response of recurrent malignant childhood CNS tumors to a MEMMAT based metronomic antiangiogenic combination therapy varies dependent on tumor type: Experience in 71 patients. Neuro-Oncology (2018) vol. 20 suppl 2 pp. 122
132. Smith et al. Prostaglandin endoperoxide H synthases (cyclooxygenases)-1 and -2. J Biol Chem (1996) vol. 271 (52) pp. 33157-60
133. Steinbach et al. The effect of celecoxib, a cyclooxygenase-2 inhibitor, in familial adenomatous polyposis. N Engl J Med (2000) vol. 342 (26) pp. 1946-52
134. Stempak et al. A pilot pharmacokinetic and antiangiogenic biomarker study of celecoxib and low-dose metronomic vinblastine or cyclophosphamide in pediatric recurrent solid tumors. J Pediatr Hematol Oncol (2006) vol. 28 (11) pp. 720-8
135. Sterba et al. Combined biodifferentiating and antiangiogenic oral metronomic therapy is feasible and effective in relapsed solid tumors in children: single-center pilot study. Onkologie (2006) vol. 29 (7) pp. 308-13
136. Sterba et al. Second complete remission of relapsed medulloblastoma induced by metronomic chemotherapy. Pediatr. Blood Cancer (2010) vol. 54 (4) pp. 616-7
137. Stockklausner et al. Long-term remission of children with relapsed and secondary anaplastic large cell non-Hodgkin lymphoma (ALCL) following treatment with pulsed dexamethasone and low dose etoposide. Pediatr. Blood Cancer (2008) vol. 50 (1) pp. 126-9
138. Stoelting et al. Low-dose oral metronomic chemotherapy prevents mobilization of endothelial progenitor cells into the blood of cancer patients. In Vivo (2008) vol. 22 (6) pp. 831-6
139. Sung et al. Successful metronomic low-dose cyclophosphamide therapy in an older patient with advanced mucosa-associated lymphoid tissue lymphoma. Ann Hematol (2009) vol. 88 (12) pp. 1257-9
140. Tait et al. Adjuvant chemotherapy for medulloblastoma: the first multi-centre control trial of the International Society of Paediatric Oncology (SIOP I). Eur J Cancer (1990) vol. 26 (4) pp. 464-9
141. Talpur et al. Optimizing bexarotene therapy for cutaneous T-cell lymphoma. J Am Acad Dermatol (2002) vol. 47 (5) pp. 672-84
142. Tanaka et al. Ligands for peroxisome proliferator-activated receptors alpha and gamma inhibit chemically induced colitis and formation of aberrant crypt foci in rats. Cancer Res (2001) vol. 61 (6) pp. 2424-8
143. Thuillier et al. Activators of peroxisome proliferator-activated receptor-alpha partially inhibit mouse skin tumor promotion. Mol Carcinog (2000) vol. 29 (3) pp. 134-42
144. Tian and Abel. Responses of GLUT4-deficient hearts to ischemia underscore the importance of glycolysis. Circulation (2001) vol. 103 (24) pp. 2961-6
145. Tippelt S et al. Recurrent ependymoma a challenging therapy: Re-irradiation prolongs survival in failure of radical resection. Neuro-Oncology (2016) 18:iii159–iii164.
146. TomLinson et al. Comparison of in vitro activity of epipodophyllotoxins with other chemotherapeutic agents in human medulloblastomas. Br J Cancer (1991) vol. 64 (6) pp. 1051-9

147. Torres et al. Surveillance scanning of children with medulloblastoma. N Engl J Med (1994) vol. 330 (13) pp. 892-5
148. Tsujii et al. Cyclooxygenase regulates angiogenesis induced by colon cancer cells. Cell (1998) vol. 93 (5) pp. 705-16
149. Tsujii et al. Cyclooxygenase-2 expression in human colon cancer cells increases metastatic potential. Proc Natl Acad Sci USA (1997) vol. 94 (7) pp. 3336-40
150. Urbanska et al. Activation of PPARalpha inhibits IGF-I-mediated growth and survival responses in medulloblastoma cell lines. Int J Cancer (2008) vol. 123 (5) pp. 1015-24
151. Vander Heiden et al. Understanding the Warburg effect: the metabolic requirements of cell proliferation. Science (2009) vol. 324 (5930) pp. 1029-33
152. von Hoff et al. Long-term outcome and clinical prognostic factors in children with medulloblastoma treated in the prospective randomised multicentre trial HIT'91. Eur J Cancer (2009) vol. 45 (7) pp. 1209-17
153. Vredenburgh et al. Bevacizumab plus irinotecan in recurrent glioblastoma multiforme. J Clin Oncol (2007) vol. 25 (30) pp. 4722-9
154. WARBURG et al. On the metabolism of carcinoma cells. Biochem Z (1924) vol. 152 pp. 309-344
155. WARBURG. ORIGIN OF CANCER CELLS. Science (1956) vol. 123 (3191) pp. 309-314
156. Warmuth-Metz. Imaging Recommendations for the Paediatric Brain Tumour Studies. Springer International Publishing, (2017), ISBN 978-3-319-42501-6
157. Wetmore C et al. Alisertib is active as single agent in recurrent atypical teratoid rhabdoid tumors in 4 children. Neuro Oncol. (2015) vol 17 (6) pp882-8.
158. Willett et al. Direct evidence that the VEGF-specific antibody bevacizumab has antivascular effects in human rectal cancer. Nat Med (2004) vol. 10 (2) pp. 145-7
159. Yoshida et al. COX-2/VEGF-dependent facilitation of tumor-associated angiogenesis and tumor growth in vivo. Lab Invest (2003) vol. 83 (10) pp. 1385-94
160. Zacharoulis et al. Biological modification strategies following marrow ablative, high-dose chemotherapy (HDCT) with autologous hematopoietic stem cell rescue (AHSCR) for pediatric brain tumors. Pediatr. Blood Cancer (2010) vol. 54 (4) pp. 654-6
161. Zimm et al. Cytosine arabinoside cerebrospinal fluid kinetics. Clin Pharmacol Ther (1984) vol. 35 (6) pp. 826-30
162. Zuniga et al. Efficacy, safety and patterns of response and recurrence in patients with recurrent high-grade gliomas treated with bevacizumab plus irinotecan. J Neurooncol (2009) vol. 91 (3) pp. 329-36

13 MEMMAT Appendix

A - Graphical Overview

B - Performance status

C - Tanner stages

D - MRI protocol

E - Conversion of RCF (g) to RPM

F - Bevacizumab Treatment Management for Proteinuria

G - Serious Adverse Event (SAE) Report Form

H - Pregnancy Notification Form

I - Adverse Event Report Form

J – End of Therapy Report Form

K - Case Report Forms

L - Common Terminology Criteria for Adverse Events (CTCAE)
