# Supplementary material for: Sustained Survival Benefit in Recurrent Medulloblastoma by a Metronomic Antiangiogenic Regimen: A Nonrandomized Controlled Trial
Source: JAMA Oncol. 2023 Oct 26;9(12):1688–95. doi: 10.1001/jamaoncol.2023.4437 (PMC10603581; doi:10.1001/jamaoncol.2023.4437)
Supplement: Supplement 2. — eFigure 1. OS After 12 Months for Medulloblastoma Subgroup Distribution eFigure 2. Kaplan-Meier Estimates of Survival Outcomes Regarding the Number of Relapses eFigure 3. Kaplan-Meier Estimates of Survival Outcomes Regarding Intraventricular Liposomal Cytarabine (n = 17) and Aqueous Cytarabine (n = 24) eFigure 4. Kaplan-Meier Estimates for PFS of Patients That Exhibited Disease Control (NED+CR+PR+SD) eFigure 5. Kaplan-Meier Estimates for PFS of Patients That Exhibited Response (NED+CR+PR) eFigure 6. Kaplan-Meier Estimates for PFS of Patients That Showed No Progression After 12 Months of MEMMAT Treatment eTable 1. MEMMAT Regimen: Dosing Schedule eTable 2. Dose Modifications of the MEMMAT Regimen eTable 3. Results and Description of the Interim Analyses eTable 4. Responders and Non-responders by Subgroup eTable 5. All Treatment-Related AEs in the Study Population (N = 40) eTable 6. Quality of Life at the Beginning and After 6 Months of the MEMMAT Protocol [file jamaoncol-e234437-s002.pdf]

Supplementary Online Content

Peyrl A, Chocholous M, Sabel M, et al. Sustained survival benefit in recurrent medulloblastoma by a metronomic antiangiogenic regimen: a nonrandomized controlled trial. *JAMA Oncol.* Published online October 26, 2023. doi:10.1001/jamaoncol.2023.4437

eFigure 1. OS After 12 Months for Medulloblastoma Subgroup Distribution
eFigure 2. Kaplan-Meier Estimates of Survival Outcomes Regarding the Number of Relapses
eFigure 3. Kaplan-Meier Estimates of Survival Outcomes Regarding Intraventricular Liposomal Cytarabine (n=17) and Aqueous Cytarabine (n=24)
eFigure 4. Kaplan-Meier Estimates for PFS of Patients That Exhibited Disease Control (NED+CR+PR+SD)
eFigure 5. Kaplan-Meier Estimates for PFS of Patients That Exhibited Response (NED+CR+PR)
eFigure 6. Kaplan-Meier Estimates for PFS of Patients That Showed No Progression After 12 Months of MEMMAT Treatment
eTable 1. MEMMAT Regimen: Dosing Schedule
eTable 2. Dose Modifications of the MEMMAT Regimen
eTable 3. Results and Description of the Interim Analyses
eTable 4. Responders and Non-responders by Subgroup
eTable 5. All Treatment-Related AEs in the Study Population (N=40)
eTable 6. Quality of Life at the Beginning and After 6 Months of the MEMMAT Protocol

This supplementary material has been provided by the authors to give readers additional information about their work.

© 2023 Peyrl A et al. *JAMA Oncology.*

eFigure 1. OS after 12 months for medulloblastoma subgroup distribution; WNT, n=1, 0%; SHH, n=4, 25.0% ± 21.7; Group 3, n=12, 54.5% ± 15.0; Group 4, n=23, 78.3% ± 8.6; PFS progression-free survival; WNT, wingless; SHH, sonic hedgehog;

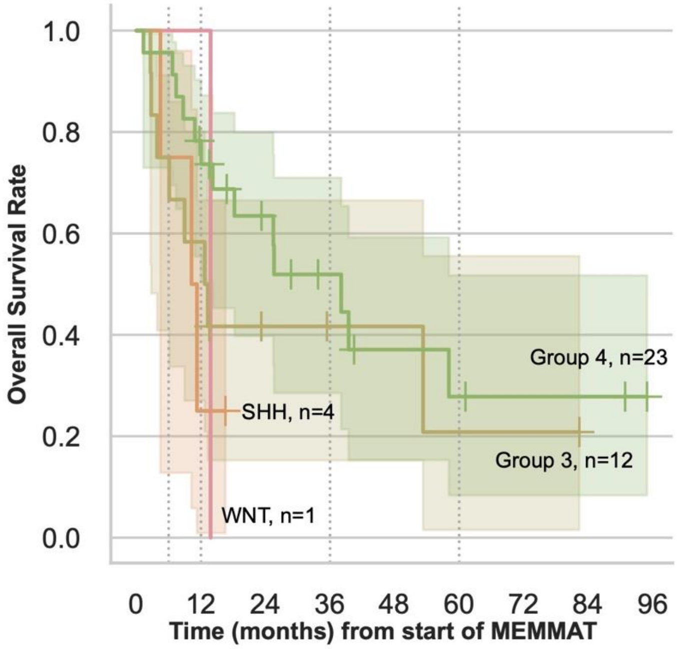

No. at Risk (Censored)

WNT	1(0)	1(0)	0(0)						
SHH	4(0)	1(0)	0(1)						
Group 3	12(0)	7(0)	3(2)	2(3)	2(3)	1(3)	1(3)	0(4)	
Group 4	23(0)	17(1)	11(4)	7(6)	4(7)	3(7)	2(8)	2(8)	0(10)

© 2023 Peyrl A et al. *JAMA Oncology.*

eFigure 2. Kaplan-Meier estimates of survival outcomes regarding the number of relapses; first relapse (n=30), 2nd/3rd relapse (n=10);

(A) PFS after 36 months: 1st relapse 28.0% ± 9.6; 2nd/3rd relapse 13.3% ± 12.0; p=0.340; p=0.814;

(B) OS after 36 months: 1st relapse 47.9% ± 10.0; 2nd/3rd relapse 30.0% ± 14.5; p=0.364; p=0.918;

A

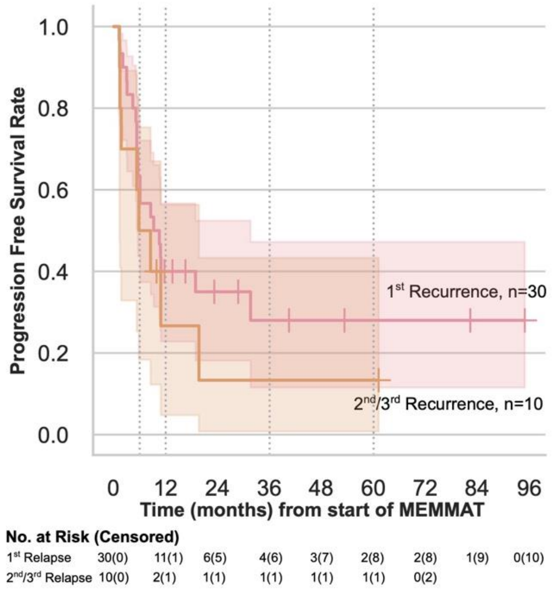

B

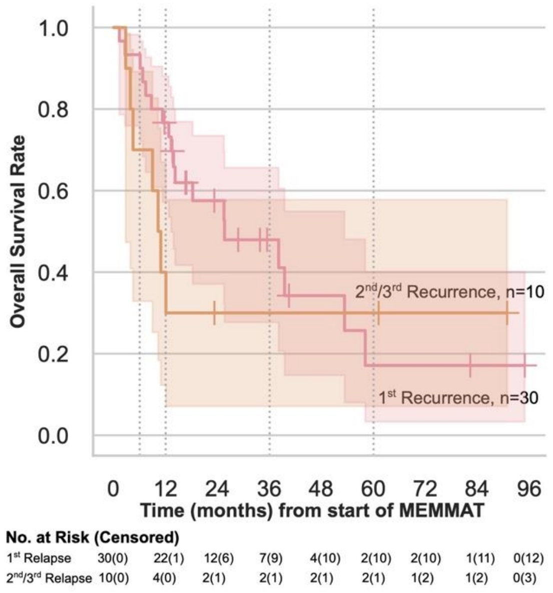

© 2023 Peyrl A et al. *JAMA Oncology.*

eFigure 3. Kaplan-Meier estimates of survival outcomes regarding intraventricular liposomal cytarabine (n=17) and aqueous cytarabine (n=24). (A) PFS after 36 months: Liposomal cytarabine 28.6% ± 11.8, aqueous cytarabine 25.0% ± 10.2; p=0.814;

B) OS after 36 months: Liposomal cytarabine 50.0% ± 12.5, aqueous cytarabine 33.3% ± 12.1; p=0.918;

A

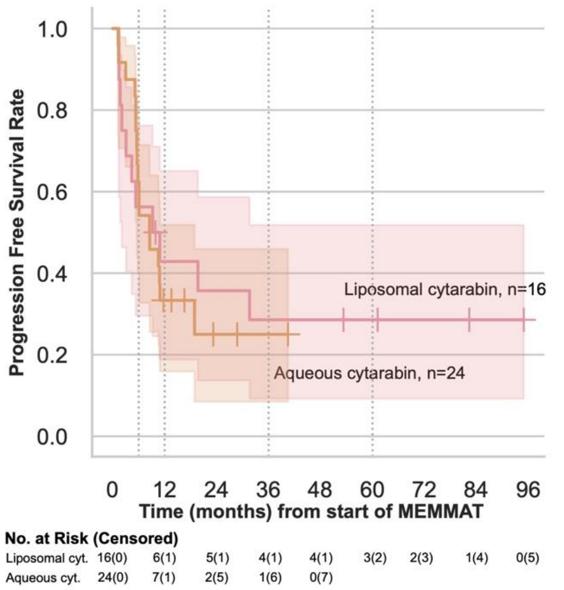

B

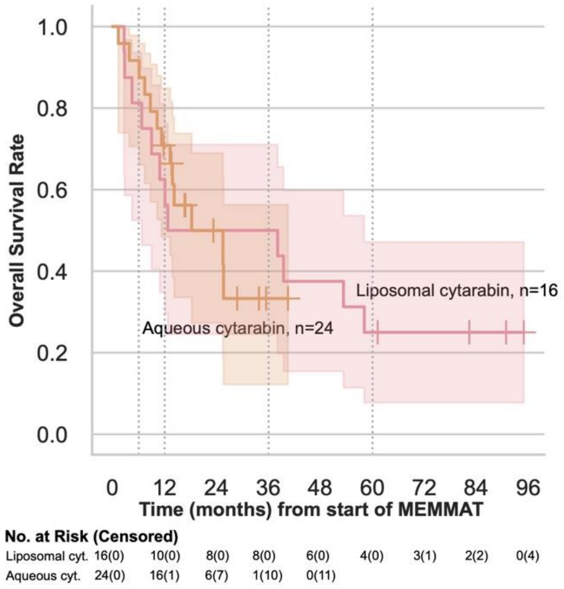

© 2023 Peyrl A et al. *JAMA Oncology.*

eFigure 4. Kaplan-Meier estimates for PFS of patients that exhibited disease control (NED+CR+PR+SD); median PFS: 31.6 (11.0-52.2) months; PFS after 60 months: 42.8% ± 12.4; PFS progression-free survival;

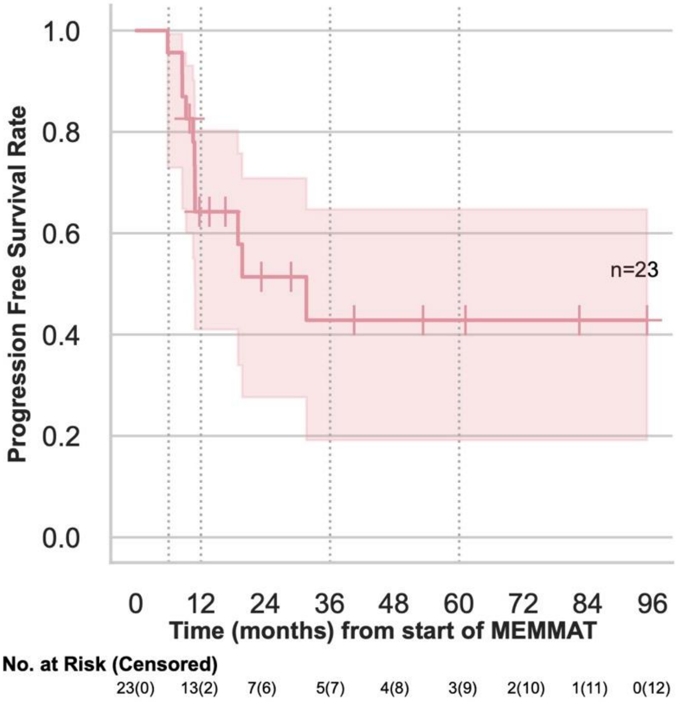

© 2023 Peyrl A et al. *JAMA Oncology.*

eFigure 5. Kaplan-Meier estimates for PFS of patients that exhibited response (NED+CR+PR); median PFS: 31.6 (11.0-52.2) months; PFS after 60 months: 49.7% ± 14.3; PFS progression-free survival;

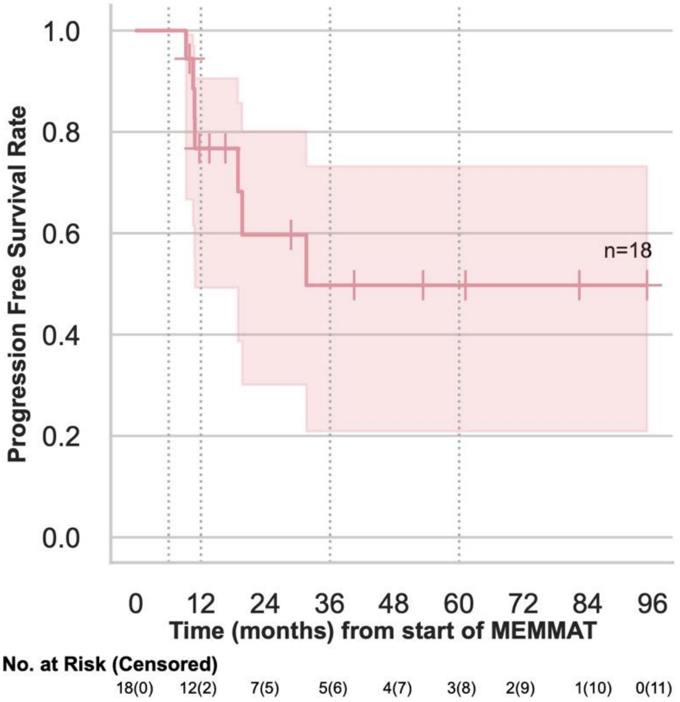

© 2023 Peyrl A et al. *JAMA Oncology.*

eFigure 6. Kaplan-Meier estimates for PFS of patients that showed no progression after 12 months of MEMMAT treatment; PFS after 60 months: 66.7% ± 16.1; PFS progression-free survival.

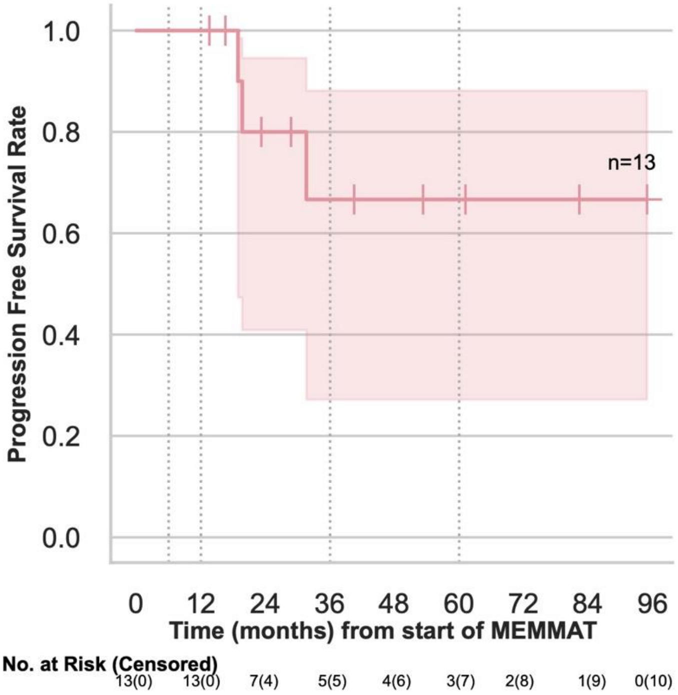

© 2023 Peyrl A et al. *JAMA Oncology.*

eTable 1. MEMMAT regimen: Dosing schedule.

**Medication**	**Dosing schedule**
**Continuous oral**	
Thalidomide	3 mg/kg/day daily
Celecoxib	Initiated twice daily in patients <10 kg at 50 mg, in patients 10-35 kg at 100 mg; patients >20 kg increased the dose to 200 mg; patients >35 kg were initiated at 200 mg and increased the dose to 300 mg, patients >50 kg escalated the dose to 400 mg
Fenofibrate	90 mg/m^2 daily
Alternating 21-day courses	
Etoposide	35-50 mg/m^2 daily for 21 days depending on anticipated bone marrow tolerance
Cyclophosphamide	2.5 mg/kg daily for 21 days
**Intravenous**	
Bevacizumab	10 mg/kg every two weeks
**Intraventricular, alternating etoposide and cytarabine every two weeks**	
Etoposide	0.5 mg on five consecutive days
Cytarabine	
Liposomal cytarabine	25 mg for children <3 years, 35 mg for children 3–9 years old and 50 mg for patients older than 9 years
Aqueous cytarabine (after withdrawal of liposomal cytarabine from the market)	30 mg twice a week; <1 year 16 mg, >1 and <2 years 20 mg, >2 and <3 years 26 mg) on days 1, 4, 8 and 11

Detailed guidelines to administer intraventricular chemotherapy via an Ommaya reservoir: Peyrl A, Chocholous M, Azizi AA, et al. Safety of Ommaya reservoirs in children with brain tumors: a 20-year experience with 5472 intraventricular drug administrations in 98 patients. *J Neurooncol.* 2014;120(1):139-45. doi: 10.1007/s11060-014-1531-1.

© 2023 Peyrl A et al. *JAMA Oncology.*

eTable 2. Dose modifications of the MEMMAT regimen.

Medication	Suggested dose modification
**Oral**	
Etoposide, cyclophosphamide	If at any time during therapy ANC falls to <1500/mm^3, then etoposide or cyclophosphamide should be reduced or held until the ANC returns to ≥1500/mm^3. Missed doses are not made up.
Thalidomide	Thalidomide should be reduced if signs of neuropathy are observed.
Celecoxib	Celecoxib should be decreased to a lower dose level or held in case of increased creatinine.
Fenofibrate	If ALT is <5x upper limit of normal, therapy with fenofibrate should be continued. If ALT is ≥5x normal, fenofibrate should held until the level is <3x normal. Fenofibrate dose should be reinitiated at 50% of the previous dose and escalated to 75% after 1 week, and then 100% after 1 week if the ALT remain <3x normal.
**Intravenous**	
Bevacizumab	Delay: Hypertension grade 3 (despite medication), proteinuria grade 3; wound healing complications;
**Intraventricular**	
Etoposide, cytarabine	In case of side effects (i.e., headache, nausea, fatigue, …), intraventricular chemotherapy should be delayed until side effects resolve.

All dose modifications should be based on the individual patient's circumstances and the physician's judgment, considering the updated SmPCs of all used drugs.

© 2023 Peyrl A et al. *JAMA Oncology.*

eTable 3. Results and Description of the Interim Analyses

**First interim analysis after 16 patients**
CR (5), PR (4), PD (7); Drop-Out (1)
**Second interim analysis after 27 patients**
NED (1), CR (6), PR (6), SD (2), PD (12)

First interim analysis:
- In the first stage, 16 patients were recruited and their response to therapy was determined:
- If one or fewer of the 16 patients would have shown a response to therapy, this study with the retention of the null hypothesis would have been terminated.
-Since at least two patients showed a response to therapy, the study was resumed and a further 11 patients were recruited into stage 2.

Second interim analysis:
- In the second stage, the response to therapy of 27 patients was determined:
- If four or fewer of the 27 patients would have demonstrated a response to therapy, this study with the retention of the null hypothesis would have been terminated.
- Since at least five patients showed a response to therapy, the study was resumed and a further 11 patients were recruited to stage 3.

Final analysis:
- If 9 or fewer of the 38 recruited patients would have shown a response to therapy, then the study would have been terminated with the retention of the null hypothesis.
- Since ten or more patients demonstrated a response to therapy, the null hypothesis was rejected and the alternative hypothesis was accepted.

© 2023 Peyrl A et al. *JAMA Oncology.*

eTable 4. Responders and non-responders by subgroup.

**Molecular subgroup at original diagnosis (n)**	**Best response (n)**
WNT (1)	PD (1)
SHH (4)	PR (1), SD (1), PD (2)
Group 3 (12)	NED (1), CR (2), SD (2), PD (7)
Group 4 (23)	NED (2), CR (4), PR (8), SD (2), PD (7)

© 2023 Peyrl A et al. *JAMA Oncology.*

eTable 5. All treatment-related AEs in the study population (n=40).

AE	Number of patients (%)					
**Hematologic**						
	**Grade 1**	**Grade 2**	**Grade 3**	**Grade 4**	**Grade 5**	**Total**
Acute myeloid leukemia MLL					1 (2.5%)	1 (2.5%)
Anemia		5 (12.5%)	5 (12.5%)			10 (25%)
Leucopenia		3 (7.5%)	2 (5%)	8 (20%)		13 (32.5%)
Lymphopenia		2 (5%)		3 (7.5%)		5 (12.5%)
Neutropenia	3 (7.5%)	2 (5%)	3 (7.5%)	14 (35%)		22 (55%)
Platelet count decreased	1 (2.5%)			4 (10%)		5 (12.5%)

**Neurologic**						
	**Grade 1**	**Grade 2**	**Grade 3**	**Grade 4**	**Grade 5**	**Total**
Cerebral Hemorrhage	1 (2.5%)					1 (2.5%)
Confusion		2 (5%)				2 (5%)
Diplopic images	3 (7.5%)	1 (2.5%)				4 (10%)
Dizziness	1 (2.5%)					1 (2.5%)
Dysarthria	1 (2.5%)	2 (5%)				3 (7.5%)
Dysesthesia	1 (2.5%)					1 (2.5%)
Fatigue	2 (5%)	2 (5%)	1 (2.5%)			5 (12.5%)
Headache	4 (10%)		2 (5%)			6 (15%)
Hearing impairment		1 (2.5%)		1 (2.5%)		2 (5%)
Nausea	3 (7.5%)					3 (7.5%)
Nystagmus	1 (2.5%)					1 (7.5%)
Papilledema	2 (5%)					2 (5%)
Paresthesia	3 (7.5%)					3 (7.5%)
Seizure	3 (7.5%)	1 (2.5%)	2 (5%)			6 (15%)
Sensomotoric polyneuropathy	1 (2.5%)					1 (2.5%)
Tremor	1 (2.5%)					1 (7.5%)
Weakness	1 (2.5%)	2 (5%)				3 (7.5%)

**Gastrointestinal**						
	**Grade 1**	**Grade 2**	**Grade 3**	**Grade 4**	**Grade 5**	**Total**
Constipation	4 (10%)					4 (10%)
Diarrhea	2 (5%)					2 (5%)
Vomiting	2 (5%)					2 (5%)

© 2023 Peyrl A et al. *JAMA Oncology.*

**Infection**						
	**Grade 1**	**Grade 2**	**Grade 3**	**Grade 4**	**Grade 5**	**Total**
Cerebellitis			1 (2.5%)			1 (2.5%)
Chemical arachnoiditis				1 (2.5%)		1 (2.5%)
Conjunctivitis	1 (2.5%)					1 (2.5%)
Febrile Neutropenia			2 (5%)			2 (5%)
Fever		1 (2.5%)				1 (2.5%)
Gingivitis		1 (2.5%)				1 (2.5%)
Infection, NOS	2 (5%)	3 (7.5%)	3 (7.5%)	1 (2.5%)		9 (22.5%)
Meningitis			2 (5%)			2 (5%)
Mucositis	1 (2.5%)		1 (2.5%)			2 (5%)
Sepsis				1 (2.5%)		1 (2.5%)
Upper respiratory tract infection		2 (5%)				2 (5%)
Urinary tract infection			1 (2.5%)			1 (2.5%)

**Laboratory**						
	**Grade 1**	**Grade 2**	**Grade 3**	**Grade 4**	**Grade 5**	**Total**
Elevated liver enzymes	3 (7.5%)		2 (5%)			5 (12.5%)
Elevated renal parameters	1 (2.5%)	1 (2.5%)				2 (5%)
Hematuria	1 (2.5%)					1 (2.5%)
Hyperuricemia	1 (2.5%)					1 (2.5%)
Hypomagnesemia		1 (2.5%)				1 (2.5%)
Hyponatremia	3 (7.5%)		3 (7.5%)			6 (12.5%)
**Various**						
	**Grade 1**	**Grade 2**	**Grade 3**	**Grade 4**	**Grade 5**	**Total**
Alopecia	3 (7.5%)					3 (7.5%)
Anorexia	2 (5%)	1 (2.5%)				3 (7.5%)
Dry eyes	1 (2.5%)					1 (2.5%)
Epistaxis			1 (2.5%)			1 (2.5%)
Hypertension		1 (2.5%)	2 (5%)			3 (7.5%)
Port a Cath revision	1 (2.5%)					1 (2.5%)
Proteinuria			2 (5%)			2 (5%)
Wound dehiscence		1 (2.5%)	1 (2.5%)			2 (5%)

© 2023 Peyrl A et al. *JAMA Oncology.*

eTable 6. Quality of Life at the beginning and after 6 months of the MEMMAT protocol.

	Self report						Parent report					
timepoint *	t0	t6mo	t0	t6mo	t0	t6mo	t0	t6mo	t0	t6mo	t0	t6mo
QoL	*high*		*Medium*		*low*		*high*		*medium*		*low*	
%	12,40	12,50	46,00	56,25	41,60	31,25	6,90	5,60	41,00	50,00	52,00	44,40

t0: start of protocol; t6mo: after 6 months

© 2023 Peyrl A et al. *JAMA Oncology.*
