# Supplementary material for: Sustained Survival Benefit in Recurrent Medulloblastoma by a Metronomic Antiangiogenic Regimen: A Nonrandomized Controlled Trial
Source: JAMA Oncol. 2023 Oct 26;9(12):1688–95. doi: 10.1001/jamaoncol.2023.4437 (PMC10603581; doi:10.1001/jamaoncol.2023.4437)
Supplement: Supplement 3. — Data Sharing Statement [file jamaoncol-e234437-s003.pdf]

Data Sharing Statement

Peyrl. Sustained Survival Benefit in Recurrent Medulloblastoma by a Metronomic Antiangiogenic Regimen. *JAMA Oncol*. Published October 26, 2023.
doi:10.1001/jamaoncol.2023.4437

Data
Data available: Yes
Data types: Deidentified participant data
How to access data: Data are available upon reasonable request.
When available: With publication

Supporting Documents
Document types: Statistical/analytic code, Informed consent form
How to access documents: Andreas.peyrl@meduniwien.ac.at
When available: With publication

Additional Information
Who can access the data: Anyone requesting the data
Types of analyses: for scientific research
Mechanisms of data availability: with a signed data access agreement
